# Unexpected Diastereomer Formation and Interconversions in Cyclohexane-1,2-diacetal Derivatization of a Glucuronic Acid Thioglycoside

**DOI:** 10.1021/acs.orglett.3c00255

**Published:** 2023-03-27

**Authors:** Fahad Ayesh Alharthi, Garrett T. Potter, Gordon C. Jayson, George F. S. Whitehead, Iñigo J. Vitórica-Yrezábal, John M. Gardiner

**Affiliations:** † Department of Chemistry, School of Natural Sciences, The University of Manchester, Oxford Road, Manchester M13 9PL, U.K.; ‡ Institute of Cancer Sciences, Faculty of Medical and Human Sciences, The University of Manchester, Manchester M20 4BX, U.K.

## Abstract

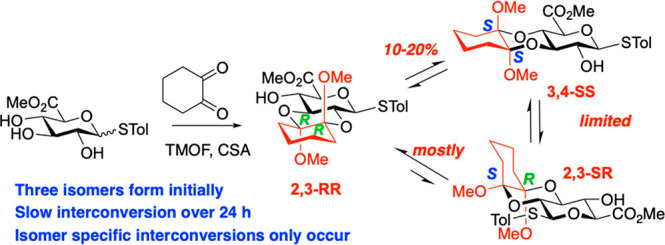

Reactions of a glucuronic acid (GlcA) β-thioglycoside with cyclohexadione show initial formation of the two anticipated all-trans decalin-type O2,O3 and O3,O4 cyclohexane-1,2-diacetals (CDAs) along with an epimer of the main O2,O3 acetal. This trans–cis isomer is then interconverted leading to higher amounts of the two all-trans products. Isomerization studies indicate slow interconversion between the all-trans CDA acetals, with only one undergoing significant interconversion with the minor 2,3-diastereomer. Crystal structures of all three isomers are included. These findings are relevant to other uses of CDA protections where occurrence of apparently disfavored isomers may be occurring, along with interconversions between CDA isomers.

Glycosaminoglycans (GAGs) are complex linear oligsaccharides involved in multiple biological processes, in both host cell regulation and differentiation, but also in almost all known pathogen-host cell–cell and cell–extracellular matrix interactions. Synthetic GAGs are of value for investigations of such interactions and hold promise as potential therapeutics. Two of the four main types of GAG sequences contain glucuronic acid. Heparin/Heparan sulfate (H/HS), the most structurally heterogeneous GAG, is comprised of alternating glucosamine (GlcN)-derived units and glucuronic (GlcA) or iduronic (IdoA), and Chondroitin sulfate (CS) consists of alternating GlcA and galactosamine (GalN) units. GlcA reagents and mimetics are key synthetic targets relevant to synthetic GAG chemistry, and also have importance for glucuronate conjugates more broadly.^1^

During investigations of routes to uronic acid building blocks for GAG synthesis,^2−4^ we have also been evaluating routes to glucuronates (GlcA),^3,4^ relevant to GlcA-containing CS/HS sequences and mimetics. As part of this work, we evaluated use of cyclohexane-1,2-diacetal (CDA protection), aware of the regioisomeric protections (of bis-equatorial O2,O3 and O3,O4 pairs), noting both anticipated isomers would have utility for GlcA targets, and separable mixtures may allow re-equilibration or hydrolytic recycling on scale.

To our knowledge, the reactivity of CDA formation with uronic acids has not been reported. Here we report that synthesis of CDA protected glucuronic acid derivatives leads to concurrent isolation of an unexpected cis-bis-methoxy CDA derivative, the first example reporting CDA acetal without trans methoxys. Secondly, investigations show that formation and the interconversion between these isomers is more complex than hitherto considered. This may have useful implications for deployment of CDA related acetals.

The use of cyclohexane-1,2-diacetal (CDA) for selective protection of trans-1-2-diols was pioneered by Ley’s group.^5^ This is of particular value in carbohydrates, complimenting methods for selective protections of 1,2-cis diols and 1,3-diols in sugars. Butane-2,3-diacetals (BDA) have similarly been deployed for oligosaccharide synthetic applications through 1,2-bis equatorial diol protections, in particular for mannosyl and galactosyl systems.^6^ Ley’s group employed CDA derivatives for a diverse range of saccharide fragment syntheses.^5,6^ Invariably, conformational and multiple anomeric effects contribute to formation of thermodynamically favored diastereomers with a trans-6,6-ring formation and bis-axial CDA methoxy groups. The Ley conditions for synthesis of carbohydrate CDA acetals employed (+)-camphor-10-sulfonic acid, trimethylorthoformate, and 1,1,2,2-tetramethoxycyclohexane under reflux in MeOH. Examples include protection of glucosides,^5^ galactoside,^5^ mannosyl^5,7^ and rhamnosyl thioglycosides,^5^ and lyxosides.^5^ Protection of 1,2-diols using the parent 1,2-cyclohexanedione was reported for a noncarbohydrate diol;^8^ however, this method was not applied to sugars.^9^

As far as we are aware, diastereomers not adopting the anticipated thermodynamic outcome involving trans-methoxy groups have not been described.

Glucuronic acid thioglycoside **1** was prepared as we reported^4^ from per-acetylated Glc, offering advantages over our prior route.^3,4^ Reaction of glucuronic acid β-thioglycosides **1**(3) with 1,1,2,2-tetramethoxycyclohexane (TMCH) and catalytic CSA under various conditions provided significant amounts of both of the anticipated 1,2-trans-diol protected products 2,3-RR (**2**) and 3,4-SS isomer (**3**), isolated as major products after 24 h (stereolabels refer to the acetal carbons of the protecting group) (Scheme 1). These isomers have optimal combinations of ring and anomeric stabilizing factors.

**Scheme 1:**
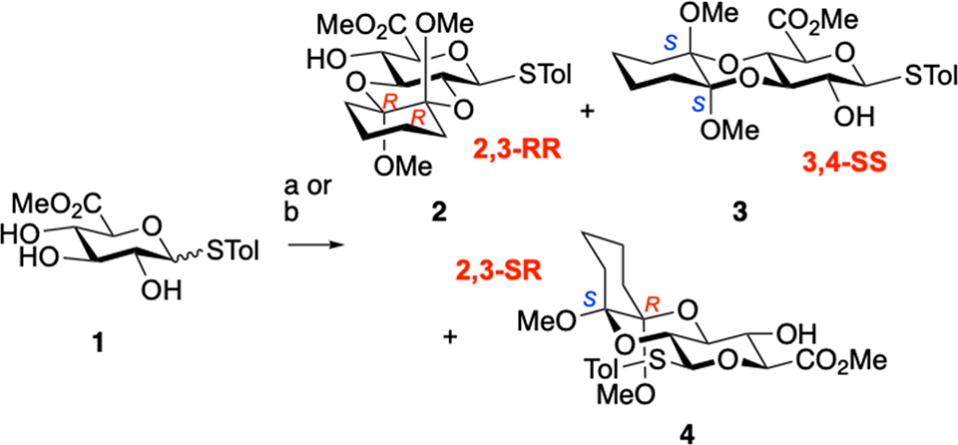
CDA Protection of Glucuronic Acid β-Thioglycoside Reagents and conditions: (a) trimethylorthoformate (1.3–1.4 equiv), (+)-camphorsulfonic acid (catalytic), MeOH, 1,1,2,2-TMCycH (1.4–1.6 equiv), 70 °C, 24 h, **2** (37%), **3** (40%), **4** (9%); (b) cyclohexane-1,2-dione (4.1 equiv), trimethylorthoformate (15-16.1 equiv), (+)-camphorsulfonic acid (catalytic), 70 °C, 24 h, 2 (45%), **3** (46%), **4** (5%). 2% of another isomer obtained; 4% of mixture of unidentified isomers isolated (*vide infra*). Yields for (b) based on product containing the mixture of the three isomers only (see SI).

The ratio of these two major acetals isolated varied as a function of reaction time, though 2,3-RR (**2**) was routinely isolated in slightly higher amounts. We reasoned that using cyclohexane-1-2-dione and methanol (in place of 1,1,2,2-tetramethoxycyclohexane (TMCH)) could lead to direct formation of GlcA CDA acetals *in situ*, without the need to use TMCH. This modification allowed multigram synthesis of the anticipated acetals (**2** and **3**), again in an ∼1:1 ratio.

Under such apparently thermodynamic conditions we had expected, consistent with other reported CDA acetal syntheses, only these trans-methoxy acetal products to form. There are six further possible alternative CDA acetals (Supporting Information (SI), Figure S1), three further diastereomers possible for each of 2,3- and 3,4-CDA outcomes. One in each case is the alternative trans-dimethoxy system (i.e., a double diastereomer) to the two main isolated isomers, **2** and **3** (central ring adopting a boat-like conformation). However, the other possible diastereomers all have cis-methoxy groups, which could adopt all-chair conformers with a cis-decalin arrangement of the CDA associated rings. Any alternative CDA diasteromeric acetals appear not to have been previously reported in other CDA reactions.

We observed that reaction of **1** was accompanied by formation of ≥10% of other product(s). A third isomer was reproducibly isolated as the major alternative product in ∼10% yield. This was shown to be the 2,3-SR isomer **4**. Much smaller amounts (<2%) of two other isomers were isolated and discussed below. The ^1^H NMR spectrum of isomers **2**–**4** have clearly defined differences (SI Figure S2).

Structures of the two anticipated trans-methoxy acetals and of this unexpected 2,3-SR isomer, **4**, were confirmed by crystal structures of 3,4-SS and 2,3-RR products **3** and **4**, and of the 4-OTCA derivative **5** of the 2,3-RR isomer (**2**) (CCDC 2173459 (compound **3**), CCDC 2173458 (compound **4**), and CCDC 2103934 (compound **5**); Figure 1).

**Figure 1:**
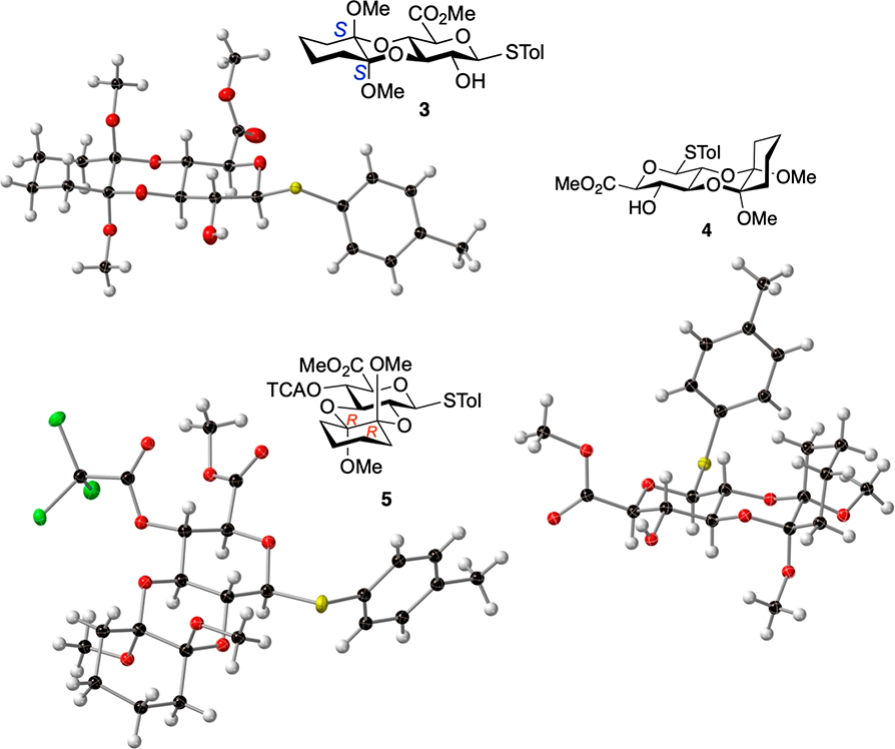
Crystal structures of GlcA-CDA derivatives **3**, **4**, and **5**. **3** crystallized with four independent molecules per unit molecular cell differentiated by the C1–S–CAr–CAr torsion angles [31.67°, 65.55°, 80.53°, and 81.38°]. [ORTEPs at 50% probability; see SI Figures S3–S5.]

With these observations for the presumed thermodynamic end-point (after 24 h), reaction conversion and isomer ratios at shorter reaction times were evaluated. The starting triol is steadily consumed over 8 h. Between 8 h and 24 h there is no starting material and only thus scope for interconversions.

After 2 h the reaction had gone to ∼60% conversion, with almost equal amounts of **2**–**4** (∼20–25%). Over the next 6 h, the amount of the minor cis-methoxy 2,3-SR isomer **4** varied to some extent, and the percentage of the two major isomers steadily increased, consistent with the rate of disappearance of starting material, **1**. From later reaction time points the minor isomer **4** is routinely obtained in about 5–10% yield after 24 h, suggesting rapid initial formation of minor isomer **4**, with slower isomerization over 8–24 h. A decrease in the amount of **4** corresponds approximately to the increase in the amount of the two main isomers (Table 1). Extended (3 d) times lead to consistent quantities of the minor isomer, **4**.

**Table 1:** Monitoring Starting Material **1** Consumption and the Formation of the Corresponding Isomers, 3,4-SS (**3**), 2,3-RR (**2**), and 2,3-SR (**4**)^a^

Time	SM	3,4-SS (3)	2,3-RR (2)	2,3-SR (4)
2 h	36	20	21	23
4 h	21	21	32	26
6 h	17	29	25	19
8 h	0	38	40	22
24 h	0	47	47	6

a *Conditions*: trimethylorthoformate (15–16.1 equiv), (+)-camphorsulfonic acid (catalytic), 70 °C (Percentages were determined by using the ^1^H NMR integrals of the anomeric peaks of the starting material **1** and the three isomers, **2**, **3**, and **4**).

We then considered evidence for isomerization between all three isomers. Reactions were conducted starting with pure samples of **2**–**4**, both with, or without, additional cyclohexadione (and excess TMOF to ensure the products remain as CDA acetals), and product distributions determined at 24 and 72 h. Those run without added dione were thus evaluating direct acid-catalyzed equilibrations without new reagent (Figure 2).

**Figure 2:**
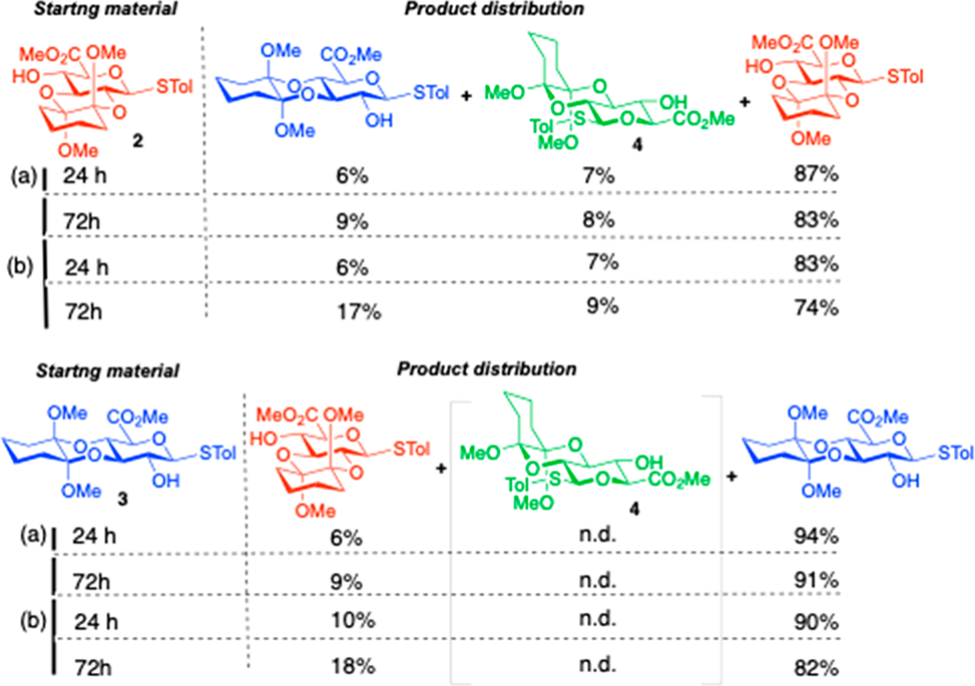
Isomerization reactions of the 2,3-RR and 3,4-SS isomers. Reagents and conditions: (a) trimethylorthoformate (16 equiv), (+)-camphor sulfonic acid (0.5 equiv), dry MeOH, 24 or 72 h, 70 °C; (b) cyclohexane-1,2-dione (4.1 equiv), trimethylorthoformate (16 equiv), (+)-camphor sulfonic acid (0.5 equiv), dry MeOH, 24 or 72 h, 70 °C. Products mixtures isolated with 94–97% mass recovery; ratios determined by NMR (see SI Figure S2).

The outcomes at 24 h for reactions starting from the major isomers (2,3-RR **2** and 3,4-SS **3**; Figure 2) showed a slightly higher degree of isomerization under conditions with added dione; significantly more on the extended 72 h reaction times. However, in all cases ca. 83–94% of the starting materials **3** and **2** remained unchanged.

Intriguingly, 3,4-SS isomer **3** only showed evidence of isomerization to the 2,3-RR **2** isomer with no detectable amounts of minor 2,3-SR diastereomer **4**. In contrast, the 2,3-RR isomer **2** showed isomerization providing similar amounts of both 3,4-SS isomer **3**, and the minor 2,3-SR diastereomer **4**, both without added dione up to 72 h and after 24 h with added dione (Figure 2). This is consistent with isomerization to minor isomer **4** only through its 2,3-diastereomer, and hence the very low/undetected amounts of **4** in these experiments where conversion overall is low (*vide infra.*).

Interconversions were readily quantified using the distinctive anomeric signals in ^1^H NMR (Figure 3). Given the evolution over 72 h we reasoned that these interconversions are slow and may not have reached equilibrium since a near 50:50 ratio of major isomers is obtained starting from the diol, **1**, itself, but very extended reactions lead to increasing accompanying hydrolysis.

**Figure 3:**
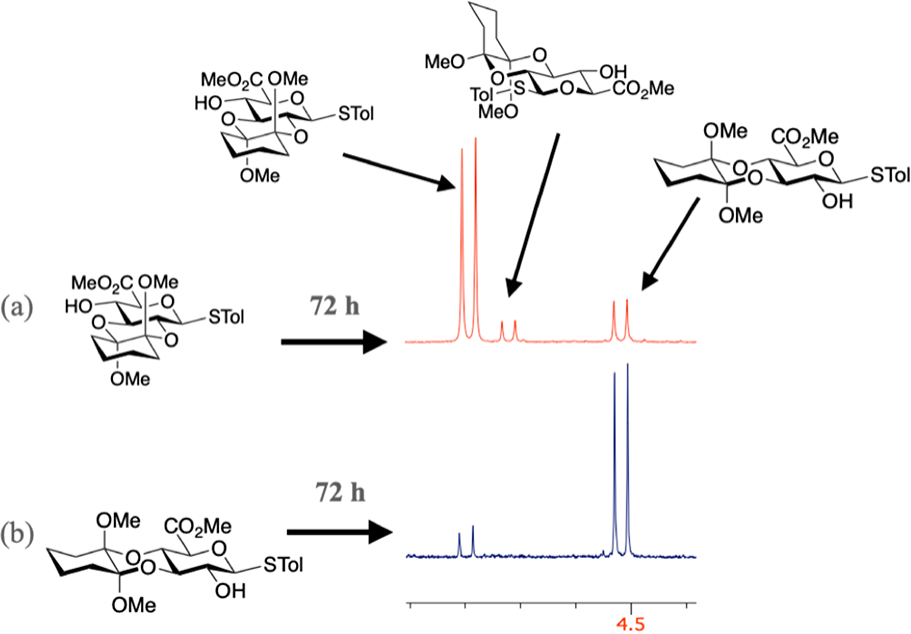
Anomeric peaks of outcomes of isomerizations of 2,3-RR **2** and 3,4-SS isomers **3** over 72 h with no added 1,2-cyclohexadione: (a) Isomerization from **2**; (b) Isomerization from **3**.

The same conditions applied to minor 2,3-SR isomer **4** lead to a much higher degree of isomerization. Most of starting **4** was converted to 2,3-RR isomer **2** (Figure 4) with other minor products accounting for more material than the remaining **4**.

**Figure 4:**
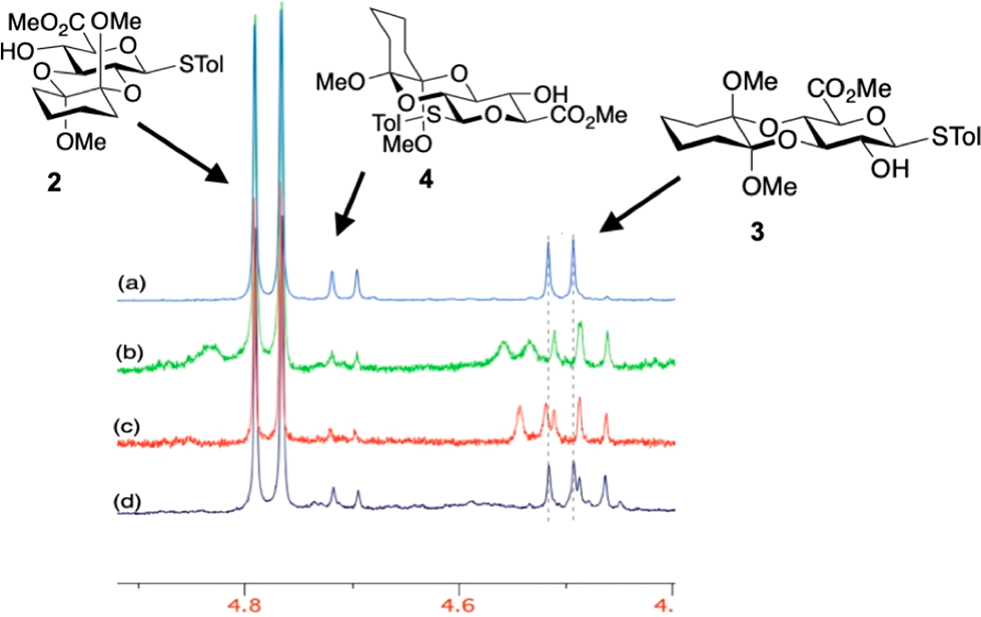
Isomerization starting from isomer **4**. (a) Reference of three main isomers; (b) 72 h without dione; (c) 24 h with dione (no change by NMR at 72 h); and (d) 24 h without dione.

The isomer mixture differs depending on the presence of added 1,3-cyclohexadione and reaction time. Without added dione, little **4** remains after 24 h (Figure 4d) and further decays by 72 h (Figure 4b), but the minor byproducts mixture also changes. When the reaction is conducted with added dione, **4** is almost gone after 24 h (Figure 4c).

To identify other minor isomers, large scale reactions (4 g) from triol starting material **1** run for 24 h, allowing isolation in ≤2% yields of other minor isomers (cf. SI Figure S1). NOESY data of one isomer indicated interaction of axial protons H2 and H4, and between H2 and both cyclohexane methoxy groups, uniquely consistent with the 2,3-RS isomer **6** (Figure 5).

**Figure 5:**
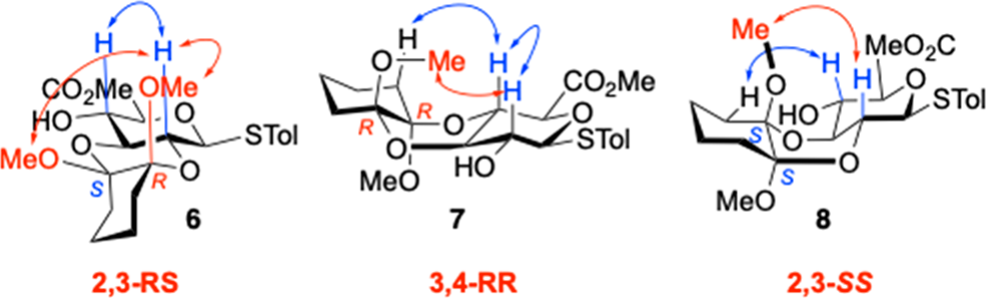
NOESY contacts consistent with further minor isomers. Red contacts from GlcA ring protons to OMe groups of CDA; blue contacts between GlcA ring protons H2 and H4, and/or to CDA cyclohexyl.

A second minor isomer showed interaction between H2 to a single OMe, and H4 to one acetal ring H, consistent with either the 3,4-RR or 2,3-SS isomer **7** and **8**. The H2–H4 interactions are consistent with a ^4^C_1_ GlcA conformation.

The pathways for interconversions between the identified isomers and with the remaining theoretical isomers (SI Figure S1) involve either single acetal carbon epimerizations (without positional migration) or positional isomerization alongside epimerizations. Interconversion pathways between some thus may involve two sequential single-site epimerizations without change of regiochemistry, and or subsequent positional isomerization. Mechanistic options include acetal opening, rotation and recyclization, or full CDA loss and reformation.

For the 3,4-SS isomer, **3**, interconversion with isomer **4** requires (at least) two sequential acetal epimerizations without positional isomerism (two paths to 3,4-RR) followed by opening of the O4 acetal and reclosure to the 2,3-SR isomer, **4**. In contrast, a single acetal carbon epimerization allows for interconversion of the 2,3-RR, **2**, and 2,3-SR isomer **4**. The lack of evidence for interconversion between 3,4-SS isomer **3** and 2,3-SR isomer **4** where two or three steps, including ring opening and closing for migration, are necessary (Figure 6) may argue against a full CDA release pathway being significant.

**Figure 6:**
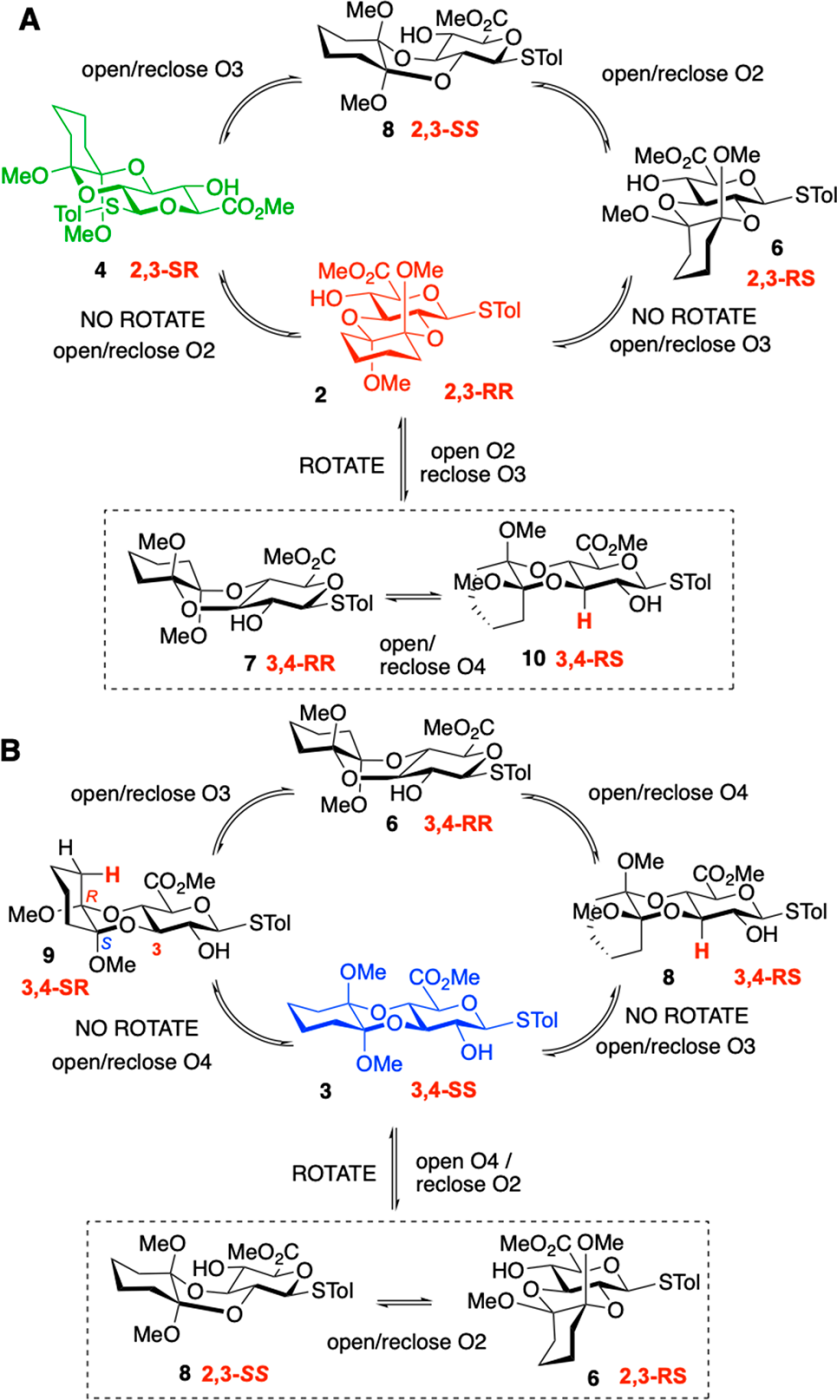
Interconversion pathways between isolated isomers.

In conclusion, CDA acetal formation from a glucuronic acid thioglycoside identified that, along with the expected all-trans decalin O2,O3 and O3,O4 CDA-protected isomers, an unexpected diastereomer of the 2-3-CDA protected GlcA is obtained as a main minor product. Neither observation of such alternative isomers nor evaluation of the rates of formation and interconversions of CDA acetals has previously been reported. Isomerization experiments indicate differential degrees of interconversion between these isomers. This has implications for CDA protections in other systems. The two main CDA protected GlcA thioglycosides reported here, **2** and **3**, can be prepared and readily separated on multigram scale, and are both potentially useful for further elaborations of GlcA derivatives to GlcA-containing saccharide fragments.

## Data Availability

The data underlying this study are available in the published article and its Supporting Information.

## Supporting Information Available

Copies of ^1^H and ^13^C NMR, COSY, and HMQC/HSQC spectra, mass spectra (isotope patterns) for new compounds, and crystal data thermal ellipsoid ORTEPs. (PDF)
