# Supplementary material for: Unexpected Diastereomer Formation and Interconversions in Cyclohexane-1,2-diacetal Derivatization of a Glucuronic Acid Thioglycoside
Source: Org Lett. 2023 Mar 27;25(13):2196–200. doi: 10.1021/acs.orglett.3c00255 (PMC10088025; doi:10.1021/acs.orglett.3c00255)

Supporting information

Unexpected diastereomer formation and interconversions in cyclohexane-1,2-diacetal derivatization of a glucuronic acid thioglycoside

Fahad Ayesh Alharthi,[a] Garrett T. Potter,[a] Gordon C. Jayson,[b] George F. S. Whitehead,[a] Iñigo J. Vitórica-Yrezábal[a] and John M. Gardiner[a]*

[a]Department of Chemistry, School of Natural Sciences, The University of Manchester, Oxford Road, Manchester M13 9PL, U.K.

[b]Institute of Cancer Sciences, Faculty of Medical and Human Sciences, The University of Manchester, Manchester M20 4BX, UK

**Index of Supporting Data**	**pg**
Synthetic procedures and characterisation data	S2-S5
^{1}HNMR, COSY, ^{13}C, HSQC, and NOSEY spectra of the phenyl 2,3-O-(1,2-dimethoxy-1,2-cyclohexanediyl)-4-methyl-1-thio-6-methyl-β-D-glucuronic acid **2**	S6-S9
^{1}H NMR, COSY, ^{13}C, HSQC, and NOSEY spectra of phenyl 3,4-O-(1,2-dimethoxy-1,2-cyclohexanediyl)-4-methyl-1-thio-6-methyl-β-D-glucuronic acid **3**	S9-12
^{1}HNMR, COSY, ^{13}C, HSQC, and NOSEY spectra of the GlcA-CDA odd isomer **4**	S13-S16
^{1}HNMR, COSY, ^{13}C, HSQC, and NOSEY spectra of the phenyl 2,3-O-(1,2-dimethoxy-1,2-cyclohexanediyl)-4-trichloroacetyl-4-methyl-1-thio-6-methyl-β- D-glucuronic acid **5**	S16-S19
Experimental data text for **6**. ^{1}H NMR, COSY, ^{13}C, HSQC, and NOSEY spectra 2,3-RS GlcA-CDA isomer **6**.	S19-S23
^{1}H NMR, COSY, ^{13}C, HSQC, and NOSEY spectra 2,3-SS GlcA-CDA isomer **8** (or **7**)	S24-S28
^{1}H NMR spectra showing specific peak integrations of mixtures	S39-S35
Fig S1: Alternative isomers	S35
Fig S2: Spectra comparing anomeric all three main isomers	S36
^{1}H NMR, COSY, ^{13}C, HSQC of starting material **1**	S37-38
Fig S3: ORTEP at 50% probability for O4-TCA 2,3-CDA acetal, **5**.	S39
Fig S4: ORTEP at 50% probability for 3,4-CDA acetal, **3**.	S40
Fig S5A/B: ORTEP at 50% probability for S,R 3,4-CDA acetal, **4**.	S41-2

Experimental Section

NMR spectra were referenced to the residual solvent signal. ^{13}C shifts are given to 1 d.p. unless 2 d.p. is required to distinguish peaks. Coupling constants (J values) are quoted to the nearest 0.1 Hz and are given as observed i.e. not made equal for coupling protons. Preparative column chromatography was performed using Sigma-Aldrich silica gel (technical grade, 60 Å, 220–240 mesh, 35–75 μm) and the flash technique.1 All solvents used were of standard laboratory grade unless otherwise specified. 'Pet. ether' refers to the petroleum ether fraction with boiling point 40–60 °C. Compositions of solvent mixtures are quoted as ratios of volumes unless otherwise stated. Organic solutions were dried with anhydrous $MgSO_4$ and concentrated by rotary evaporation under reduced pressure. Reactions where heated used DrySyn® heating blocks. Structural assignments were made with additional information from gCOSY, gHSQC, and gHMBC experiments
XRD data were collected using a Rigaku FR-X DW diffractometer, equipped with an FR-X high intensity rotating anode Cu Kα radiation source (λ=1.54184 Å) and VariMAXTM microfocus optics, using an AFC-11 4-circle goniometer, a HyPix-6000HE hybrid pixel array detector operating in shutterless mode and an Oxford Cryosystems Cryostream 800 plus nitrogen flow gas system at a temperature of 100K. Data were collected, integrated and reduced using Rigaku Oxford Diffraction CryAlisPro v171.42.[11] Structures were solved and refined using ShelX-20XX implemented through Olex2.[12,13]

Preparation of phenyl 2,3-*O*-(1,2-dimethoxy-1,2-cyclohexanediyl)-4-methyl-1-thio-6-methyl-β-D-glucuronic acid

Method A: To triol **1**[4] (0.7763 g, 2.47 mmol) and (+)-camphor-10-sulfonic acid (0.1056 g, 0.34 mmol) in a 50 mL round bottom flask under N_2 atmosphere was added anhydrous MeOH (50 mL), trimethylorthoformate (0.38 mL, 3.47 mmol), and 1,1,2,2-tetramethoxycyclohexane (0.76 mL, 3.96 mmol). The solution was then heated under reflux at 70 °C for 24 hours, whereupon the reaction was judged complete by the consumption of the starting material 1 on TLC. The reaction solvent was then removed in vacuo, and the resulting crude product was further purified using column chromatography (gradient: 100% n-hexane, followed by 7:1 DCM/ EtOAc, followed by 40% EtOAc in n-hexane) which yielded four products: 2,3-CDA acetal **2**, as a colourless foam (0.4110 g, 37 %, 0.91 mmol), 3,4-CDA acetal **3** as a colourless gel-like liquid that could be recrystallized by slow evaporation from DCM (0.4548 g, 40 %, 1.00 mmol), and 2,3-CDA acetal **4** as a colourless gel-like liquid that could be recrystallized by slow evaporation from $CDCl_3$ (0.1045 g, 9 %, 0.23 mmol), along with unreacted starting material **1** (0.0950 g, 0.30 mmol, 12%). A mixture of other side-products was also isolated but could not be further separated by column chromatography on this scale (0.0237g, 0.05 mmol, total ~2%). Reaction on 4 g scale and a further column chromatography using 1:1 EtOAc/DCM provided 5-10 mg of two minor by-products, each as a colourless liquid, whose structures have been tentatively proposed.

Method B: To the deprotected thioglycoside **1**[4] (0.3631 g, 1.15 mmol) was added 1,2-cyclohexanedione (0.5210 g, 4.64 mmol). While kept under a flow of N_2 gas, dry MeOH (19 mL), CAS (0.1440 g, 0.61 mmol), trimethylorthoformate (2.0 mL, 18.3 mmol) were added. The reaction vessel was fitted with a condenser, and the reaction solution was heated to reflux at 70 °C and under N_2 atmosphere for 8 hours. The solvent was evaporated in *vacuo* to give a crude product as mixture of isomers (0.5081g, 98%, 1.1 mmol), consisting of isomers **2** (40 %), **3** (38 %), and **4** (22 %). Percentages determined using integration of anomeric peaks.

2,3-CDA Acetal 2: ^1H NMR ($CDCl_3$, 400 MHz) δ 7.45 (d, *J* = 8.1 Hz, 2 H, Ar-H), 7.09 (d, *J* = 8.0 Hz, 2 H, Ar-H), 4.77 (d, *J* = 9.8 Hz, 1 H, H-1), 4.00 (t, *J* = 9.3 Hz, 1 H, H-4), 3.94-3.87 (m, 2 H, H-3 & H-5), 3.83 (s, 3 H, $-COOCH_3$), 3.78 (d, *J* = 9.8 Hz, 1 H, H-2), 3.24 (s, 3 H, $-COCH_3$), 3.13 (s, 3 H, $-COCH_3$), 2.32 (s, 3 H, Ar-CH_3), 1.88-1.80 (m, 2 H, $-CH_2-$), 1.79-1.69 (m, 2 H, $-CH_2-$), 1.57-1.50 (m, 2 H, $-CH_2-$), 1.43-1.33 (m, 2 H,$-CH_2-$).

^{13}C NMR ($CDCl_3$, 100 MHz) δ 169.4 (C6), 138.0 (Ar-CH_3), 132.8 (2 x Ar-H), 129.6 (2 x Ar-H), 128.9 (Ar), 99.0 (-$COCH_3$), 98.6 (-$COCH_3$), 86.4 (C1), 78.3 (C5), 73.9 (C3), 68.9 (C4), 68.1 (C2), 52.8 (-$COOCH_3$), 47.0 (-$COCH_3$), 46.9 (-$COCH_3$), 27.0 (-CH_2-), 26.9 (-CH_2-), 21.4 (-CH_2-), 21.3 (-CH_2-), 21.1 (Ar-CH_3). FTIR *v* max/cm^{-1} 3450 (br, O-H stretch), 1749 (C=O stretch), 1183 (acyl C-O stretch), 1053 (alkoxy C-O stretch) cm^{-1}; Product R_f = 0.07, 40% EtOAc in n-hexane. ES MS *m/z* 477 [M+Na]$^+$, 100%); HRMS (Orbitrap-H-ESI) *m/z* [M+Na]$^+$ Calcd for $C_{22}H_{30}O_8SNa$ 477.1559; Found 477.1554, Melting Point = 70 – 73 °C. $[\alpha]_D$ = –108.4 (CH_2Cl_2). All data values are novel and have not yet been reported.

Eg HRMS (ESI-TOF) m/z: [M + Na]+ Calcd for C13H17NO3Na 258.1101; Found 258.1074.

3,4-CDA Acetal 3: ^{1}H NMR ($CDCl_3$, 400 MHz) 7.41 (d, *J* = 8.0 Hz, 2 H, Ar-H), 7.09 (d, *J* = 7.9 Hz, 2 H, Ar-H), 4.48 (d, *J* = 9.3 Hz, 1 H, H-1), 4.04 (d, *J* = 9.8 Hz, 1H, H-5), 3.96 (t, *J* = 9.9 Hz, 1 H, H-4), 3.88 (t, *J* = 9.4 Hz, 1 H, H-3), 3.76 (s, 3 H, -$COOCH_3$), 3.50 (t, *J* = 9.3 Hz, 1 H, H-2), 3.21 (s, 3 H, -OCH_3), 3.14 (s, 3 H,-OCH_3), 2.65 (s, br, 1 H, -OH), 2.31 (s, 3 H, Ar-CH_3),1.82-1.64 (m, 4 H, -CH_2-), 1.53-1.41 (m, 2 H, -CH_2-), 1.41-1.27 (m, 2 H, -CH_2-).
^{13}C-NMR ($CDCl_3$, 100 MHz) δ 167.8 (C6), 138.8 (Ar-CH_3), 133.9 (2 x Ar- H), 129.9 (2 x Ar- H), 126.9 (Ar), 98.8 (-$COCH_3$), 98.6 (-$COCH_3$), 89.4 (C1), 76.9 (C5), 73.3 (C3), 68.8 (C2), 67.8 (C4), 52.4 (-$COOCH_3$), 46.9 (2 x -$COCH_3$), 27.0 (-CH_2-), 26.8 (-CH_2-), 21.3 (2 x -CH_2-), 21.2 (Ar-CH_3). FTIR v_{max}/cm^{-1} 3450 (br, O-H stretch), 1748 (C=O stretch), 1183 (acyl C-O stretch) 1052 (alkoxy C-O stretch) cm^{-1}; Product R_f = 0.21, 40% EtOAc in n-hexane; ES MS *m/z* 477 (MNa^+, 100%); HRMS (Orbitrap-H-ESI) *m/z* [M+Na]$^+$ Calcd for $C_{22}H_{30}O_8SNa$ 477.1559; Found 477.1540, Melting Point = 94 – 98 °C; $[\alpha]_D$ = + 54.9 (CH_2Cl_2). All data values are novel and have not yet been reported.

2,3-CDA Acetal diastereomer 4: ^{1}H NMR ($CDCl_3$, 400 MHz) δ 7.44 (d, *J* = 8.1, 2 H, Ar-H), 7.10 (d, *J* = 8.1 Hz, 2 H, Ar-H), 4.69 (d, *J* = 9.3 Hz, 1 H, H-1), 3.90-3.84 (m, 3 H, H 4, H 3, & H-5), 3.83 (s, 3 H, -$COOCH_3$), 3.41 (s, 3 H, -$COCH_3$), 3.39-3.34 (m, 1 H, H-2), 3.27 (s, 3 H, -$COCH_3$), 2.33 (s, 3 H, Ar-CH_3), 1.90-1.77 (m, 1 H, -CH_2-), 1.68-1.49 (m, 3 H, -CH_2-), 1.43-1.28 (m, 2 H, -CH_2-), 1.43-1.33 (m, 2 H,-CH_2-).
^{13}C NMR ($CDCl_3$, 100 MHz) δ 169.7 (C6), 138.6 (Ar-CH_3), 134.6 (2 x Ar-H), 129.5 (2 x Ar-H), 127.0 (Ar), 99.6 (-$COCH_3$), 98.1 (-$COCH_3$), 85.5 (C1), 78.4 (C5), 73.4 (C3), 70.0 (C4), 68.9 (C2), 52.6 (-$COOCH_3$), 49.0 (-$COCH_3$), 47.5 (-$COCH_3$), 30.6 (-CH_2-), 25.4 (-CH_2-), 21.9 (-CH_2-), 21.8 (-CH_2-), 21.3 (Ar-CH_3); FTIR v_{max}/cm^{-1} 3450 (br, O-H stretch), 1746 (C=O stretch), 1181 (acyl C-O stretch) 1073 (alkoxy C-O stretch); ES MS *m/z* 477 (MNa^+, 100%); HRMS (Orbitrap-H-ESI) *m/z* [M+Na]$^+$ Calcd for $C_{22}H_{30}O_8SNa$ 477.1559,; Found: 477.1554; Product R_f = 0.19 (1:1 n-hexane/EtOAc); $[\alpha]_D$ = - 94.6 (CH_2Cl_2); Melting Point = 64 – 67 °C. All data values are novel and have not yet been reported.

Phenyl 2,3-O-(1,2-dimethoxy-1,2 cyclohexanediyl)-4-trichloroacetyl-4-methyl-1-thio-6-methyl-β-D-glucuronic acid, 5

2 (0.4014 g, 0.88 mmol) in a 100 mL round bottom flask under N_2 atmosphere was added anhydrous DCM (36 mL), pyridine (0.26 mL, 3.23 mmol), and trichloroacetyl chloride (0.1 mL, 0.89 mmol). The reaction was stirred at RT under N_2 overnight after which the reaction was judged complete by consumption of **2** on TLC. DCM (100 mL) was then added and the crude mixture was transferred to a beaker containing HCl (50 mL, 1 M) and sat. aq. $NaHCO_3$ (50 mL). The mixture was vigorously stirred for 5 minutes and the organic sseparated, dried ($MgSO_4$,), filtered, and concentrated to afford the product **5** as white crystals (0.3808g, 72%, 0.63 mmol).

^{1}H NMR (400 MHz; $CDCl_3$): δ 7.47 (d, *J* = 6.5 Hz, 2 H, Ar-H), 7.11 (d, *J* = 6.3, 2 H, Ar), 5.31 (t, *J* = 7.8 Hz, 1 H, H4), 4.78 (d, *J* = 8.0 Hz, 1 H, H-1), 4.18 (t, *J* = 8.0 Hz, 1 H, H-3), 4.11 (d, *J* = 7.7 Hz, 1 H, H-5), 3.92 (t, *J* = 8.0 Hz, 1 H, H-2), 3.76 (s, 3 H, -COOMe), 3.21 (s, 3 H, -OMe), 3.12 (s, 3 H, -OMe), 2.33 (s, 3 H, Ar-CH_3),1.88-1.70 (m, 4 H, -CH_2-), 1.44-1.30 (m, 2 H, -CH_2-), 1.29-1.22 (m, 2 H, -CH_2-).

^{13}C NMR (100 MHz; $CDCl_3$): δ [(166.8, 160.4) C=O], 138.2 (Ar-CH_3),133.1 (2 x Ar-H), 129.7 (2 x Ar-H), 127.3 (Ar), 99.2 (-$COCH_3$), 98.8 (-$COCH_3$), 85.3 (C1), 75.9 (C5), 72.8 (C4), 72.0 (CCl_3), 71.6 (C3), 68.0 (C2), 53.0 (-COOMe), 47.0 (-OCH_3), 46.8 (-OCH_3), 27.0 (-CH_2-), 26.8 (-CH_2-), 21.3 (Ar-CH_3), 21.2 (-CH_2-), 21.2 (-CH_2-); Product R_f = 0.55 (1:1 pet ether/EtOAc); FTIR *v* max/cm^{-1} 2952

(sp^3 C-H stretch), [1764 & 1753 (C=O stretch)], 1430 (sp^3 C-H bend), 1190 (acyl C-O stretch), [1090, 1067, 993 (alkoxy C-O stretch)],; ES MS *m/z* 621 (MNa^+, 100%); HRMS *m/z* $[M+Na]^+$ Calcd for $C_{24}H_{29}Cl_3O_9SNa$ 621.0496; Found: 621.0484; Melting Point = 141 – 145 °C; $[\alpha]_D = -91.8$ (CH_2Cl_2).

Interconversion Reaction: From 3,4-SS 3 (in the presence of 1,2-cyclohexanedionedione.

To O3 and O4 cyclohexane-1,2-diacetal protected thioglycoside **3** (0.1352 g, 0.30 mmol), and 1,2-cyclohexanedione (0.1386 g, 1.22 mmol) dissolved in dry MeOH (10 mL) under N_2 atmosphere were added (+)-10-camphorsulfonic acid (0.0369 g, 0.16 mmol) and trimethyl orthoformate (0.52 mL, 4.8 mmol). The reaction solution was heated to reflux at 70 °C for 72 hours. Removal of the solvent in *vacuo* and washing the resulting brownish solid with hot n-hexane gave a clean mixture of two isomers (0.1270 g, 0.28 mmol, 94%). ^{1}H NMR indicated 82 % of starting material **3**, and 18 % of **2** (Percentages determined using integration of anomeric peaks).

Interconversion Reaction: From 3,4-SS 3 (Absence of 1,2-cyclohexanedionedione).

The O3 and O4 cyclohexane-1,2-diacetal protected thioglycoside **3** (0.0807 g, 0.18 mmol) was dissolved in dry MeOH (7 mL) under an inert atmosphere, then 10-camphorsulfonic acid (0.0230 g, 0.10 mmol) and trimethylorthoformate (0.29 mL, 2.7 mmol) were added. The reaction solution was heated under reflux at 70 °C for 72 hours. The solvent was removed in vacuo to afford a mixture of two isomers (0.0782 g, 0.17 mmol, 97%). ^{1}H NMR indicated 91 % of starting material **3**, and 9 % of **2** (Percentages determined using integration of anomeric peaks).

Interconversion Reaction: From 2,3-*O*-CDA 2 (with 4 eq. 1,2-cyclohexanedione)

The O2 and O3 cyclohexane-1,2-diacetal protected thioglycoside **2** (0.2328 g, 0.51 mmol) and 1,2-cyclohexanedione (0.2398 g, 2.11 mmol) were placed in a 50 mL round bottom flask, under N_2 atmosphere, and dissolved in dry MeOH (10 mL). To the reaction solution was added CSA (0.06 g, 0.26 mmol) followed by trimethylorthoformate (0.90 mL, 8.30 mmol). The reaction mixture was heated under reflux at 70 °C and under N_2 atmosphere for 72 hours. The reaction solvent was removed in vacuo, and the resulting brown solid was washed with hot n-hexane to afford a colourless oil as a mixture of isomers (0.2211 g, 048 mmol, 95%). ^{1}H NMR indicated isomer **3** (17%) isomer **4** (9%), and staring material **2** (74%). (Percentages were determined using integration of anomeric peaks of the desired isomers).

Interconversion Reaction: From 2,3-*O*-CDA 2 (Absence of 1,2-cyclohexanedione)

In a 25 mL round bottom flask, the CDA isomer **2** (0.0206 g, 0.04 mmol) was dissolved in of dry MeOH (5 mL). CSA (0.0050 g, 0.0210 mmol) and trimethylorthoformate (0.0750 mL, 0.70 mmol) were added. The reaction mixture was heated under reflux at 70 °C and under N_2 atmosphere for 72 hours. Removal of solvent under reduced pressure yielded the crude product as a mixture of isomers (0.0196 g, 0.04 mmol, 95%). The reaction solvent was removed in vacuo, and the resulting brown solid was washed with hot n-hexane to afford a colourless oil as a mixture of isomers (0.2211 g, 0.48 mmol, 95%). ^{1}H NMR indicated isomer **3** (9%), isomer **4** (8% yield), and staring material **2** (83%). (Percentages were determined using integration of anomeric peaks).

Interconversion Reaction: From 2,3-SR 4 (with 4 eq. 1,2-Cyclohexanedionedione.)

The 2,3-SR acetal **4** (0.0130 g, 0.03 mmol), 1,2-cyclohexanedione (0.0140 g, 0.12 mmol) and 10-camphorsulfonic acid (0.0030 g, 0.013 mmol) were dissolved in dry MeOH (3 mL) under a N_2 atmosphere. Trimethyl orthoformate (0.051 mL, 0.47 mmol) was added and the reaction solution was heated under reflux at 70 °C for 72 hours. The solvent was removed in *vacuo*, and crude was washed with hot n-hexane. This afforded a crude product containing a mixture of two isomers (0.0128 g, 0.11 mmol, 98%). ^{1}H NMR indicated starting material **4** (14 %) and **2** (86%) (Percentages determined using integration of anomeric peaks).

Interconversion Reaction: From 2,3-SR 4 (absence of 1,2-Cyclohexanedionedione.)

In a 10 mL round bottom flask, the 2,3-SR CDA acetal **4** (0.0126 g, 0.03 mmol) and 10-camphorsulfonic acid (0.0030 g, 0.013 mmol) were dissolved in dry MeOH (5 mL) under N_2 atmosphere. Trimethylorthoformate (0.05 mL, 0.46 mmol) was added to the reaction solution. The

reaction solution was heated to reflux at 70 °C for 72 hours. The solvent was removed under reduced pressure, to give a clean product as mixture of isomers (0.0120 g, 0.03 mmol, 95%). ^{1}H NMR revealed that the crude product contained the starting material **4** (15 %) and **2** (85%). (Percentages were determined using integration of anomeric peaks).

XRD data

Crystal Data and structure refinement data for **3** $C_{22}H_{30}O_8S$ (M =454.52 g/mol): monoclinic, space group $P2_1$ (no. 4), a = 14.21250(10) Å, b = 22.1143(2) Å, c = 14.21710(10) Å, β = 93.2260(10)°, V = 4461.35(6) Å^3, Z = 8, T = 100.01(10) K, μ(Cu Kα) = 1.685 mm^{-1}, $Dcalc$ = 1.353 g/cm^3, 66389 reflections measured (3.996° ≤ 2Θ ≤ 152.652°), 17738 unique (R_{int} = 0.0362, R_{sigma} = 0.0273) which were used in all calculations. The final R_1 was 0.0289 (I > 2σ(I)) and wR_2 was 0.0791 (all data). CCDC

Crystal Data and structure refinement data for **4** $C_{23}H_{31}Cl_3O_8S$ (M =573.89 g/mol): orthorhombic, space group $P2_12_12_1$ (no. 19), a = 10.7347(7) Å, b = 11.0420(6) Å, c = 22.9093(15) Å, V = 2715.5(3) Å^3, Z = 4, T = 100.01(10) K, μ(Cu Kα) = 4.155 mm^{-1}, $Dcalc$ = 1.404 g/cm^3, 17556 reflections measured (7.718° ≤ 2Θ ≤ 154.054°), 5536 unique (R_{int} = 0.1109, R_{sigma} = 0.1285) which were used in all calculations. The final R_1 was 0.0714 (I > 2σ(I)) and wR_2 was 0.1982 (all data). CCDC

Crystal Data and structure refinement data for **5** $C_{24}H_{29}Cl_3O_9S$ (M =599.88 g/mol): orthorhombic, space group $P2_12_12$ (no. 18), a = 16.3459(3) Å, b = 28.9686(5) Å, c = 5.68400(10) Å, V = 2691.48(8) Å^3, Z = 4, T = 99.98(11) K, μ(CuKα) = 4.251 mm^{-1}, $Dcalc$ = 1.480 g/cm^3, 14857 reflections measured (6.102° ≤ 2Θ ≤ 152.078°), 5425 unique (R_{int} = 0.0289, R_{sigma} = 0.0342) which were used in all calculations. The final R_1 was 0.0276 (I > 2σ(I)) and wR_2 was 0.0636 (all data).

Spectral Data

^{1}H NMR (400 MHz; $CDCl_3$) spectrum of the phenyl 2,3-O-(1,2-dimethoxy-1,2-cyclohexanediyl)-4-methyl-1-thio-6-methyl-β-D-glucuronic acid 2

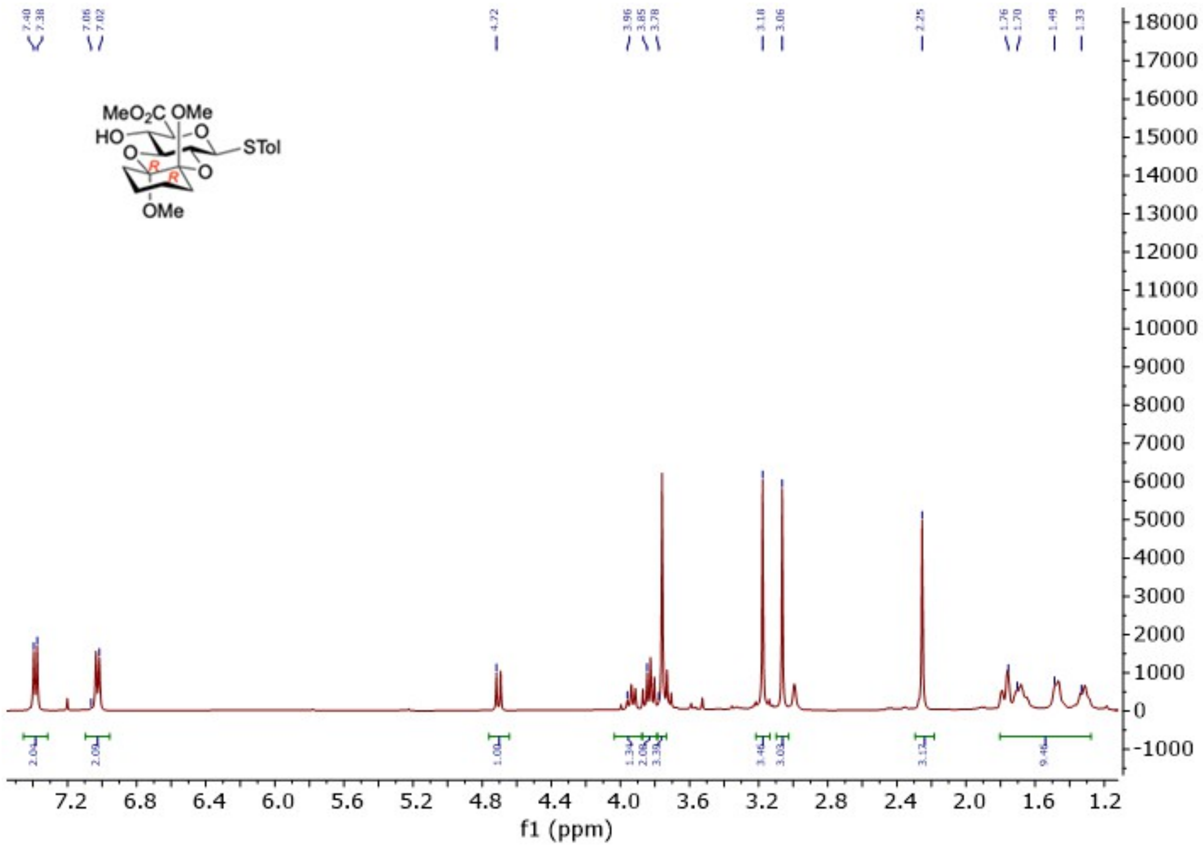

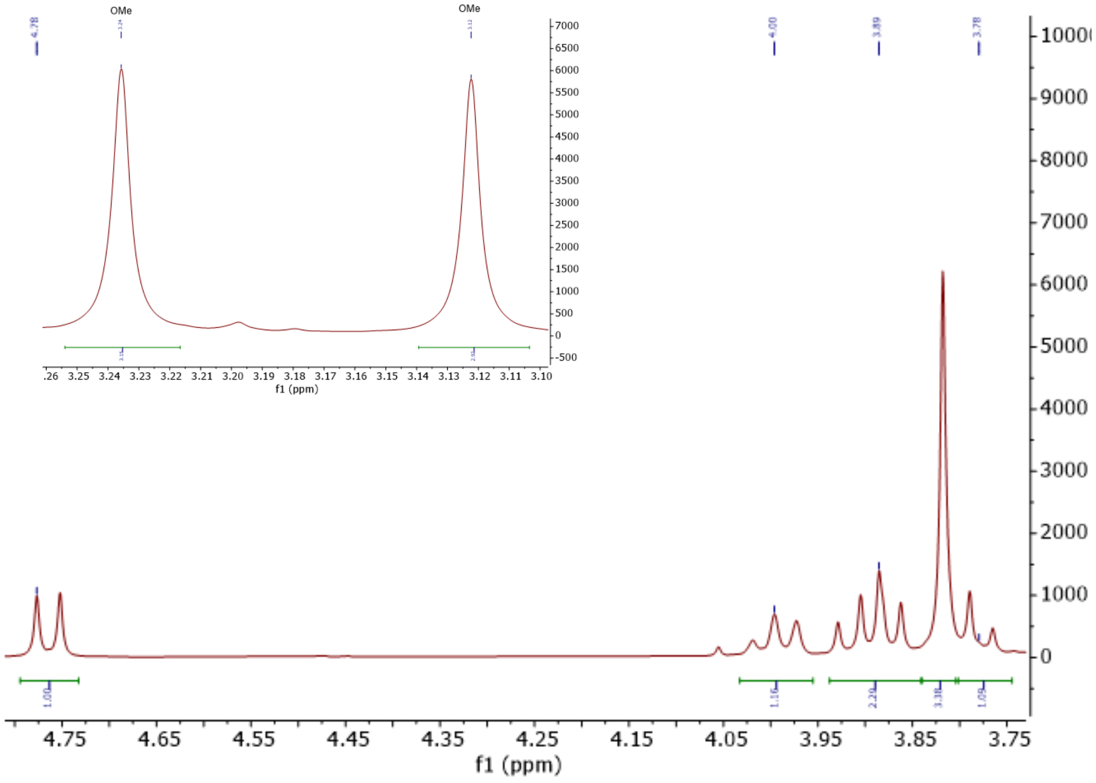

COSY NMR (400 MHz; $CDCl_3$) Spectrum of the phenyl 2,3-O-(1,2-dimethoxy-1,2-cyclohexanediyl)-4-methyl-1-thio-6-methyl-β-D-glucuronic acid 2

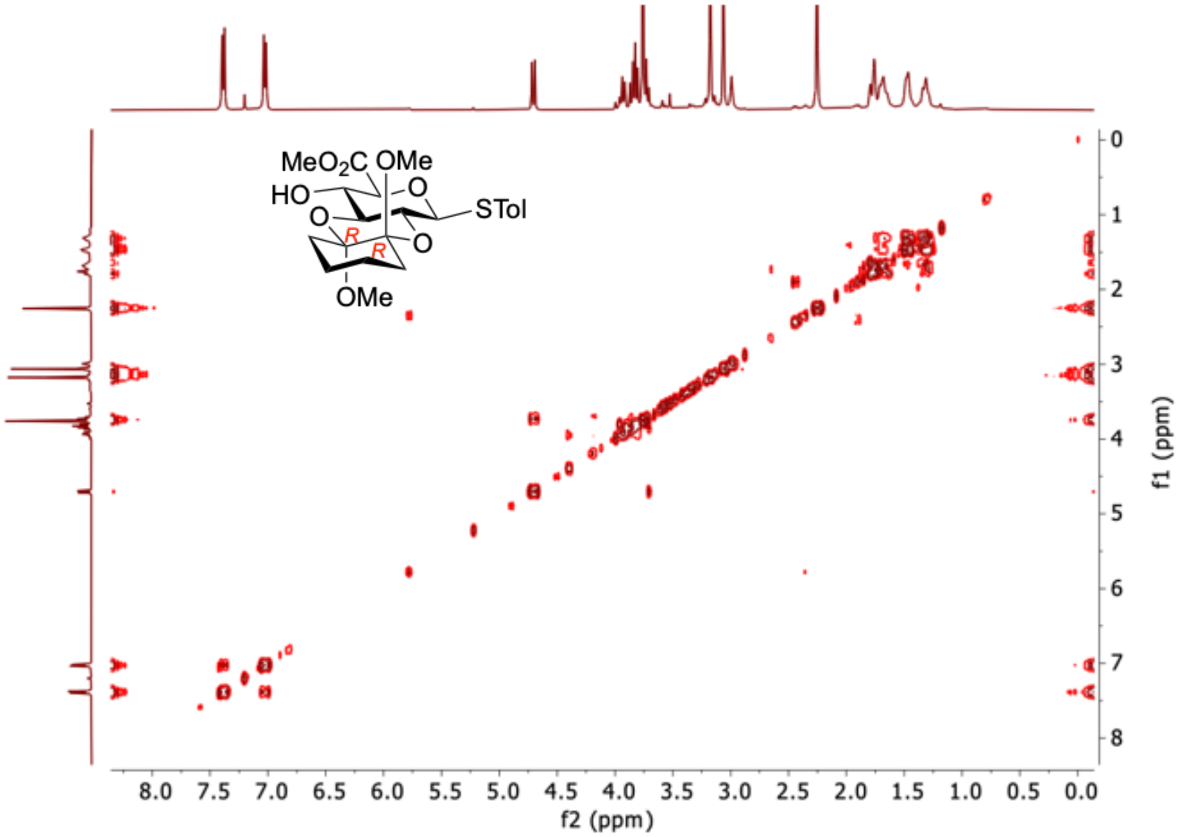

Expansion of COSY NMR spectrum of the phenyl 2,3-O-(1,2-dimethoxy-1,2-cyclohexanediyl)-4-methyl-1-thio-6-methyl-β-D-glucuronic acid 2

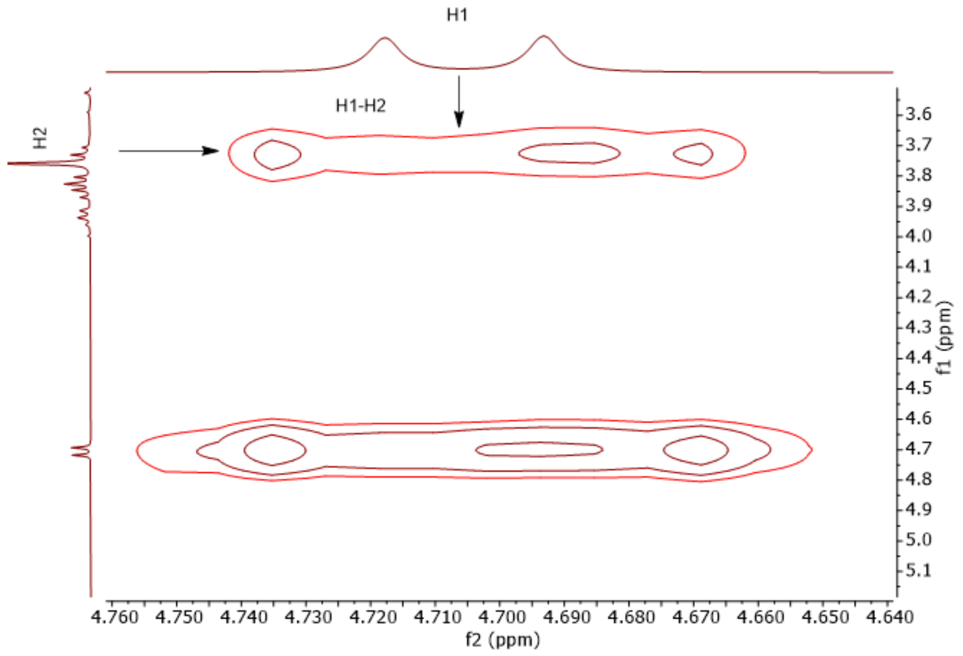

^{13}C NMR (100 MHz; $CDCl_3$) spectrum of the phenyl 2,3-O-(1,2-dimethoxy-1,2-cyclohexanediyl)-4-methyl-1-thio-6-methyl-β-D-glucuronic acid 2

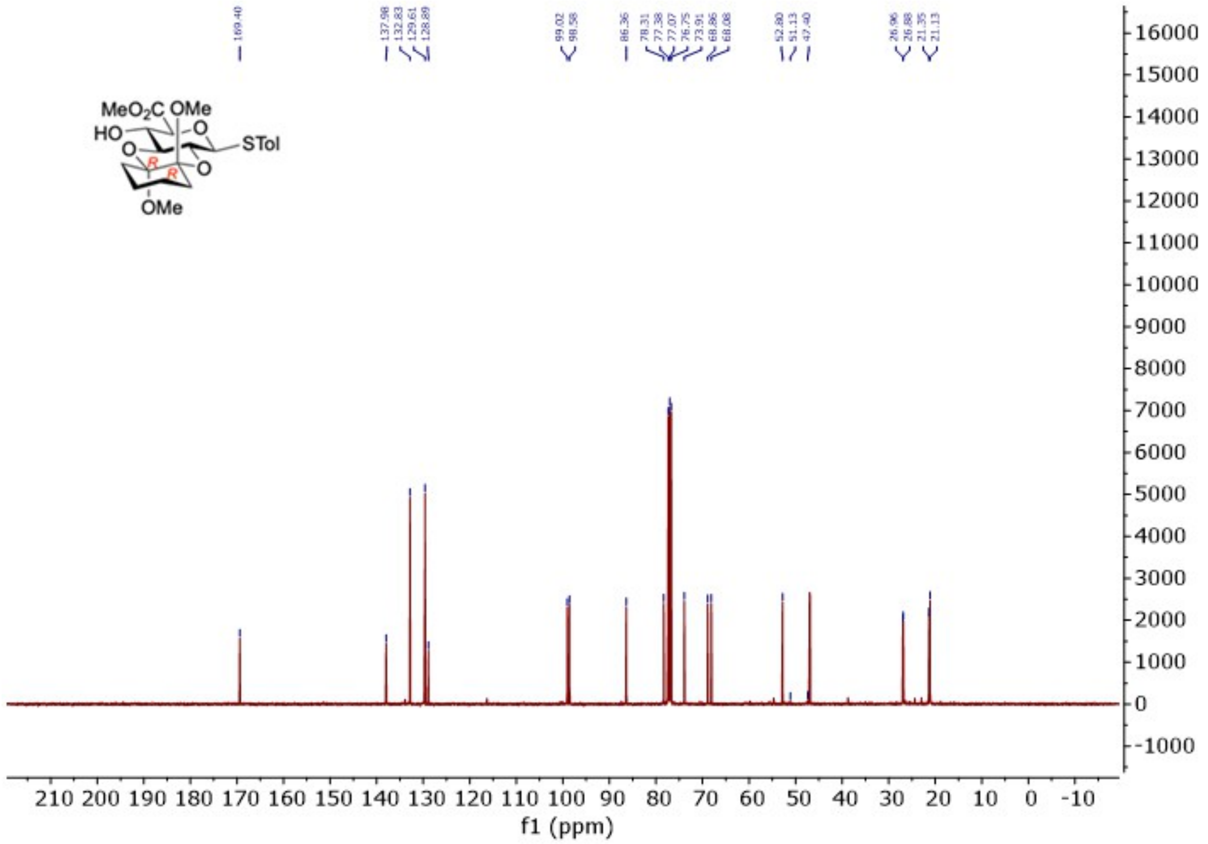

HSQC NMR (400 MHz; $CDCl_3$) spectrum of the phenyl 2,3-O-(1,2-dimethoxy-1,2-cyclohexanediyl)-4-methyl-1-thio-6-methyl-β-D-glucuronic acid 2

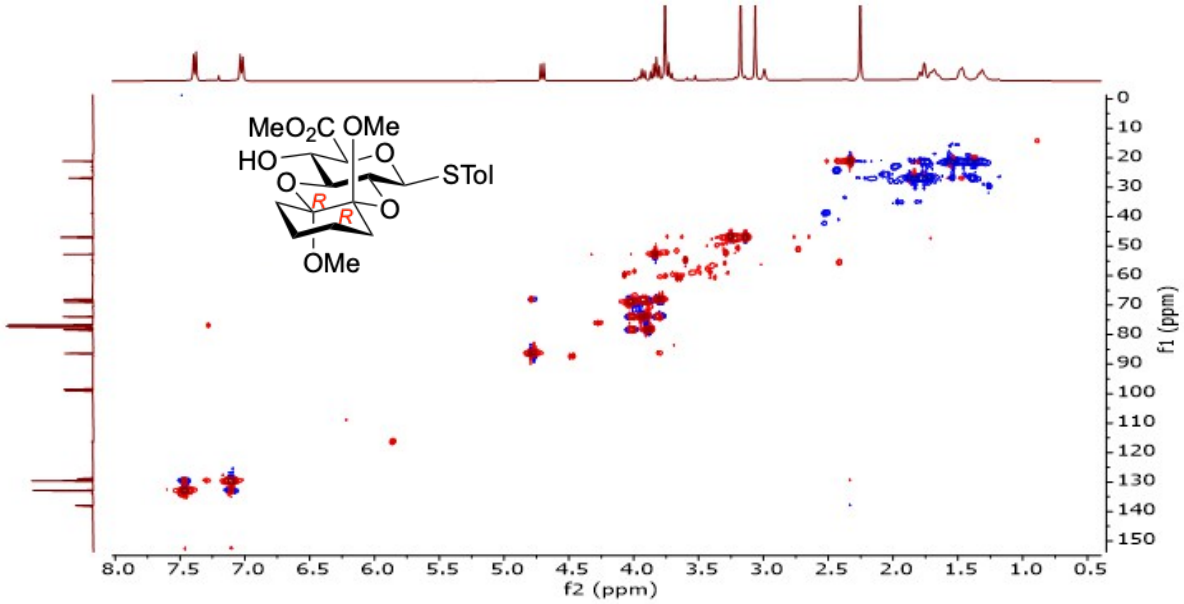

NOESY NMR (400 MHz; CDCl$_3$) spectrum of the phenyl 2,3-*O*-(1,2-dimethoxy-1,2-cyclohexanediyl)-4-methyl-1-thio-6-methyl-β-D-glucuronic acid 2

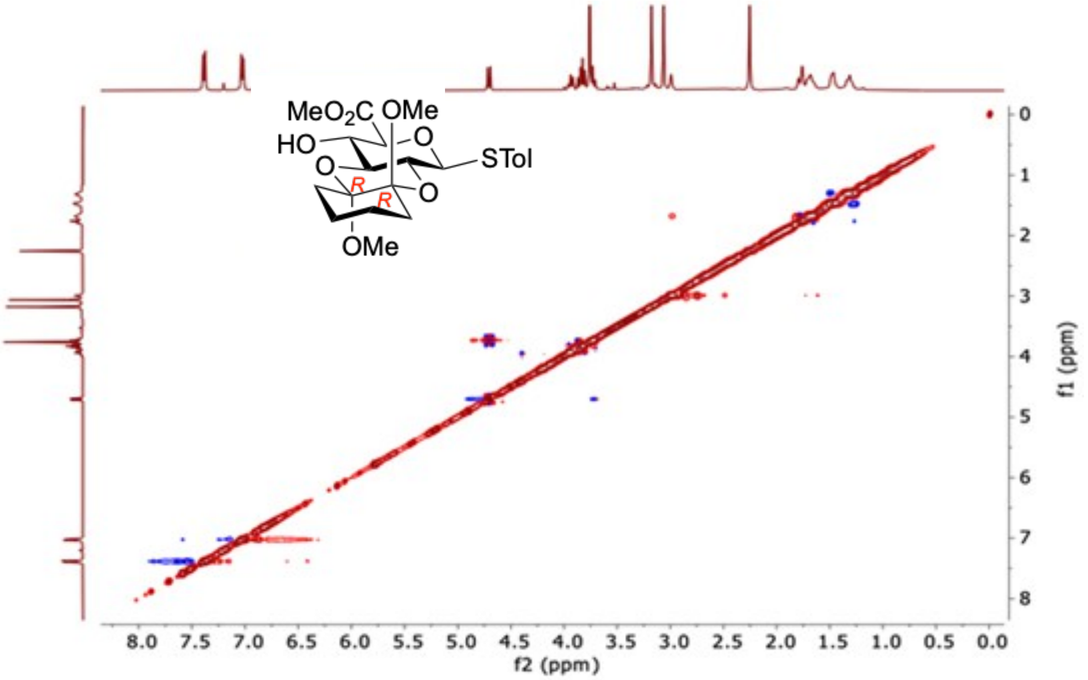

^{1}H NMR (400 MHz; CDCl$_3$) of phenyl 3,4-*O*-(1,2-dimethoxy-1,2-cyclohexanediyl)-4-methyl-1-thio-6-methyl-β-D-glucuronic acid 3

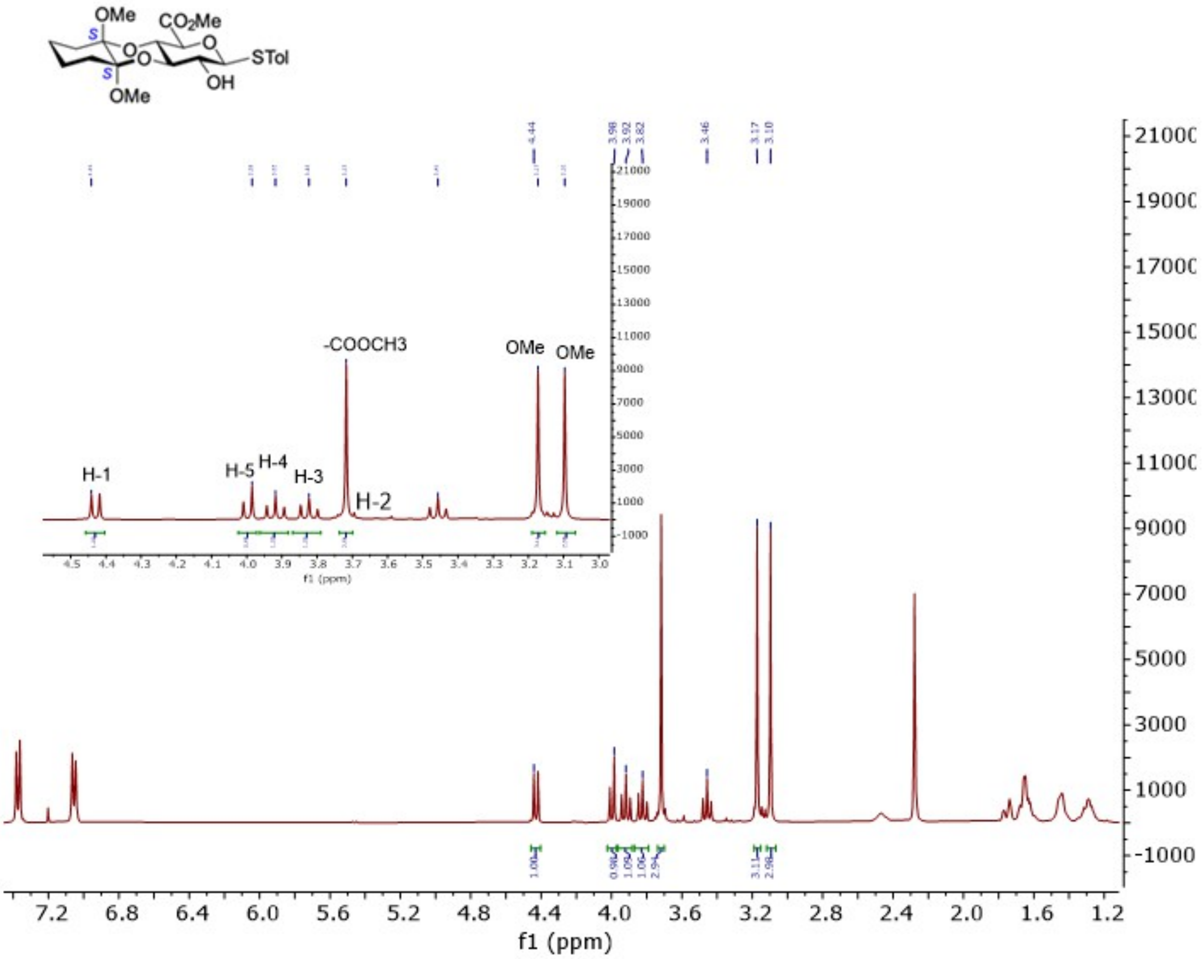

COSY NMR (400 MHz; $CDCl_3$) of phenyl 3,4-*O*-(1,2-dimethoxy-1,2-cyclohexanediyl)-4-methyl-1-thio-6-methyl-β-D-glucuronic acid 3

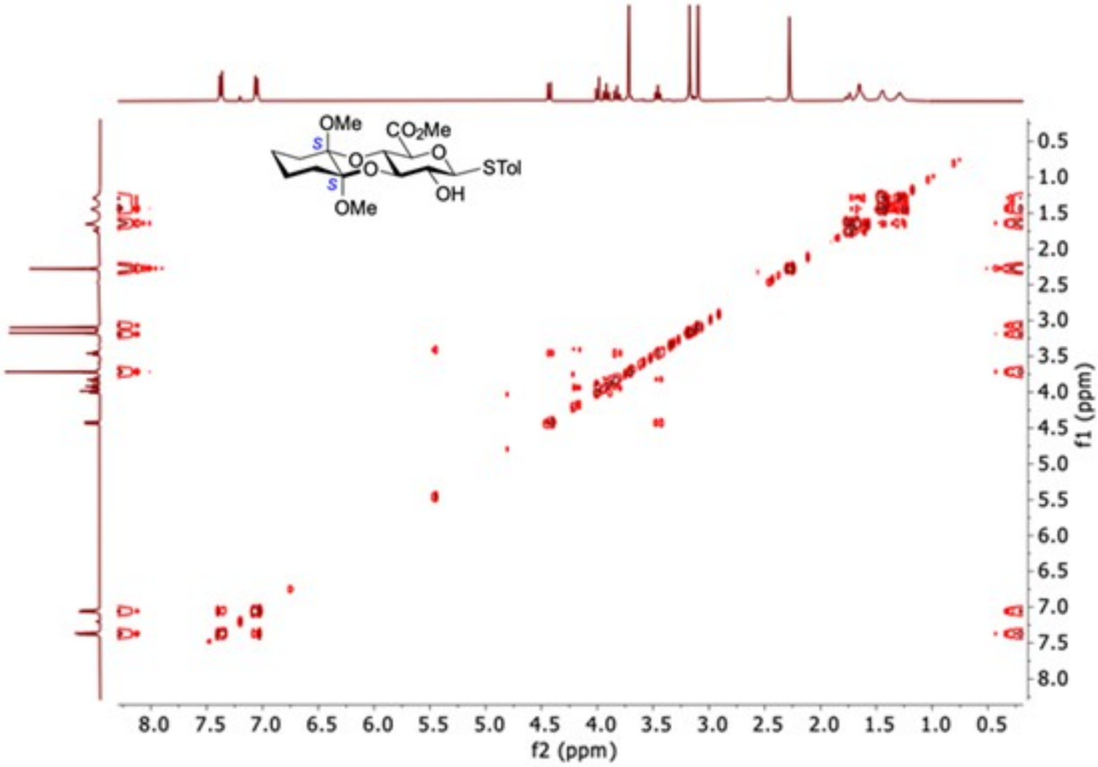

Expansion of COSY NMR of phenyl 3,4-*O*-(1,2-dimethoxy-1,2-cyclohexanediyl)-4-methyl-1-thio-6-methyl-β-D-glucuronic acid 3

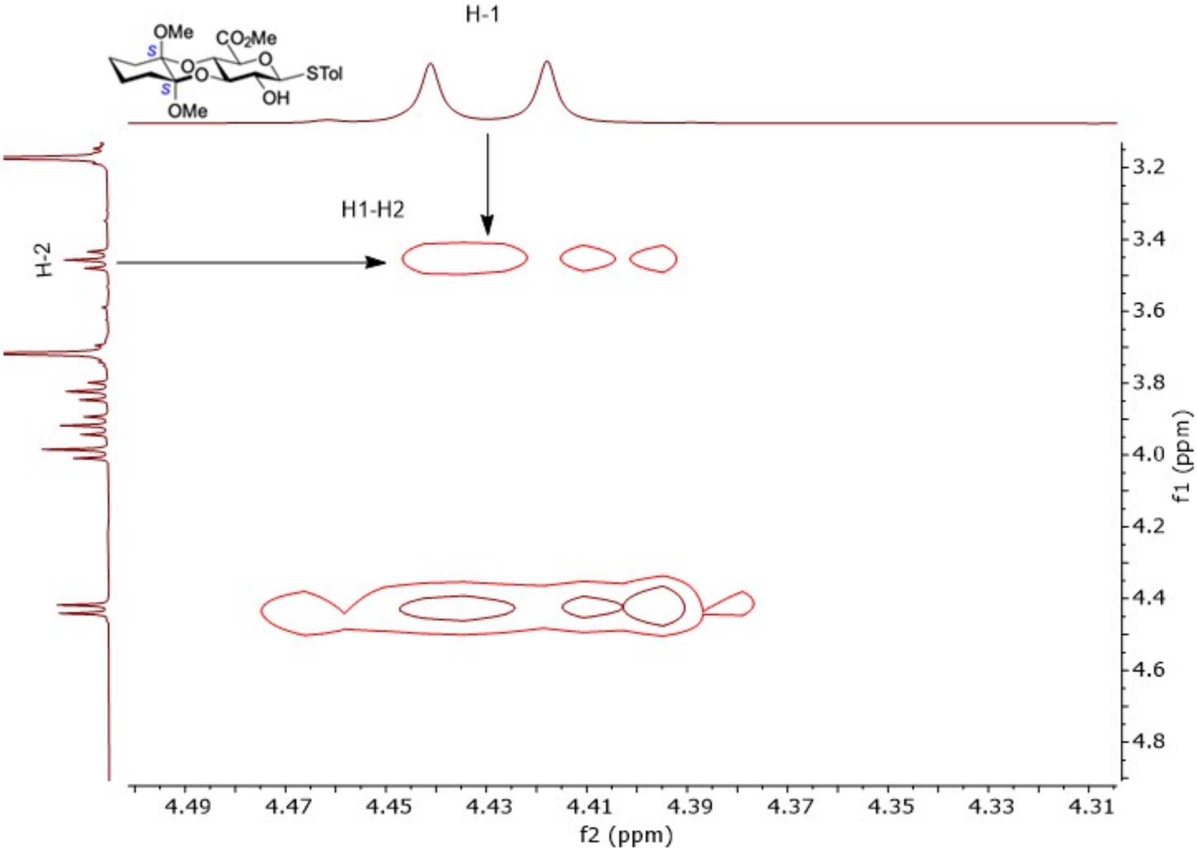

Expansion of COSY NMR of Phenyl 3,4-*O*-(1,2-dimethoxy-1,2-cyclohexanediyl)-4-methyl-1-thio-6-methyl-β-D-glucuronic acid 3

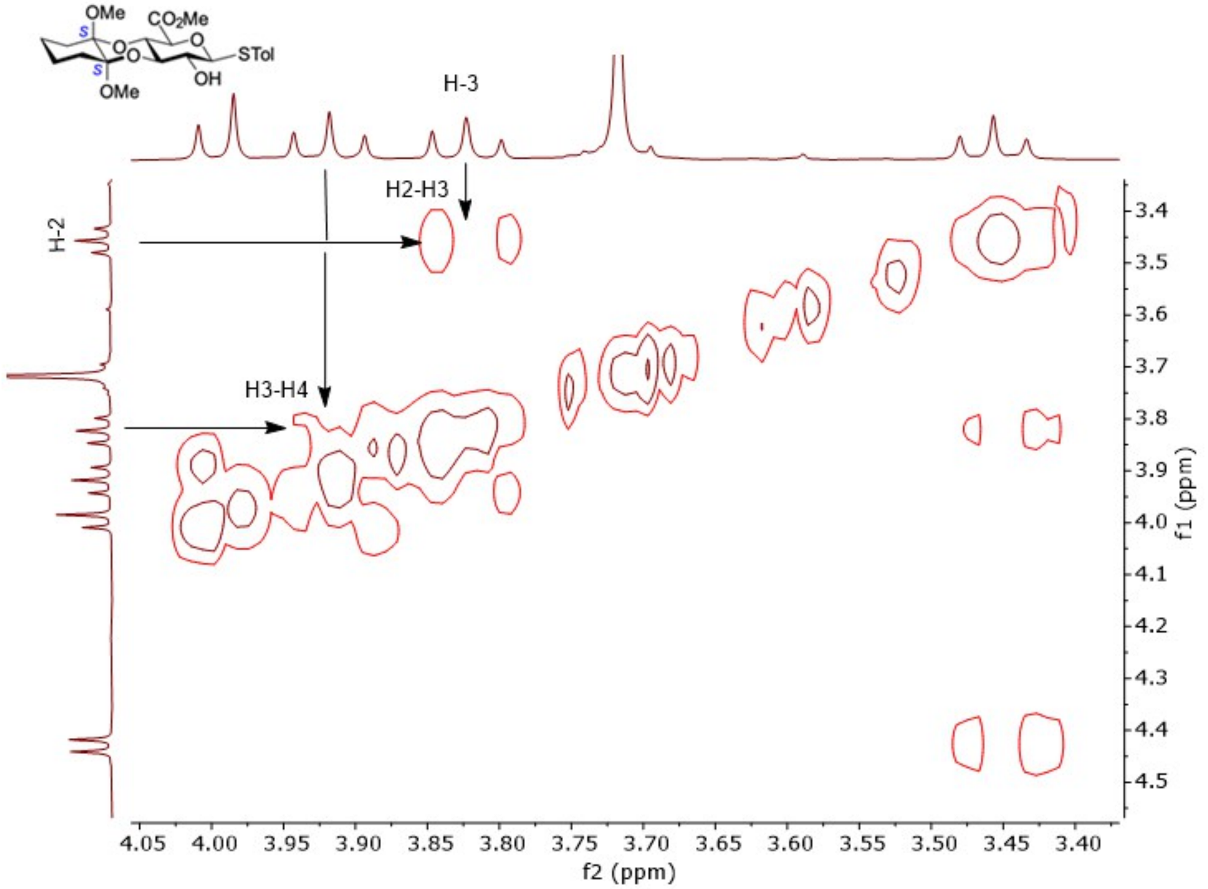

^{13}C NMR (100 MHz; CDCl$_3$) of phenyl 3,4-*O*-(1,2-dimethoxy-1,2-cyclohexanediyl)-4-methyl-1-thio-6-methyl-β-D-glucuronic acid 3

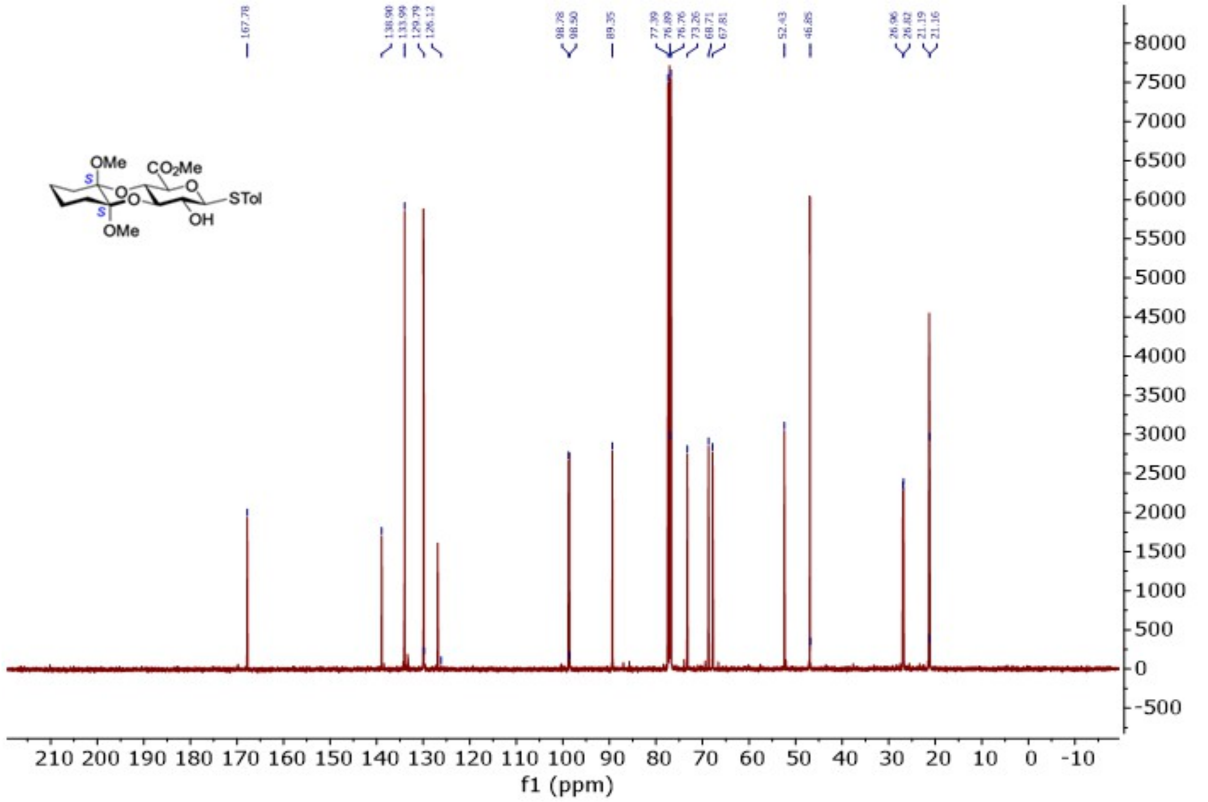

HSQC NMR (400 MHz; $CDCl_3$) of phenyl 3,4-O-(1,2-dimethoxy-1,2-cyclohexanediyl)-4-methyl-1-thio-6-methyl-β-D-glucuronic acid 3

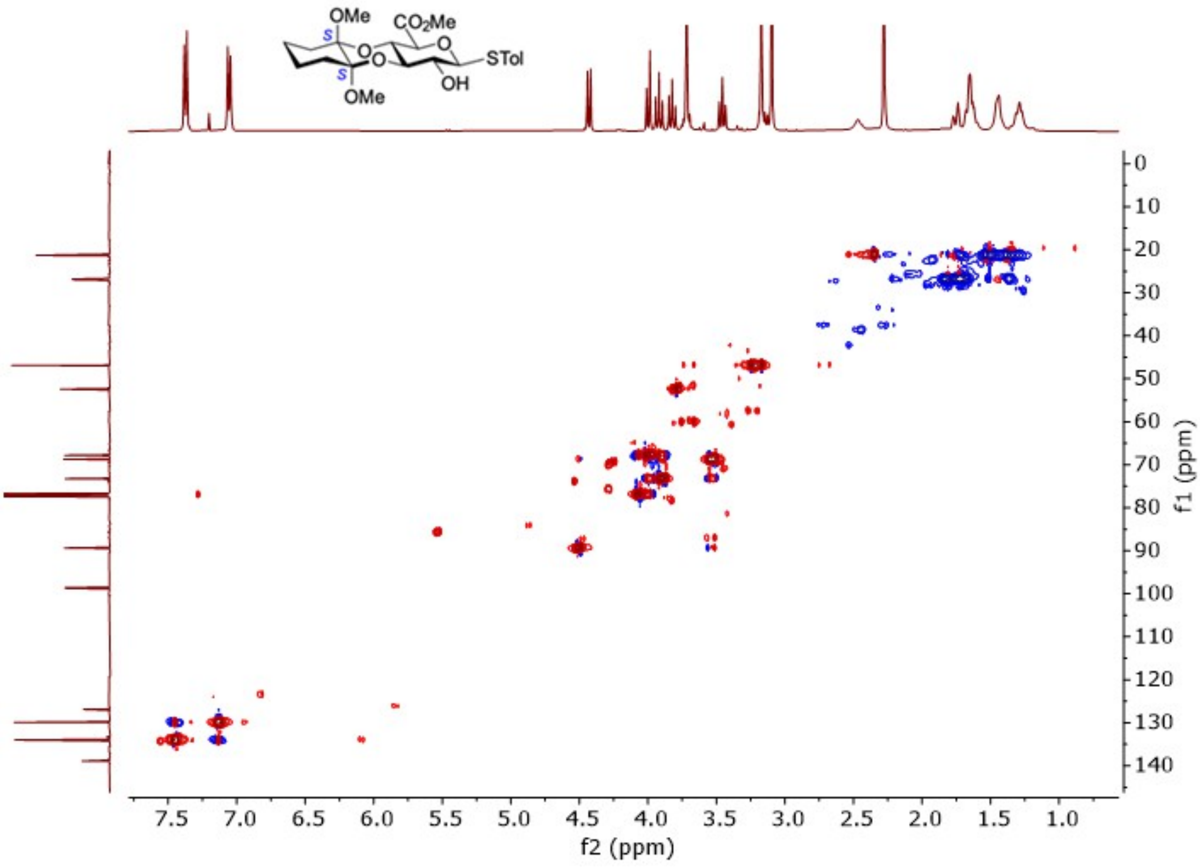

NOESY NMR (400 MHz; $CDCl_3$) of phenyl 3,4-O-(1,2-dimethoxy-1,2-cyclohexanediyl)-4-methyl-1-thio-6-methyl-β-D-glucuronic acid 3

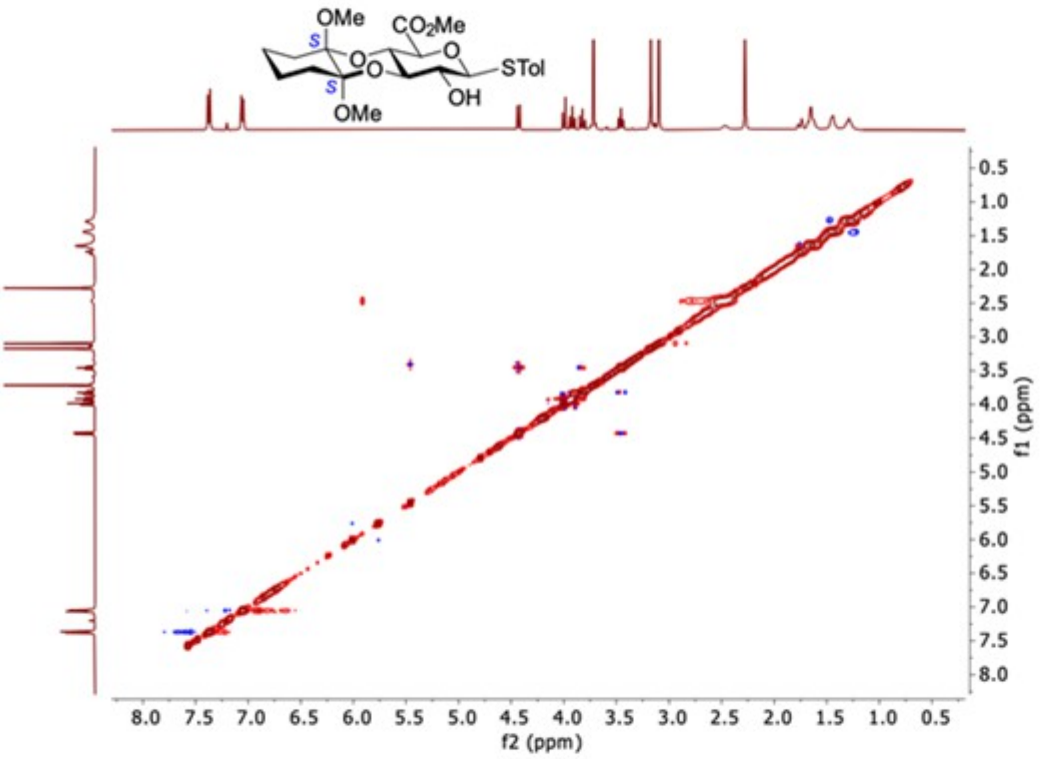

¹H NMR (400 MHz; CDCl₃) spectrum of the GlcA-CDA odd isomer 4

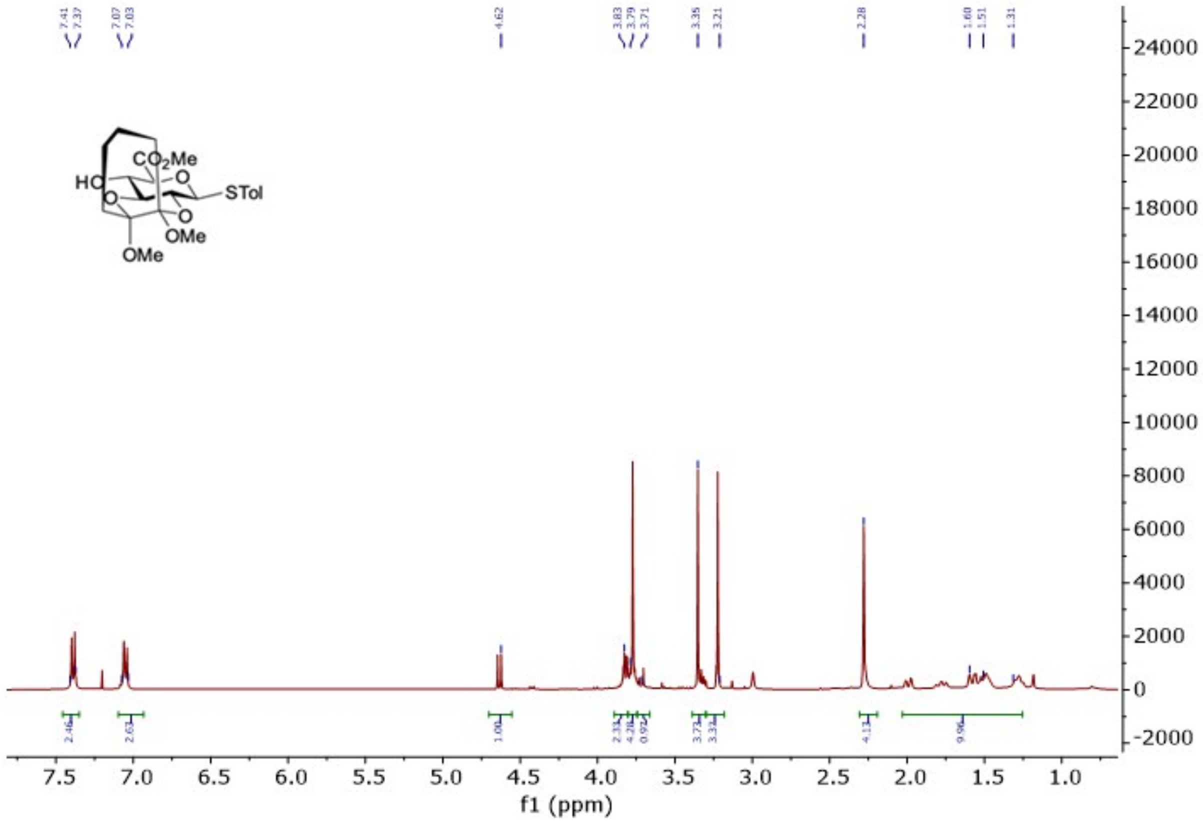

¹H NMR (400 MHz; CDCl₃) spectrum of the GlcA-CDA odd isomer 4 (copy with expansion)

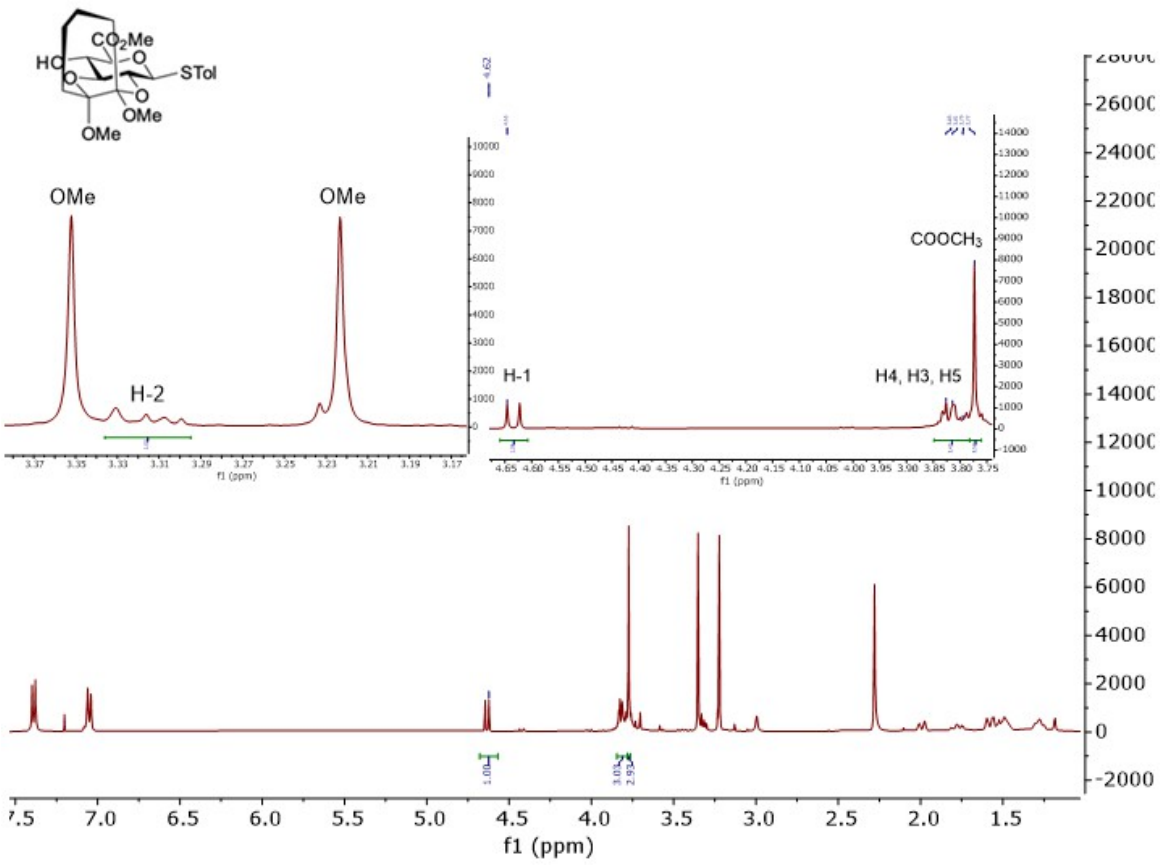

COSY spectrum (400 MHz; $CDCl_3$) of the GlcA-CDA odd isomer 4

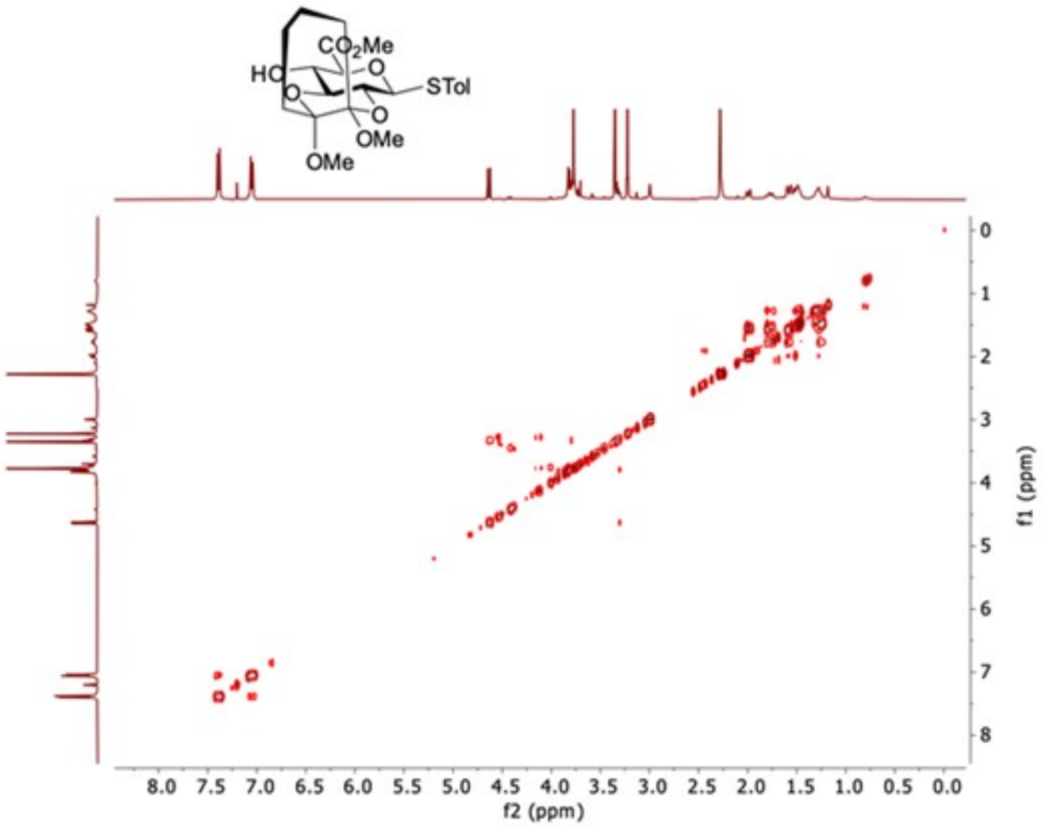

Expansion of COSY spectrum of the GlcA-CDA odd isomer 4

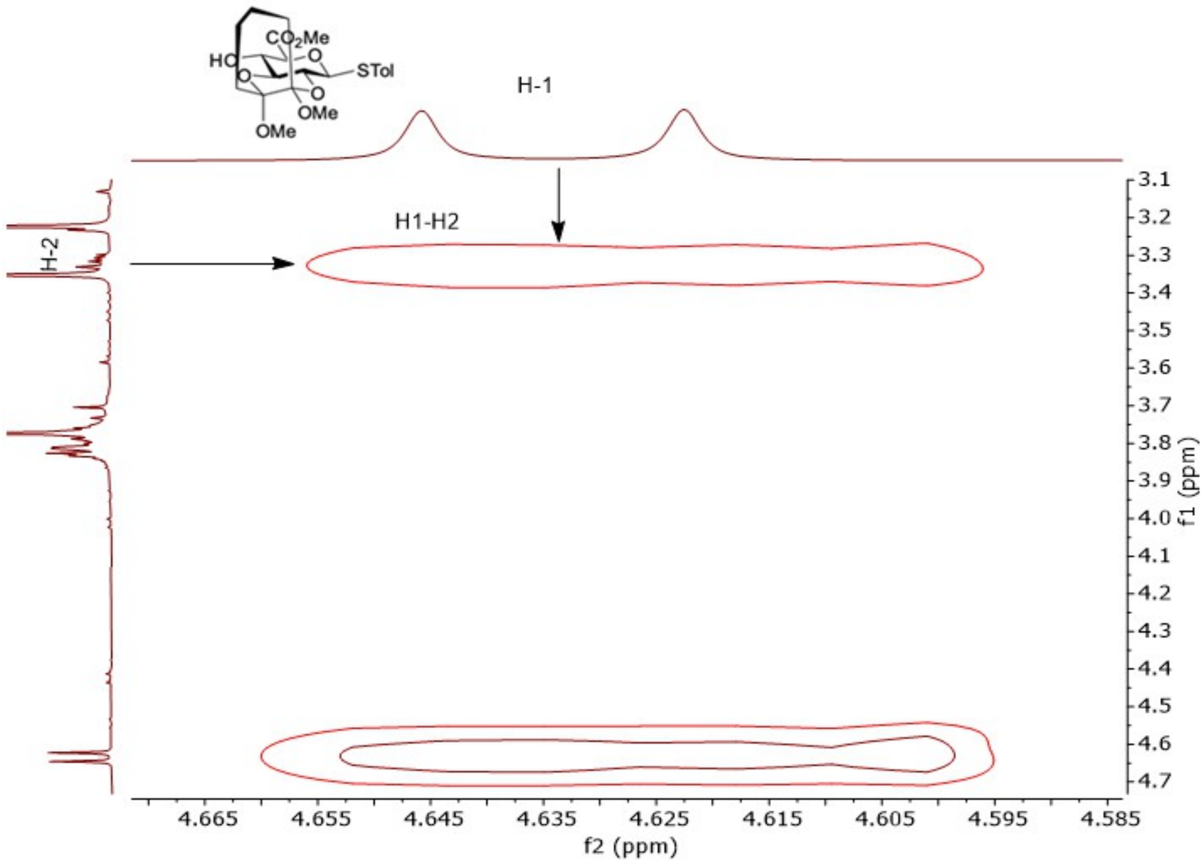

^{13}C NMR spectrum (100 MHz; CDCl$_3$) of the GlcA-CDA odd isomer 4

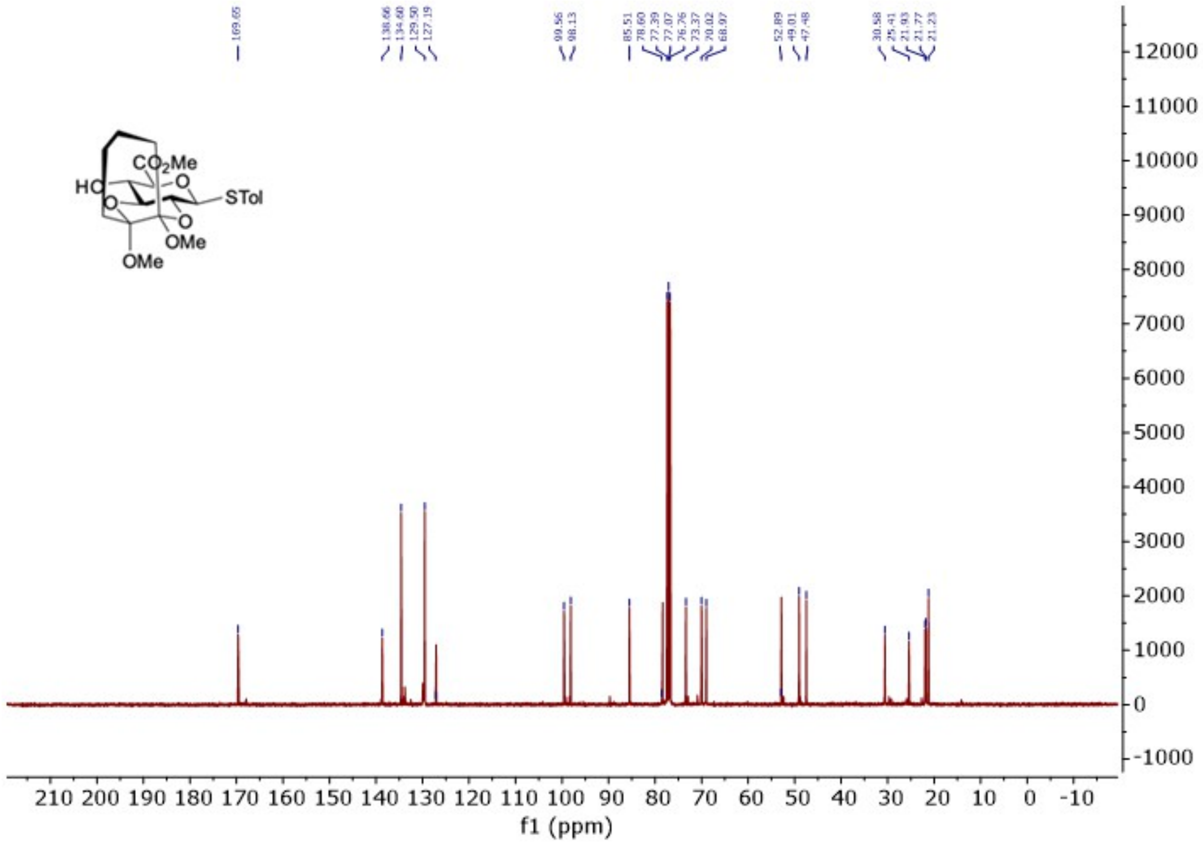

HSQC (400 MHz; CDCl$_3$) spectrum of the GlcA-CDA odd isomer 4

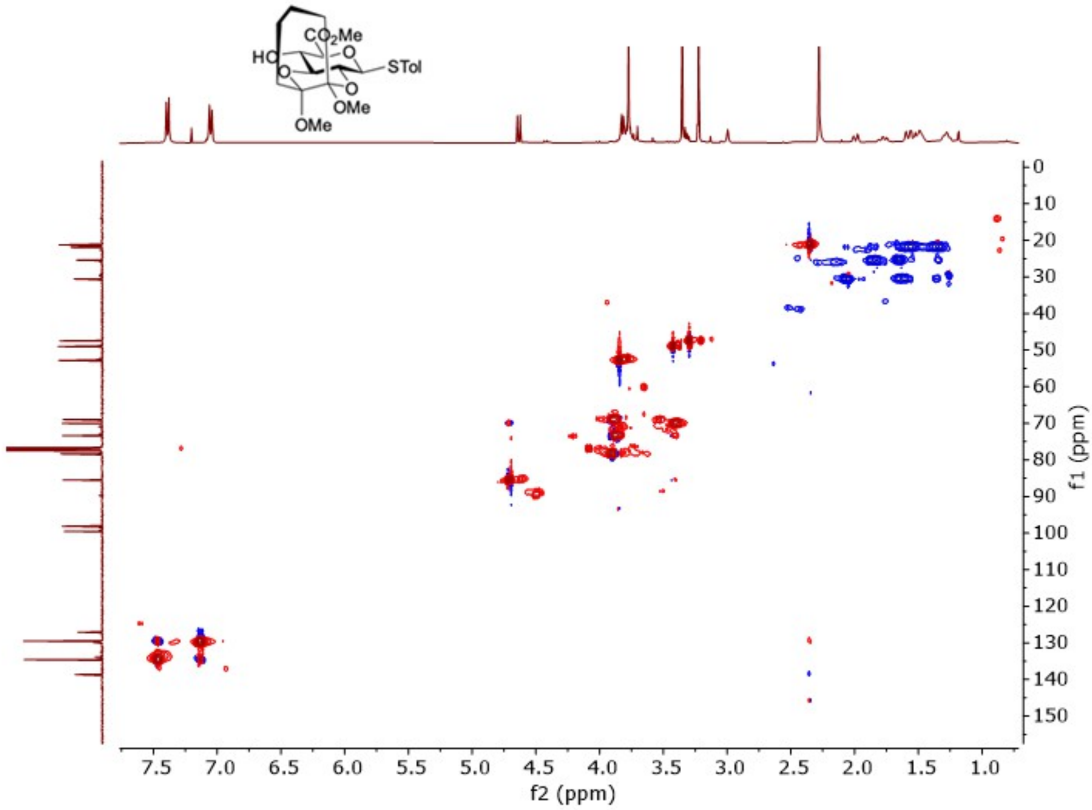

NOESY spectrum (400 MHz; $CDCl_3$) of the GlcA-CDA odd isomer 4

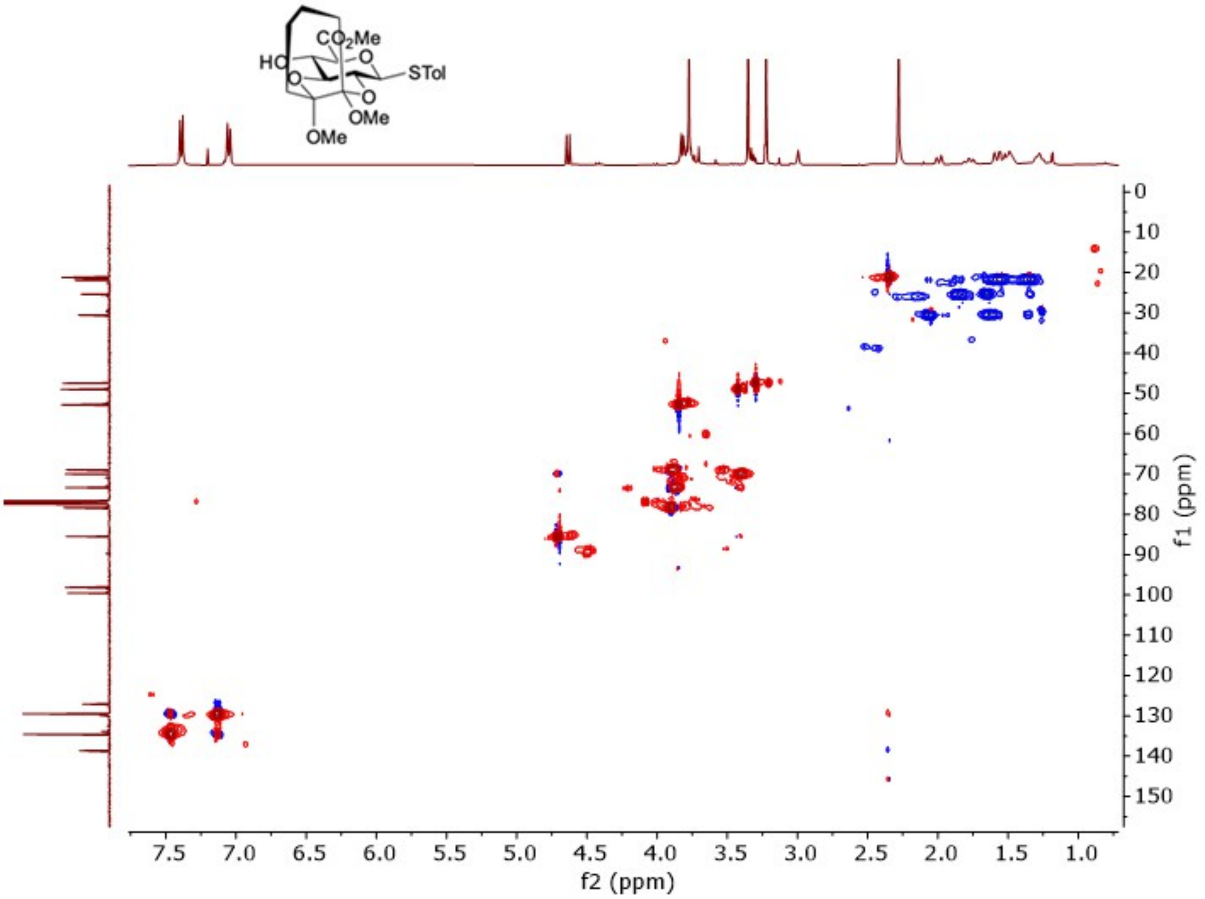

^{1}H NMR (400 MHz; $CDCl_3$) of the phenyl 2,3-O-(1,2-dimethoxy-1,2-cyclohexanediyl)-4-trichloroacetyl-4-methyl-1-thio-6-methyl-β-D-glucuronic acid 5

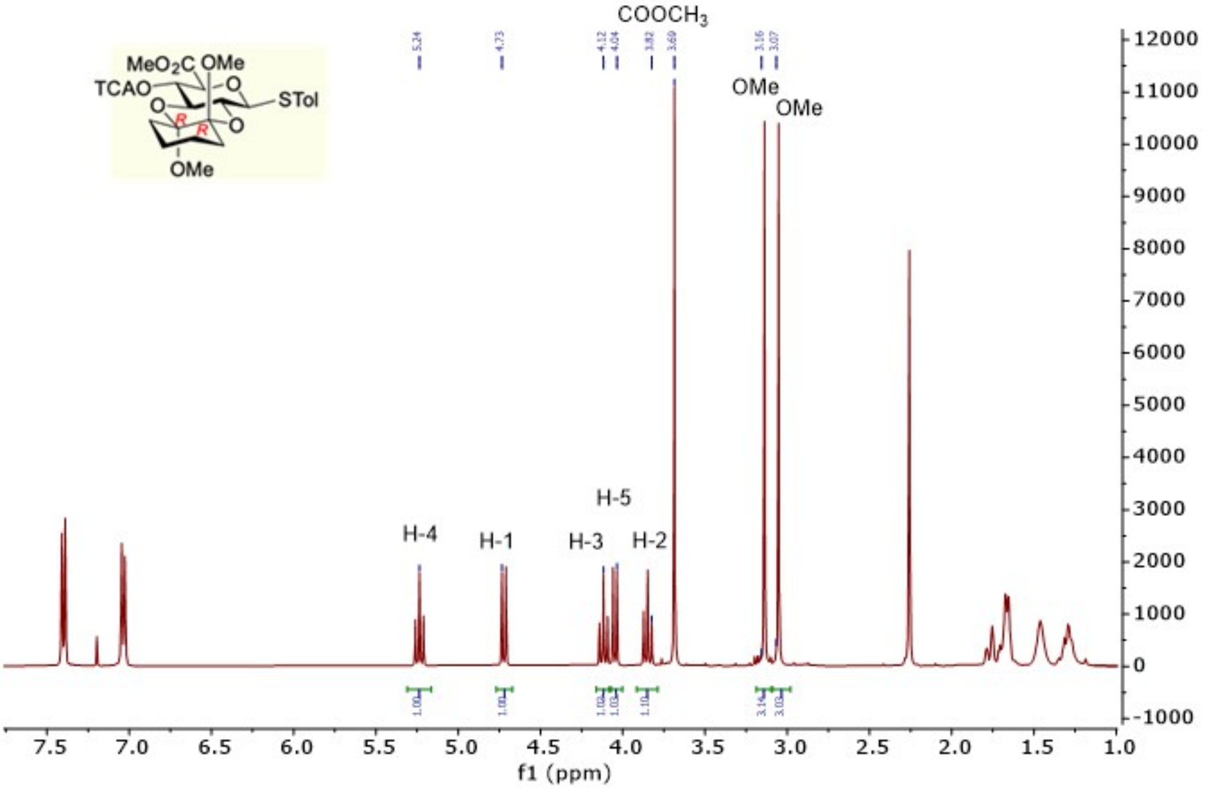

COSY NMR (400 MHz; $CDCl_3$) phenyl 2,3-O-(1,2-dimethoxy-1,2-cyclohexanediyl)-4-trichloroacetyl-4-methyl-1-thio-6-methyl-β-D-glucuronic acid 5

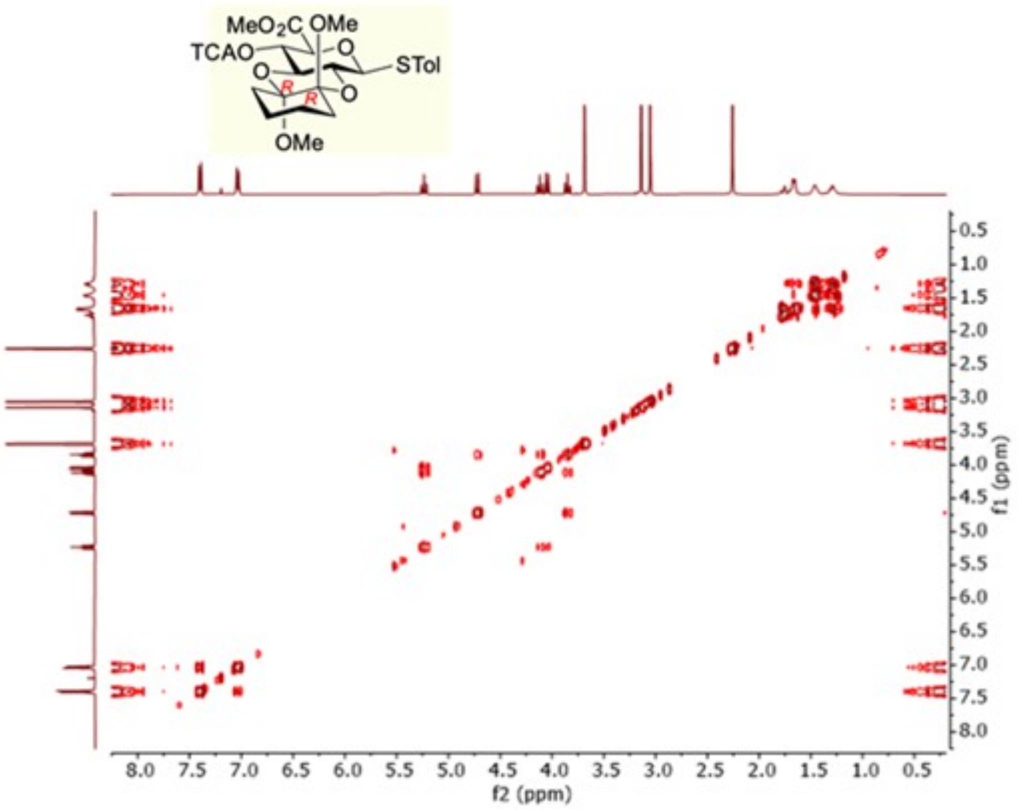

Expansion of COSY NMR Phenyl 2,3-O-(1,2-dimethoxy-1,2-cyclohexanediyl)-4-trichloroacetyl-4-methyl-1-thio-6-methyl-β-D-glucuronic acid 5

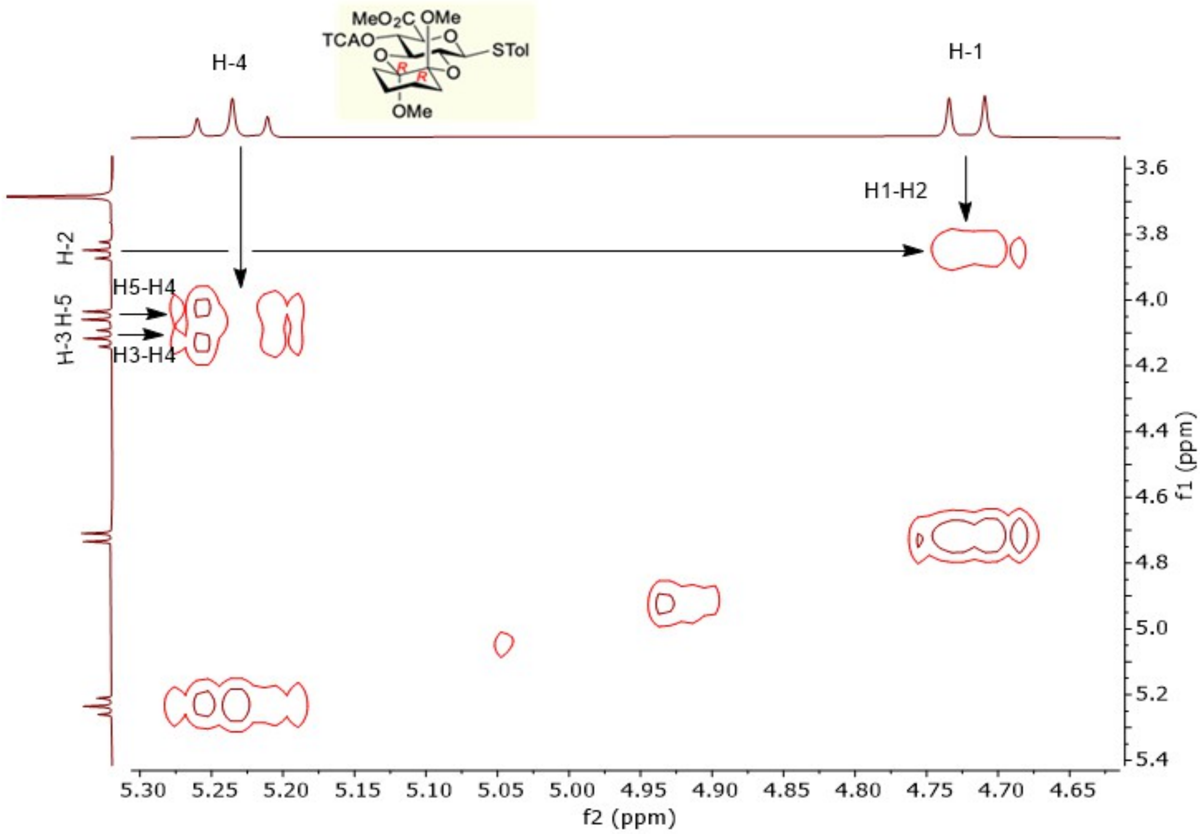

^{13}C NMR (100 MHz; CDCl$_3$) of the phenyl 2,3-O-(1,2-dimethoxy-1,2 cyclohexanediyl)-4-trichloroacetyl-4-methyl-1-thio-6-methyl-β- D-glucuronic acid 5

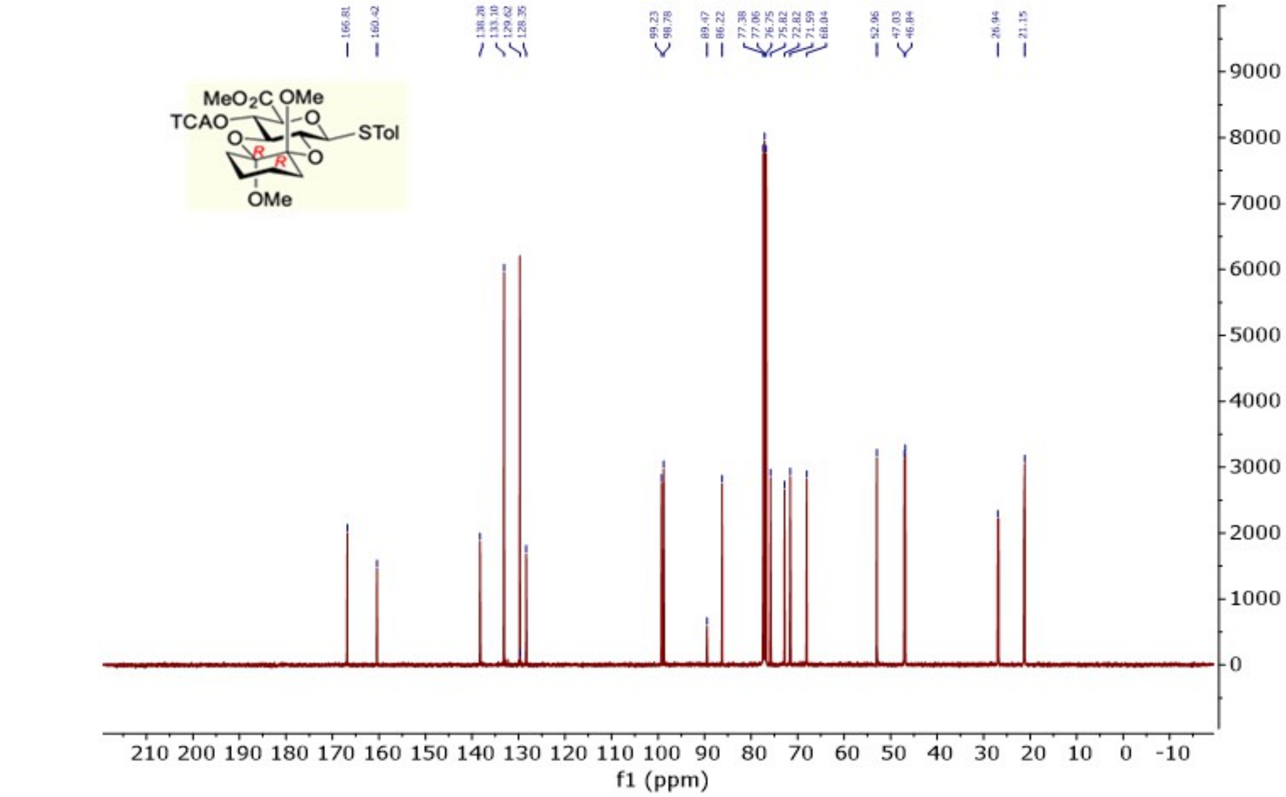

HSQC NMR (400 MHz; CDCl$_3$) of the phenyl 2,3-O-(1,2-dimethoxy-1,2-cyclohexanediyl)-4-trichloroacetyl-4-methyl-1-thio-6-methyl-β-D-glucuronic acid 5

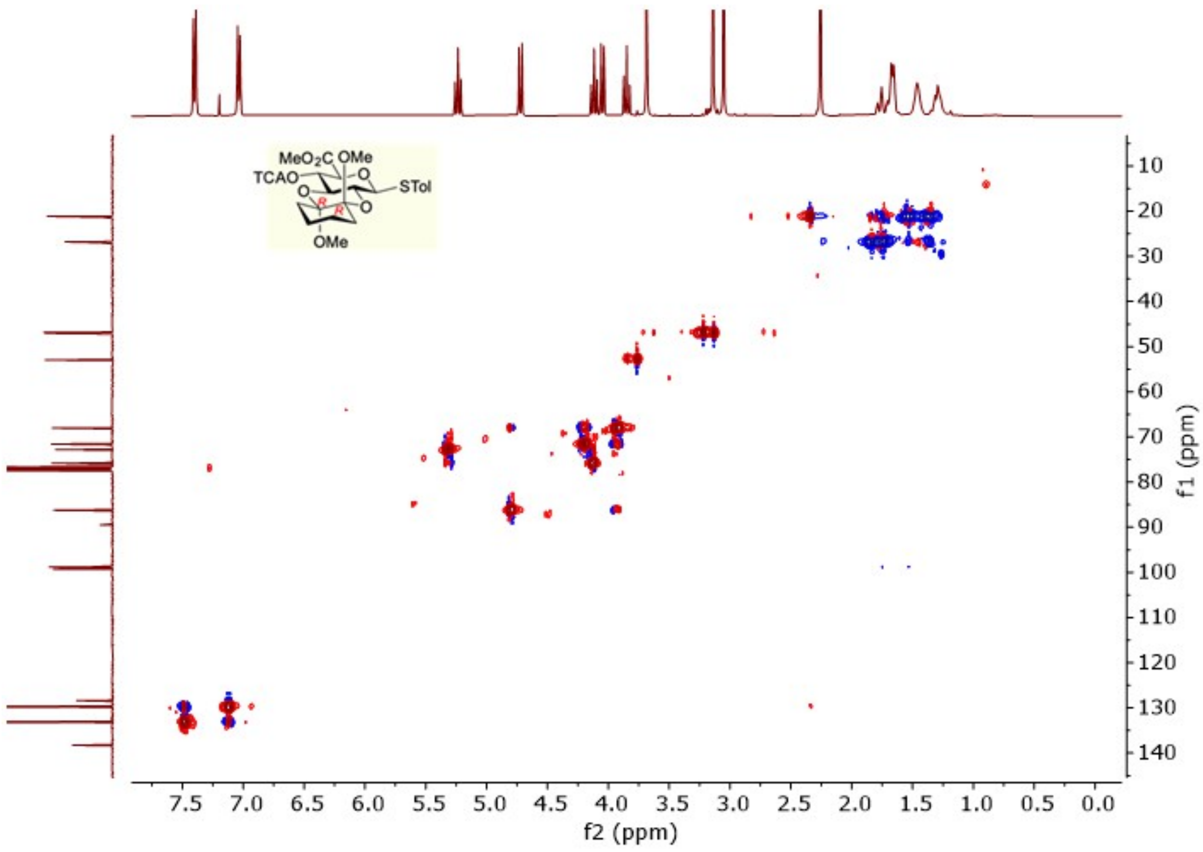

NOESY NMR (400 MHz; $CDCl_3$) of the phenyl 2,3-O-(1,2-dimethoxy-1,2-cyclohexanediyl)-4-trichloroacetyl-4-methyl-1-thio-6-methyl-β-D-glucuronic acid 5

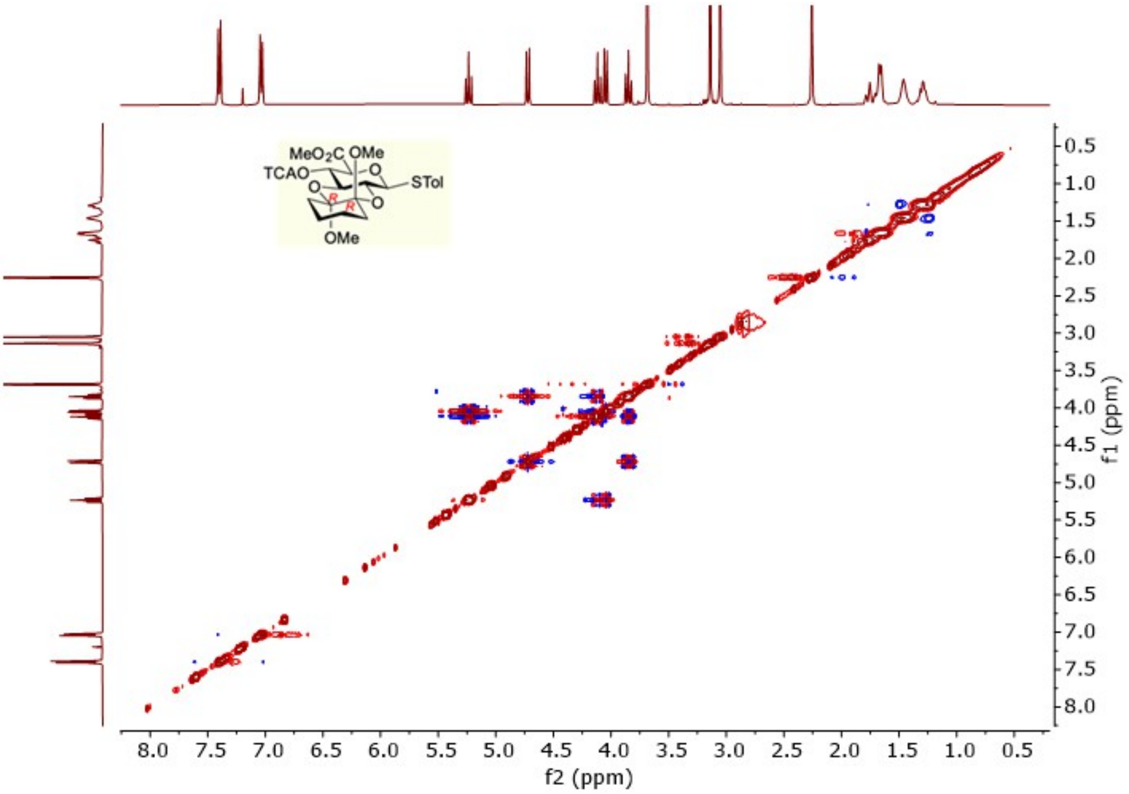

2,3-RS **6** as a colourless liquid: ^{1}H-NMR ($CDCl_3$, 400 MHz) δ 7.46 (d, *J* = 8.1, 2 H, Ar-H), 7.11 (d, *J* = 7.9, 2 H, Ar-H), 4.71 (appt. d, *J* = 9.6 Hz, 1 H, H-1), 4.06-4.01 (m, 1 H, H-3), 3.88-.84 (m, 2 H, H-4 & H-5), 3.84 (s, 3 H, -$COOCH_3$), 3.77-3.74 (m, 1 H, H-2), 3.40 (s, 3 H, -$COCH_3$), 3.22 (s, 3 H, -$COCH_3$), 2.33 (s, 3 H, Ar-CH_3), 1.90-1.85 (m, 1 H, -CH_2-), 1.70-1.57 (m, 3 H, -CH_2-), 1.45-1.37 (m, 2 H, -CH_2-), 1.43-1.33 (m, 2 H,-CH_2-).

^{13}C-NMR ($CDCl_3$, 100 MHz) δ 168.9 (C6), 138.0 (<u>A</u>r-CH_3), 132.6 (2 x Ar-H), 129.7 (2 x Ar-H), 125.6 (Ar), 99.3 (-$COCH_3$), 98.7 (-$COCH_3$), 86.7 (C1), 78.3 (C5), 77.2 (C4), 69.4 (C3), 67.7 (C2), 53.1 (-$COOCH_3$), 49.2 (-$COCH_3$), 47.2 (-$COCH_3$), 30.4 (-CH_2-), 26.4 (-CH_2-), 22.4 (-CH_2-), 21.8 (-CH_2-), 21.4 (Ar-CH_3).

^{1}H NMR (400 MHz; CDCl$_3$) spectrum of the 2,3-RS GlcA-CDA isomer 6

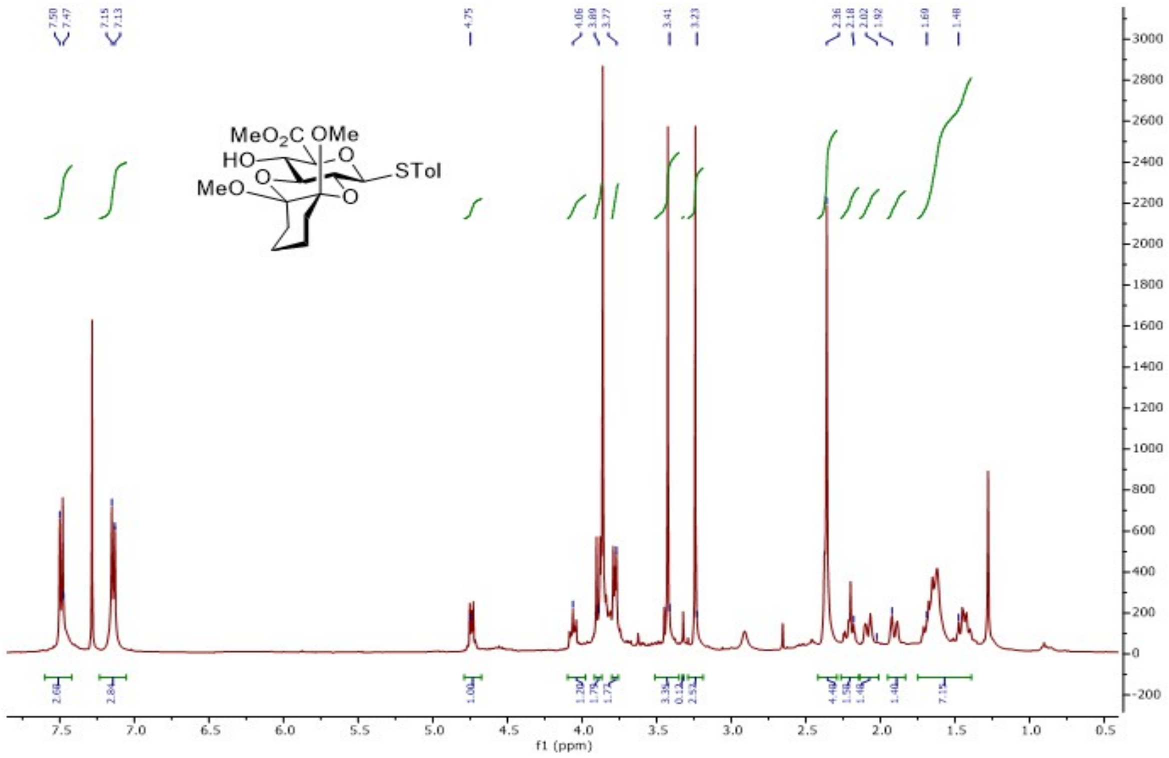

COSY NMR (400 MHz; CDCl$_3$) spectrum of the 2,3-RS GlcA-CDA isomer 6

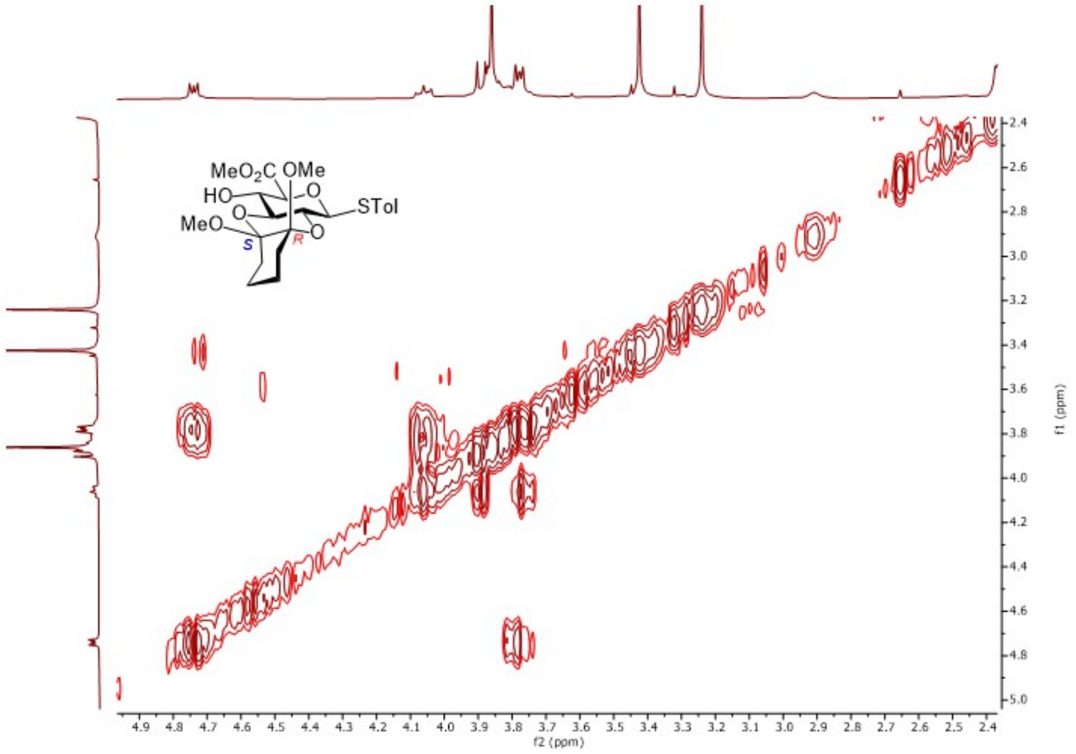

^{13}C NMR (400 MHz; $CDCl_3$) spectrum of the 2,3-RS GlcA-CDA isomer 6

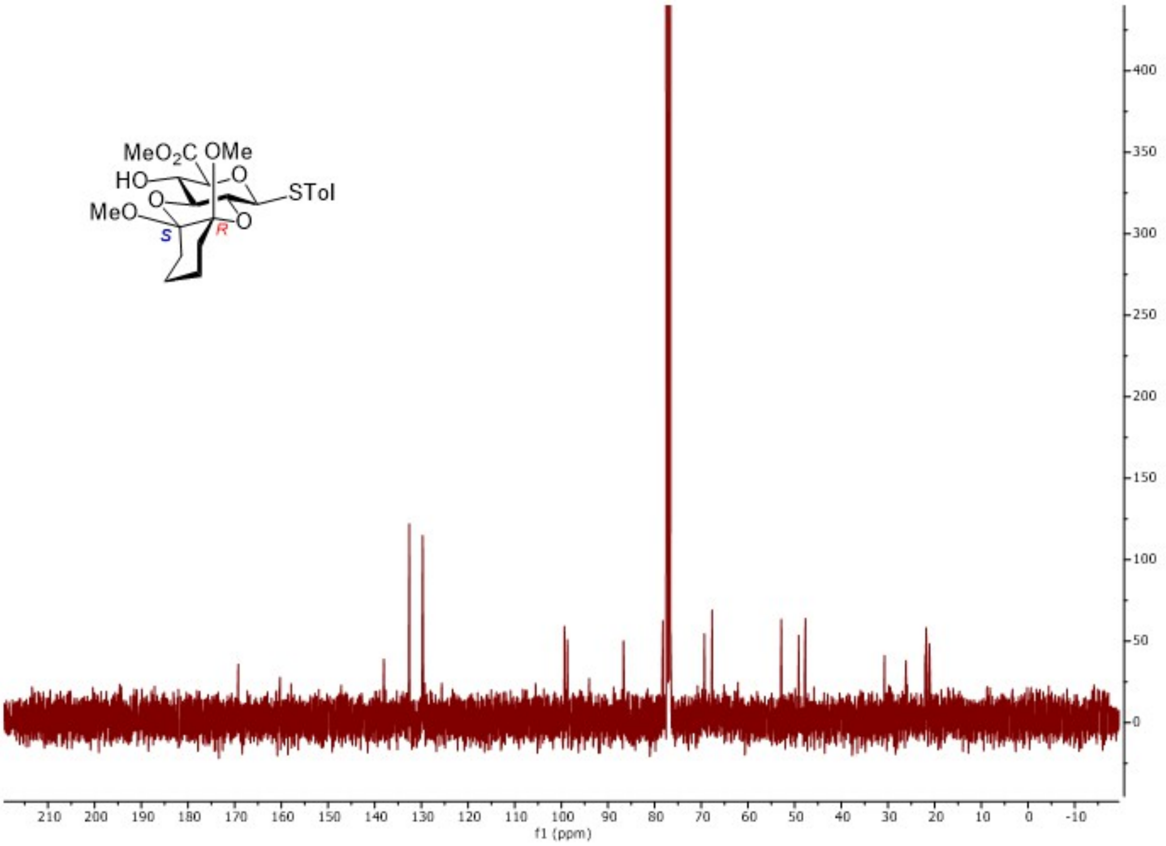

HSQC NMR (400 MHz; $CDCl_3$) spectrum of the 2,3-RS GlcA-CDA isomer 6

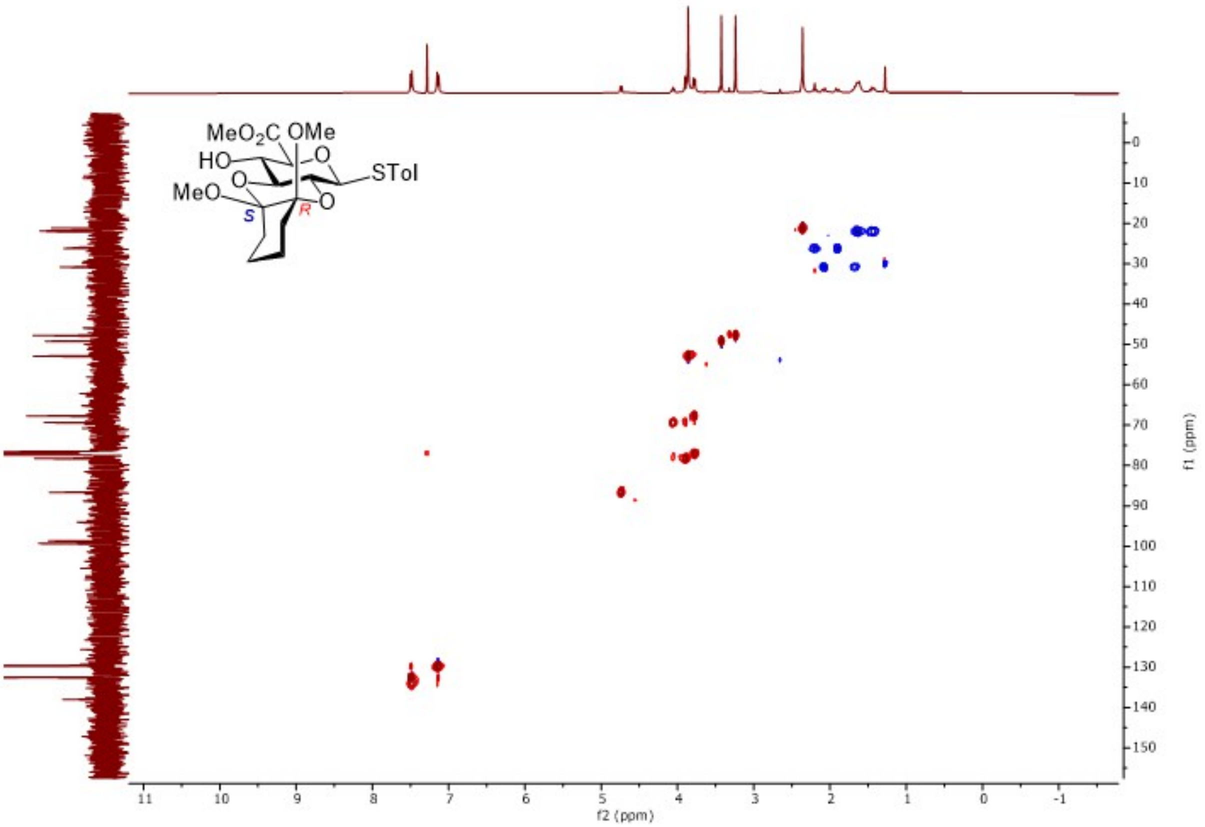

NOESY NMR (400 MHz; $CDCl_3$) spectrum of the 2,3-RS GlcA-CDA isomer 6

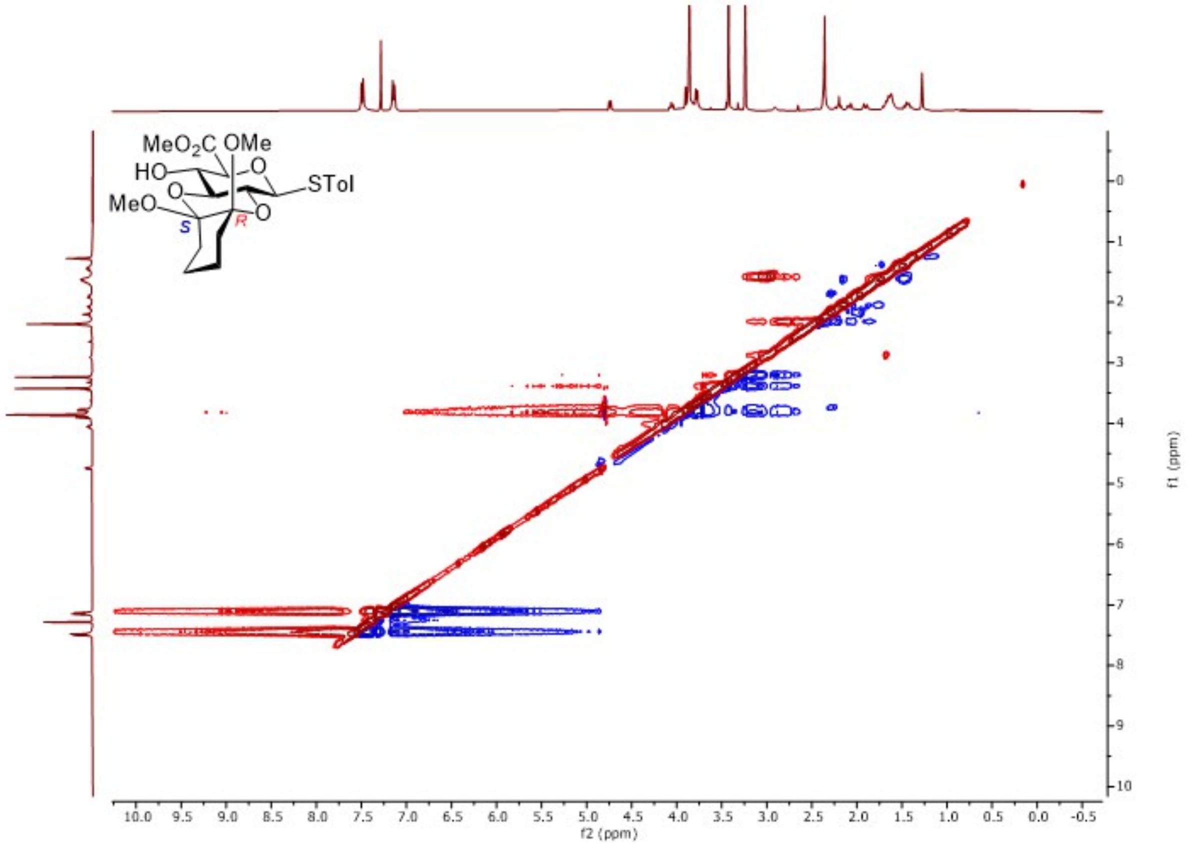

Expansion of NOESY NMR to confirm identity of 2,3-RS GlcA-CDA isomer 6

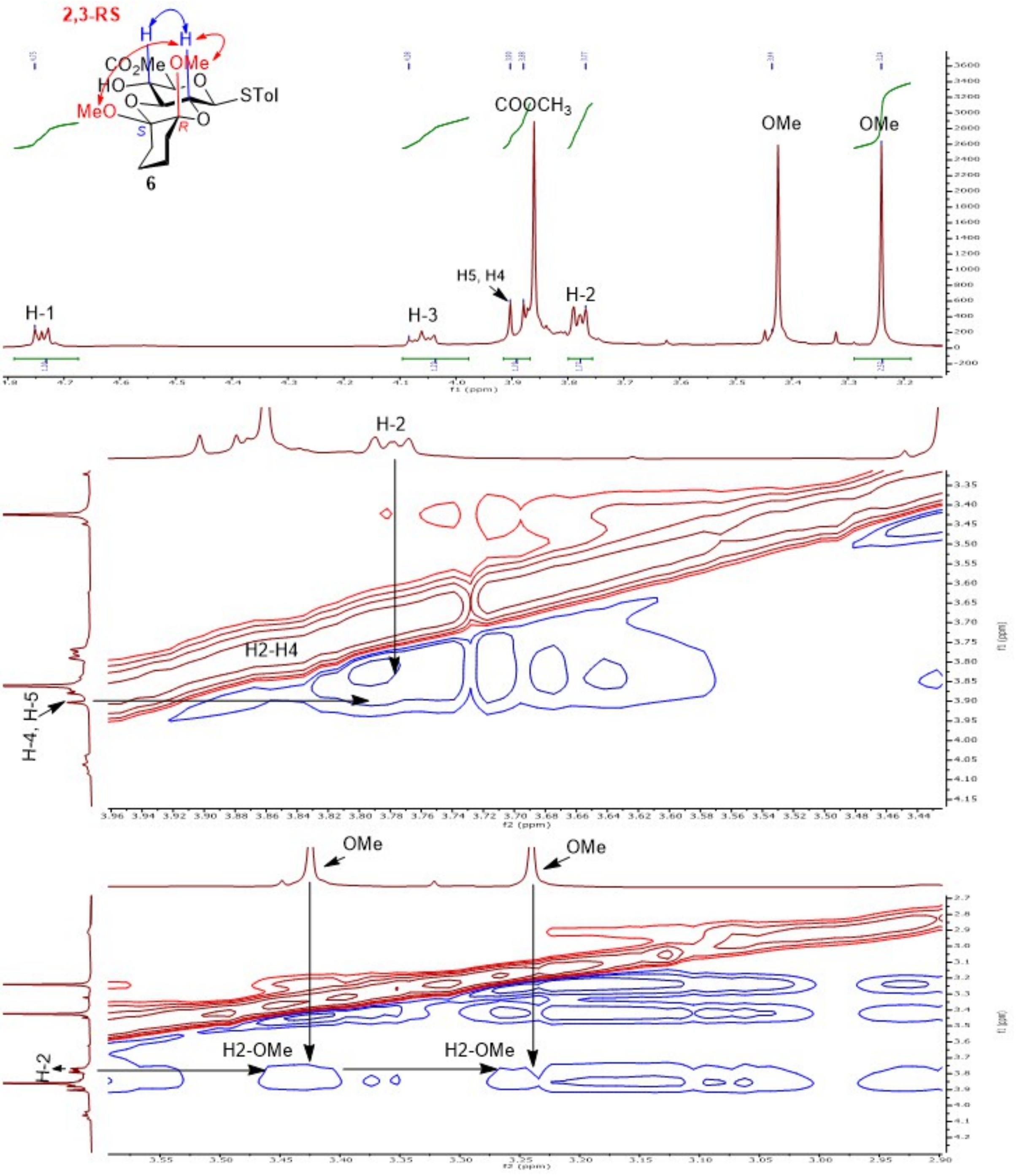

^{1}H NMR spectrum of either the 3,4-RR 7 or the 2,3-SS isomer 8

A colourless liquid either 3,4-RR **7** or 2,3-SS **8:** ^{1}H-NMR ($CDCl_3$, 400 MHz) δ 7.45 (d, J = 8.1, 2 H, Ar-H), 7.11 (d, J = 7.9, 2 H, Ar-H), 4.91 (d, J = 9.3 Hz, 1 H, H-1), 4.08 (d, J = 8.9 Hz, 1 H, H-5), 3.89-3.80 (m, 2 H, H-4 & H-3), 3.78 (s, 3 H, -COOCH$_3$), 3.51 (t, J = 9.2 Hz, 1 H, H-2), 3.31 (s, 3 H, -COCH$_3$), 3.29 (s, 3 H, -COCH$_3$), 2.36 (s, 3 H, Ar-CH$_3$), 2.17-2.03 (m, 2 H, -CH$_2$-), 1.81-1.75 (m, 1 H, -CH$_2$-), 1.68-1.60 (m, 3 H, -CH$_2$-), 1.43-1.32 (m, 2 H,-CH$_2$-); ^{13}C-NMR ($CDCl_3$, 100 MHz) δ 167.8 (C6), 138.9 (<u>A</u>r-CH$_3$), 133.8 (2 x Ar-H), 130.0 (2 x Ar-H), 127.1 (Ar), 99.3 (-COCH$_3$), 98.4 (-COCH$_3$), 89.8 (C1), 77.2 (C5), 72.9 (C4), 70.1 (C3), 69.8 (C2), 52.2 (-COOCH$_3$), 48.9 (-COCH$_3$), 47.6 (-COCH$_3$), 30.8 (-CH$_2$-), 25.9 (-CH$_2$-), 22.0 (-CH$_2$-), 21.8 (-CH$_2$-), 21.3 (Ar-CH

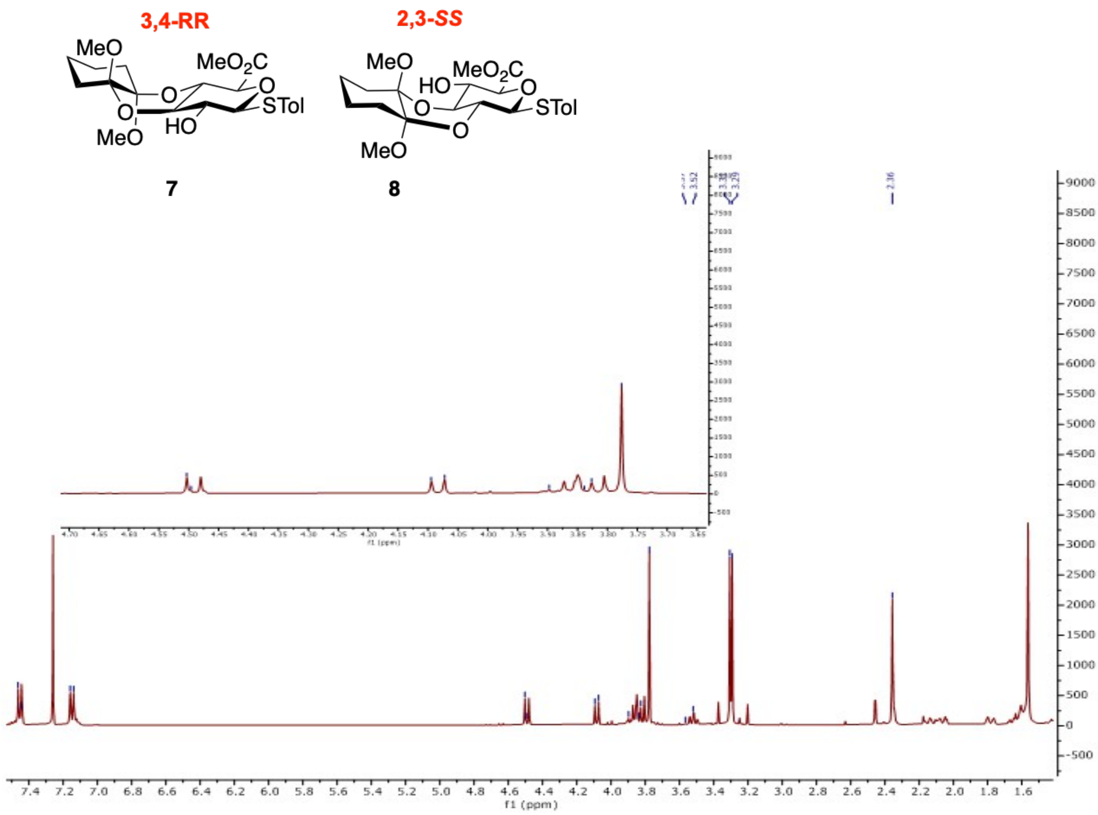

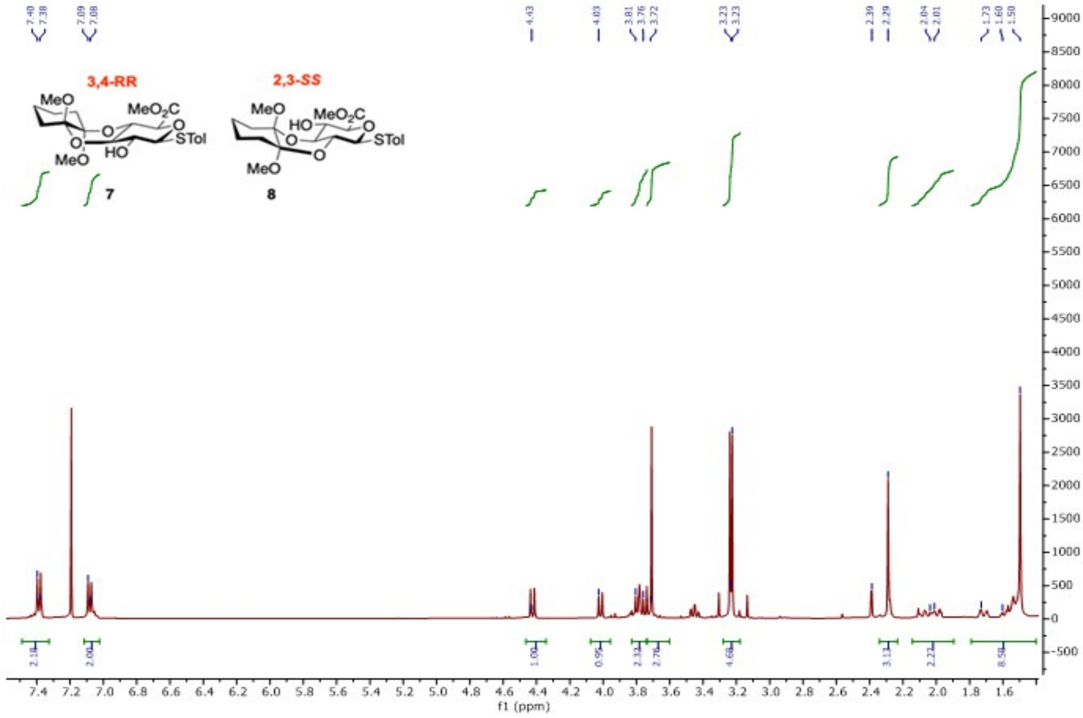

COSY NMR (400 MHz; $CDCl_3$) spectrum of either the 3,4-RR 7 or the 2,3-SS isomer 8

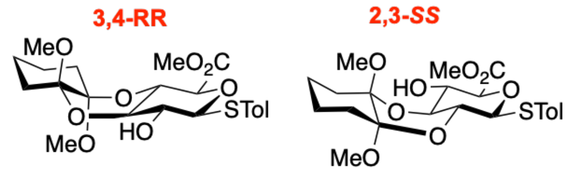

7 8

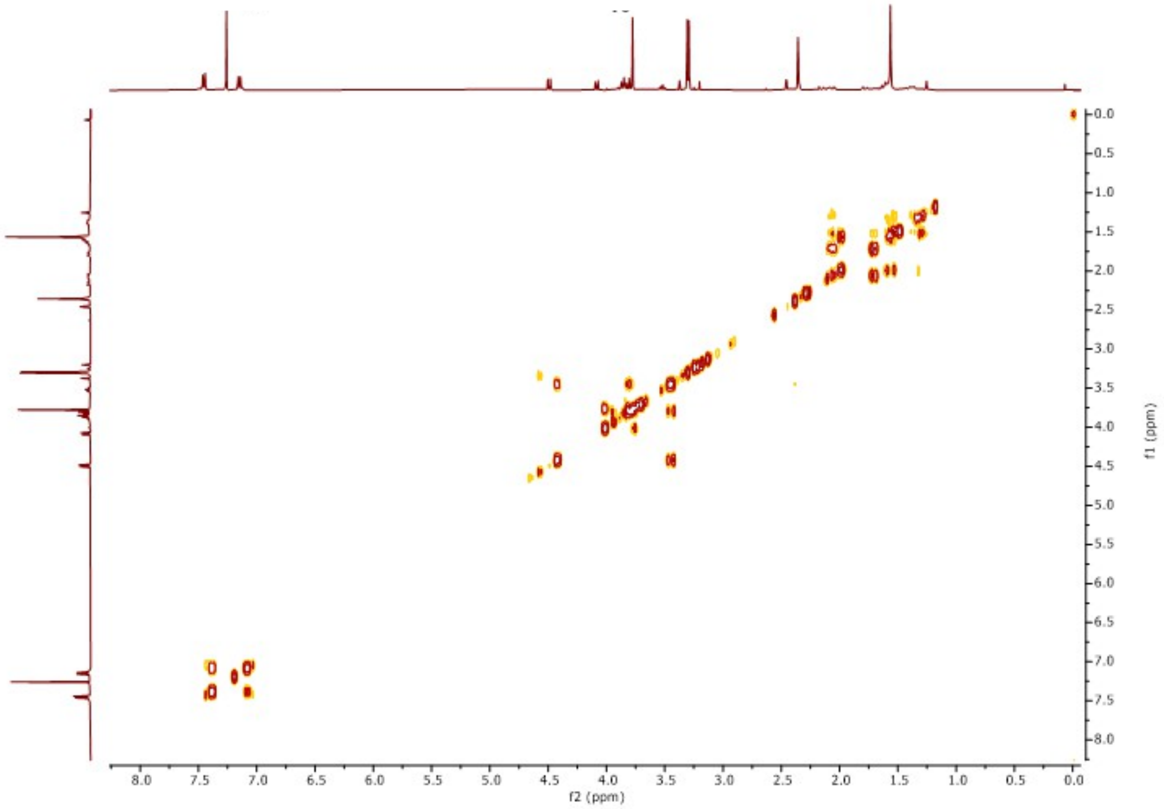

^{13}C NMR (400 MHz; CDCl3) spectrum of either the 3,4-RR 7 or the 2,3-SS isomer 8

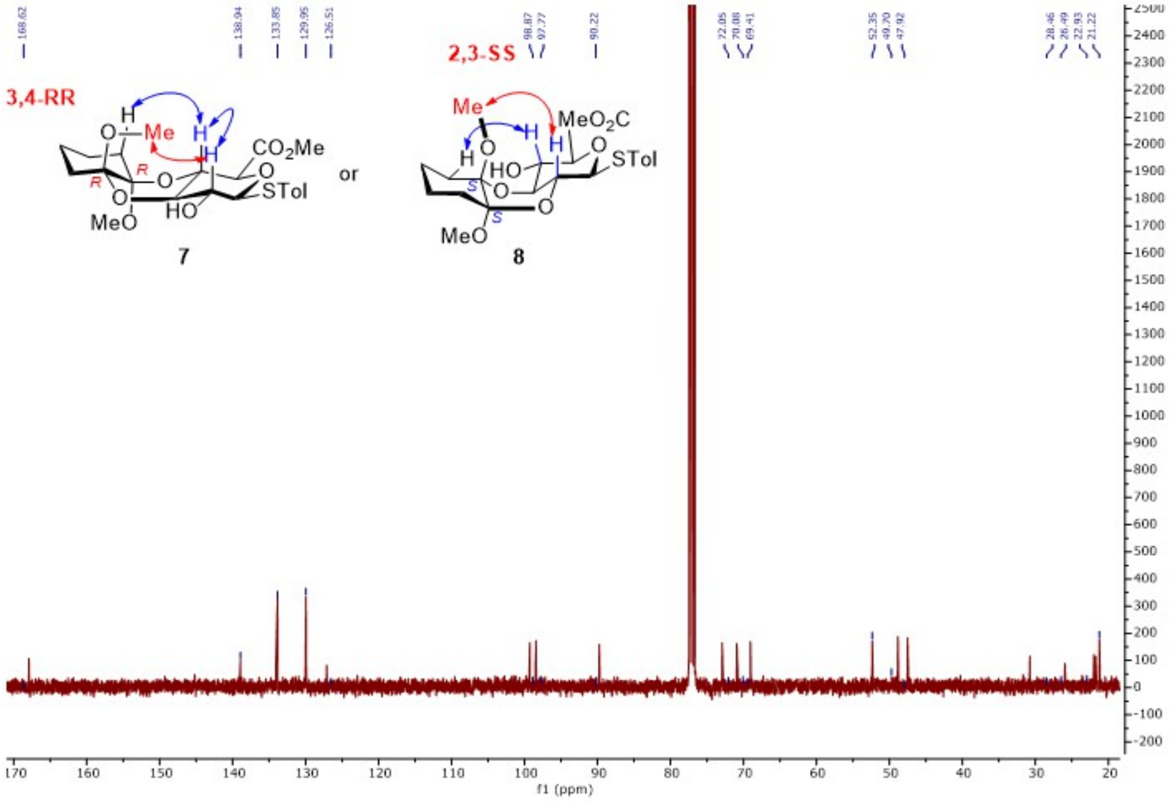

HSQC NMR (400 MHz; CDCl3) spectrum of either the 3,4-RR 7 or the 2,3-SS isomer 8

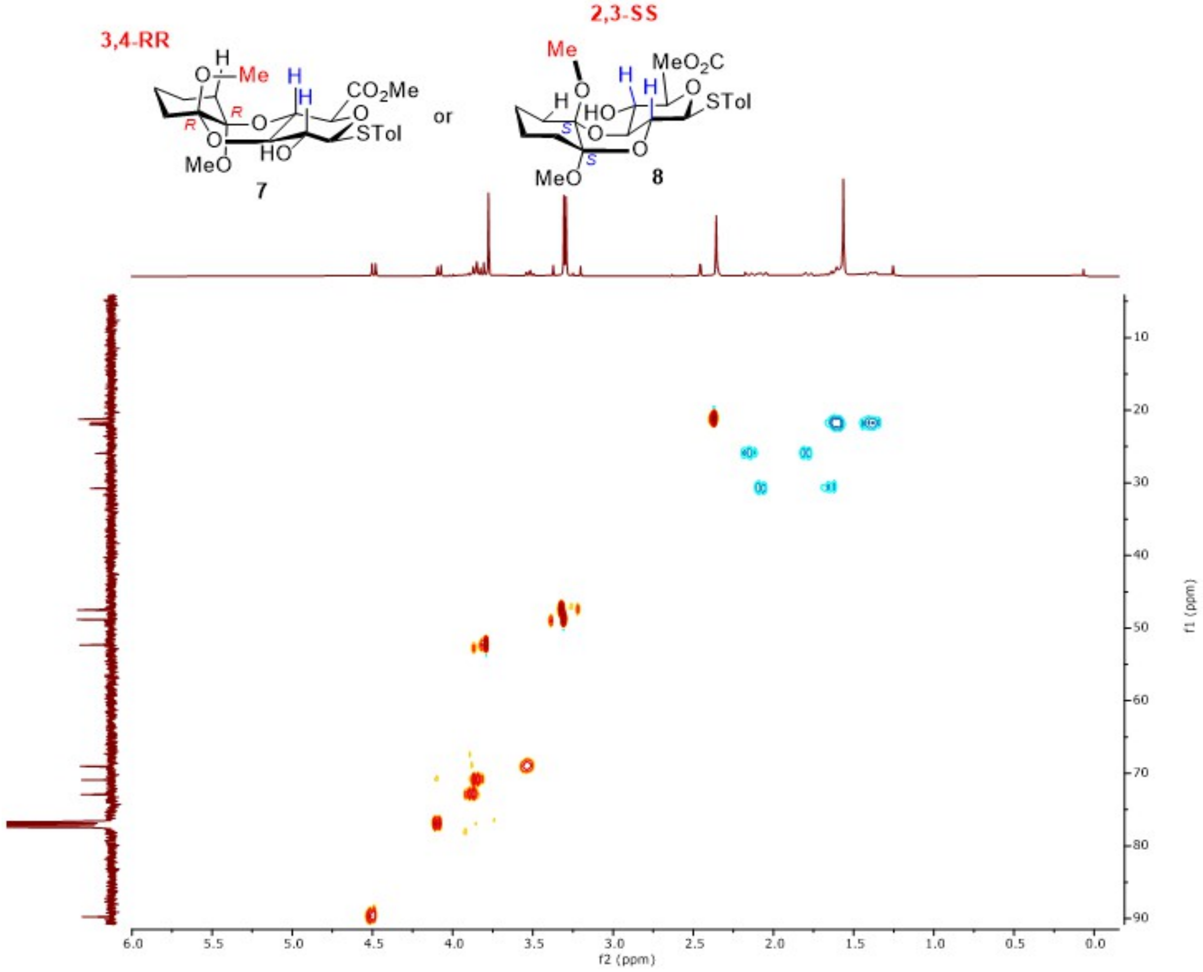

NOESY NMR (400 MHz; $CDCl_3$) spectrum of either the 3,4-RR 7 or the 2,3-SS isomer 8

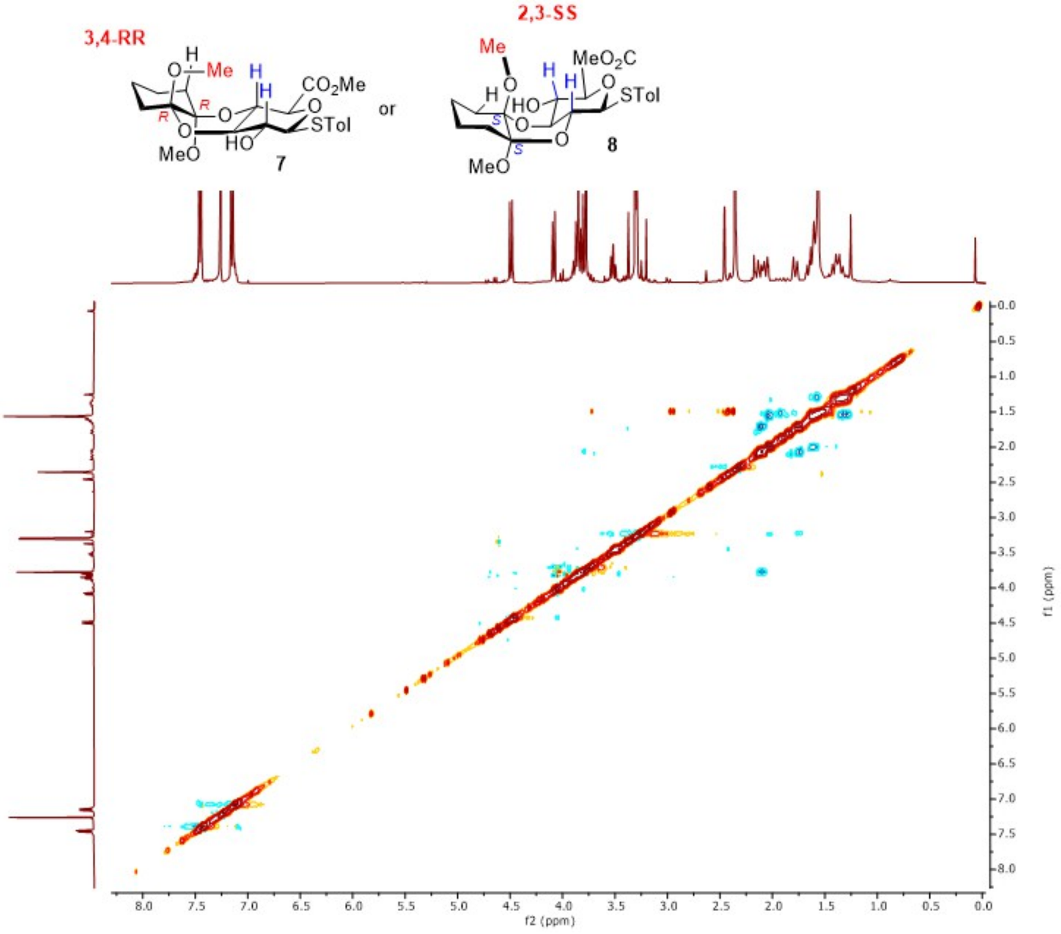

Expansion of NOESY NMR spectrum to confirm identity of the 2,3-SS isomer 8 to show H2-OMe and H4-cyclohexane CH_2 interactions

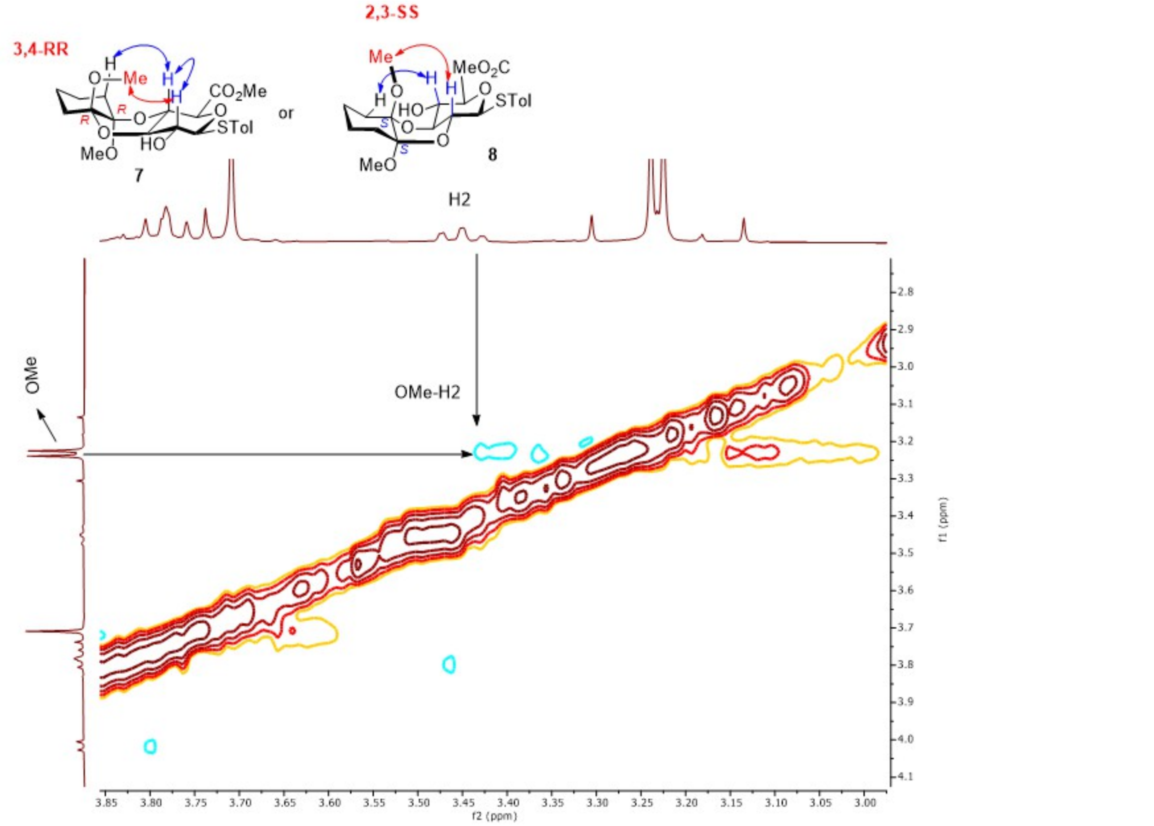

Expansion of NOESY of either the 3,4-RR 7 or the 2,3-SS isomer 8 to show H2-H4 interactions

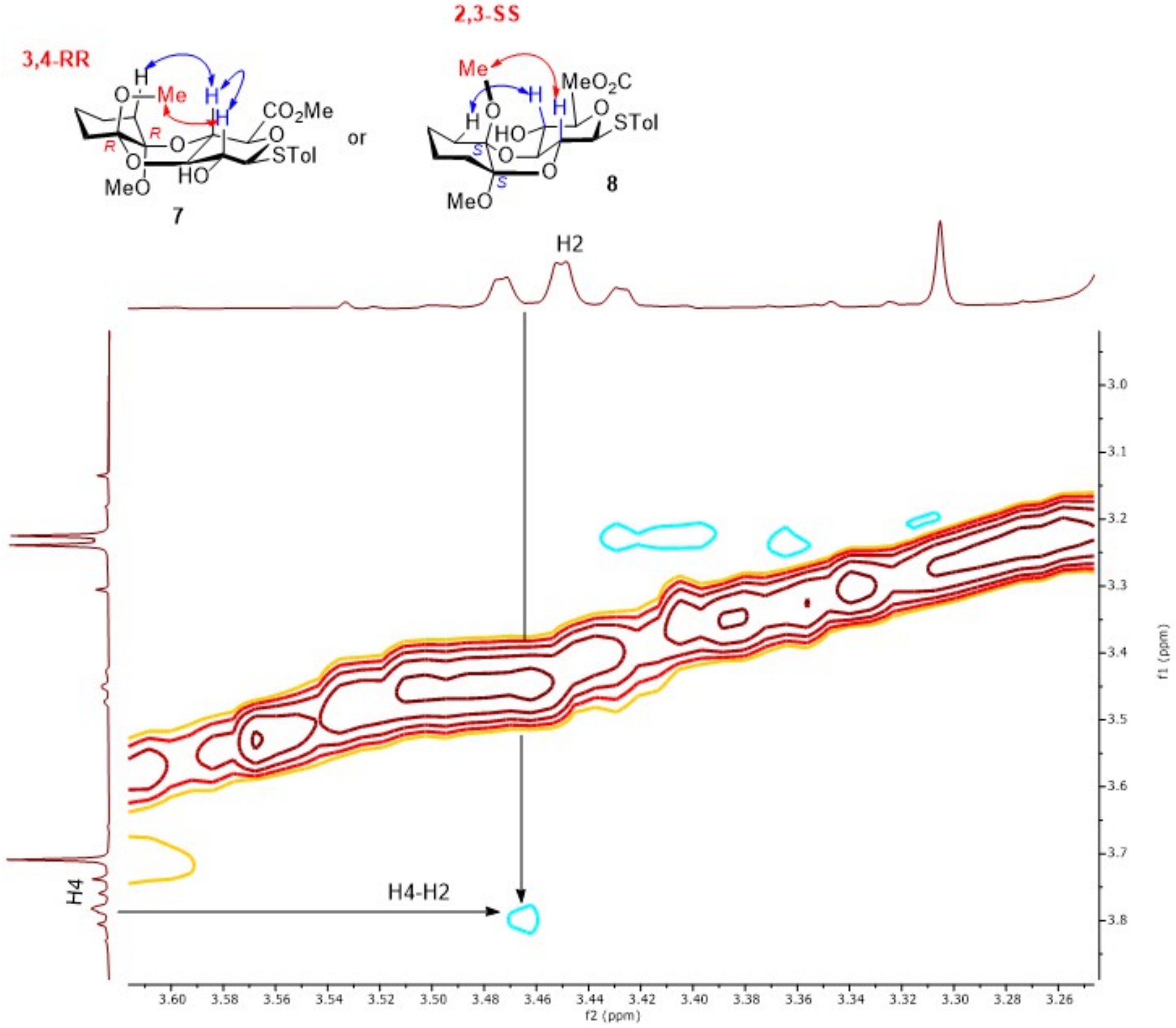

^{1}H NMR (400 MHz; $CDCl_3$) spectrum showing absence of starting material 1 after 8 hours.

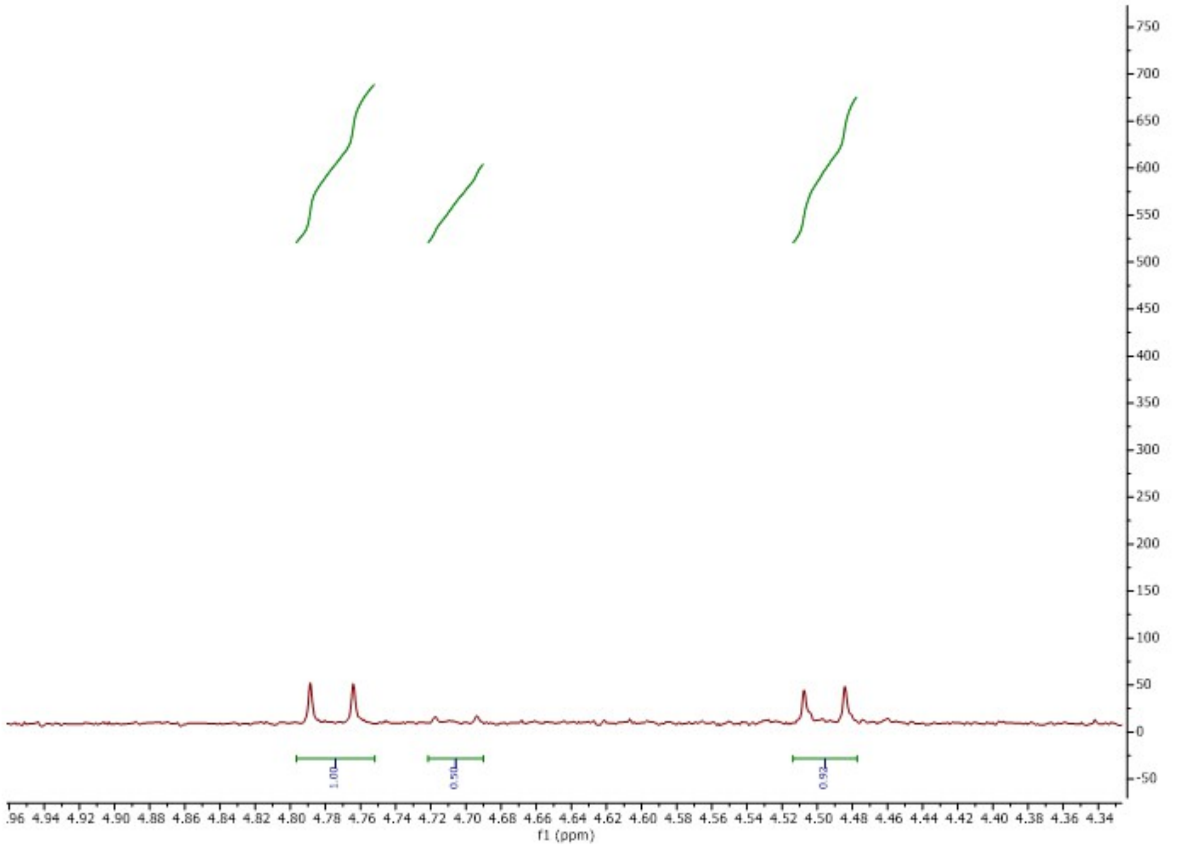

^{1}H NMR (400 MHz; $CDCl_3$) spectrum of the anomeric peak and methoxy peak integrations of the O2, O3 CDA isomer 2 after 24 h in the presence of 1,2-cyclohexanedione.

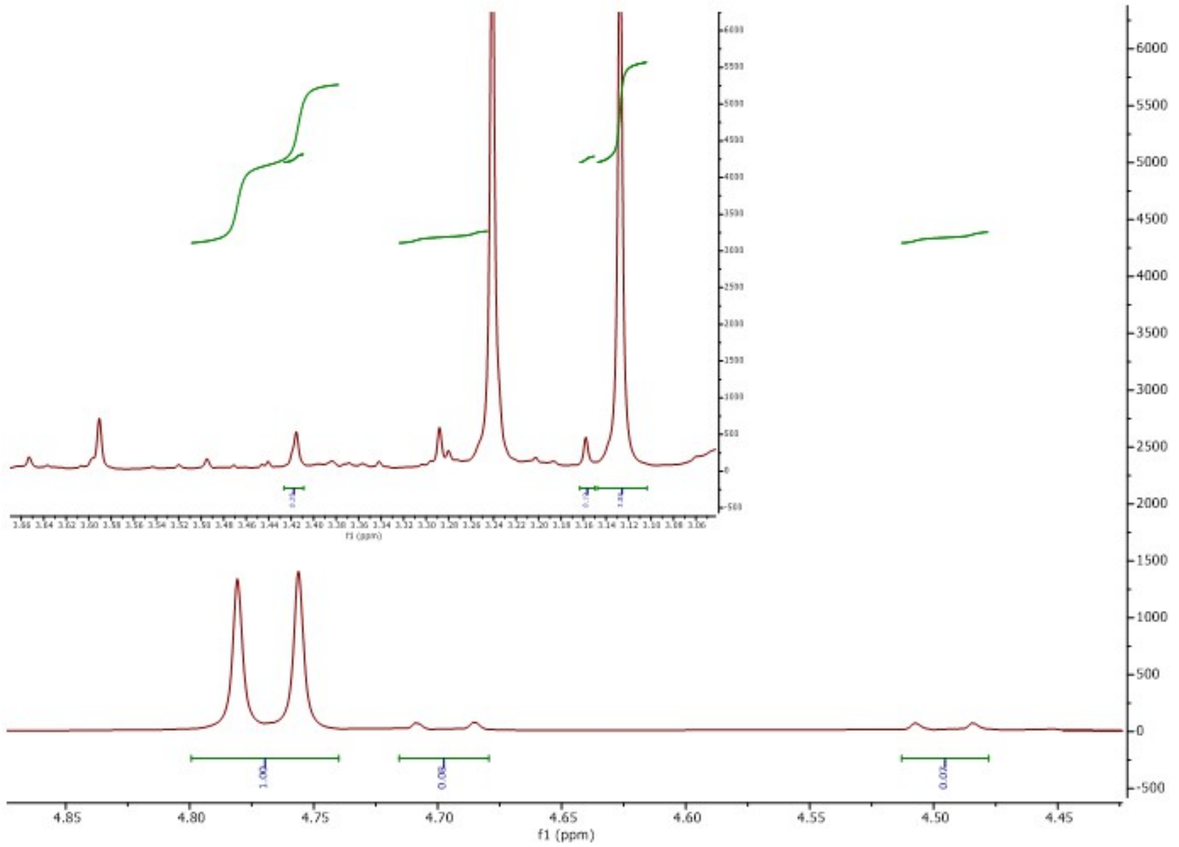

^{1}H NMR (400 MHz; $CDCl_3$) spectrum of the anomeric peak and methoxy peak integrations of isomer 2 interconversion reaction after 72 h in the presence of 1,2-cyclohexanedione.

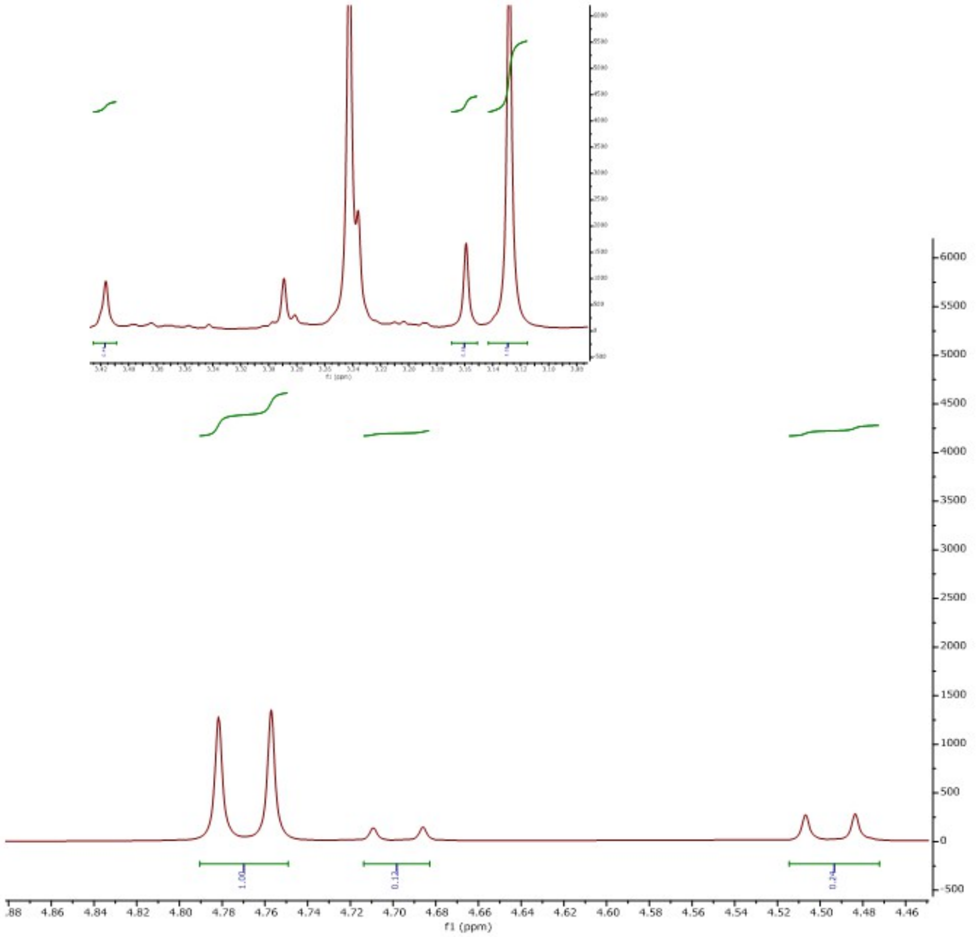

^{1}H NMR (400 MHz; $CDCl_3$) spectrum of the clean crude product of the O2,O3 CDA isomer 2 interconversion reaction after 72 h in the presence of 1,2-cyclohexanedione to show mass recovery was gotten from a clean crude product.

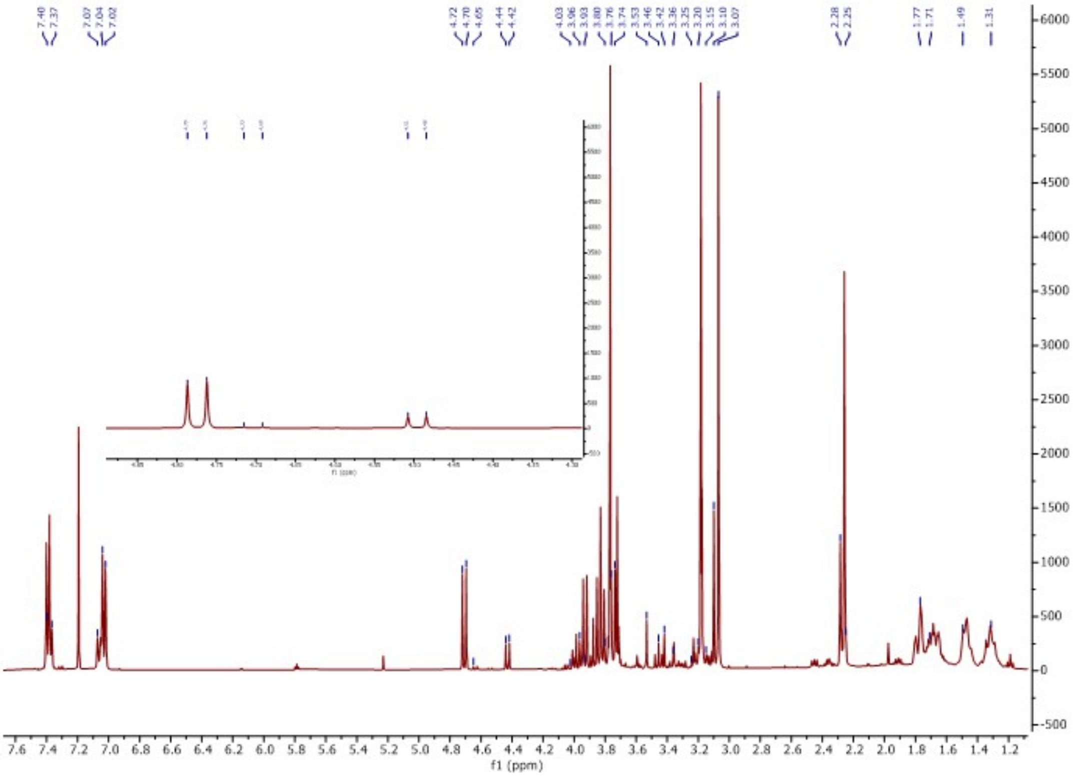

¹H NMR (400 MHz; $CDCl_3$) spectrum of the interconversion reaction of the O2, O3 CDA isomer 2 after 72 h in the absence of 1,2-cyclohexanedione.

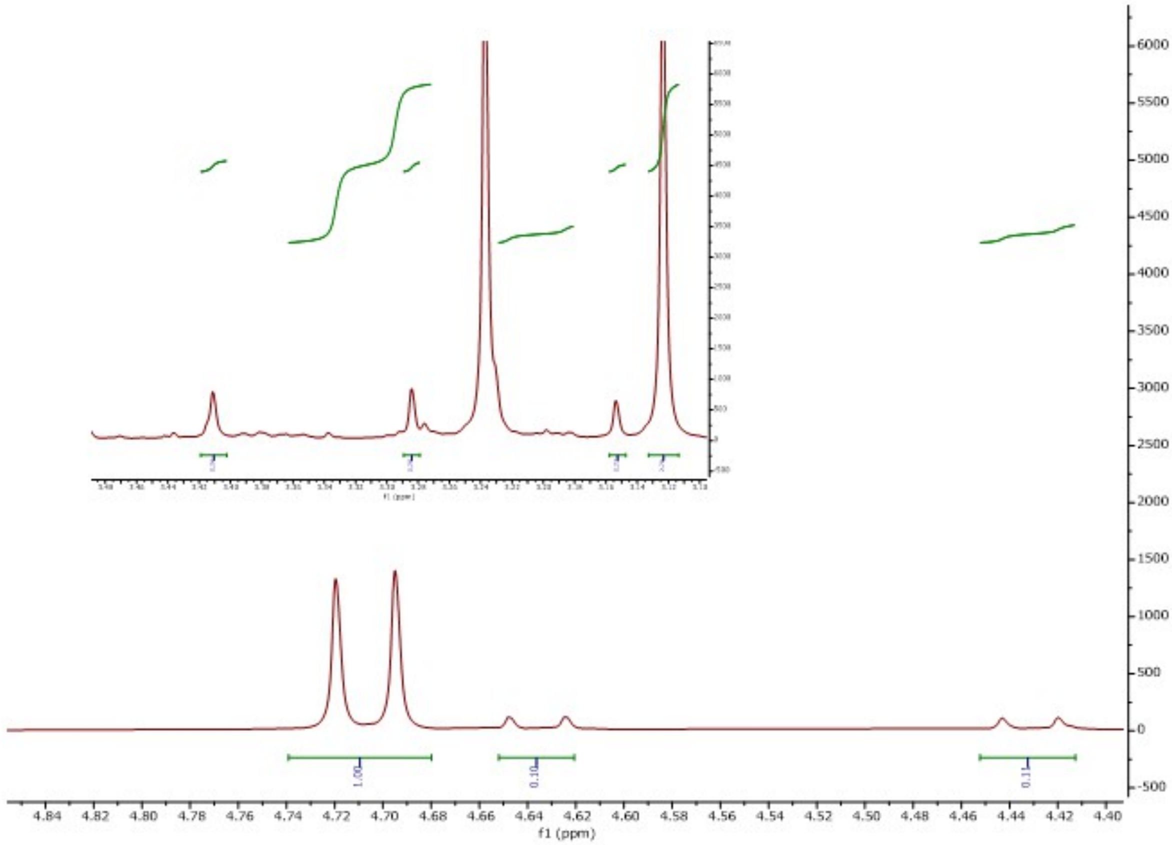

¹H-NMR (400 MHz; $CDCl_3$) spectrum of the clean crude product of the O2,O3 CDA isomer 2 interconversion reaction after 72 h in the absence of 1,2-cyclohexanedione to show mass recovery was gotten from a clean crude product.

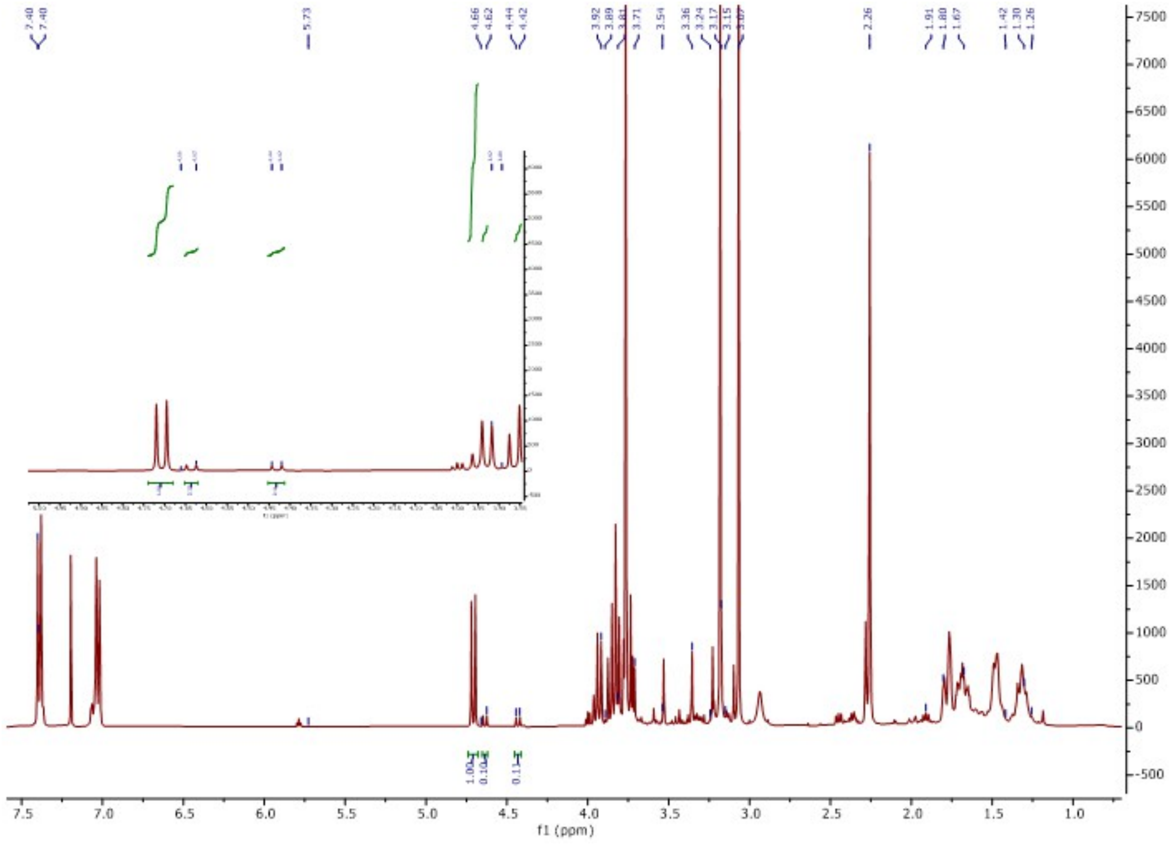

^{1}H-NMR (400 MHz; $CDCl_3$) spectrum of the O3, O4 GlcA-CDA isomer 3 interconversion reaction after 24 h in the presence 1,2-cyclohexanedione.

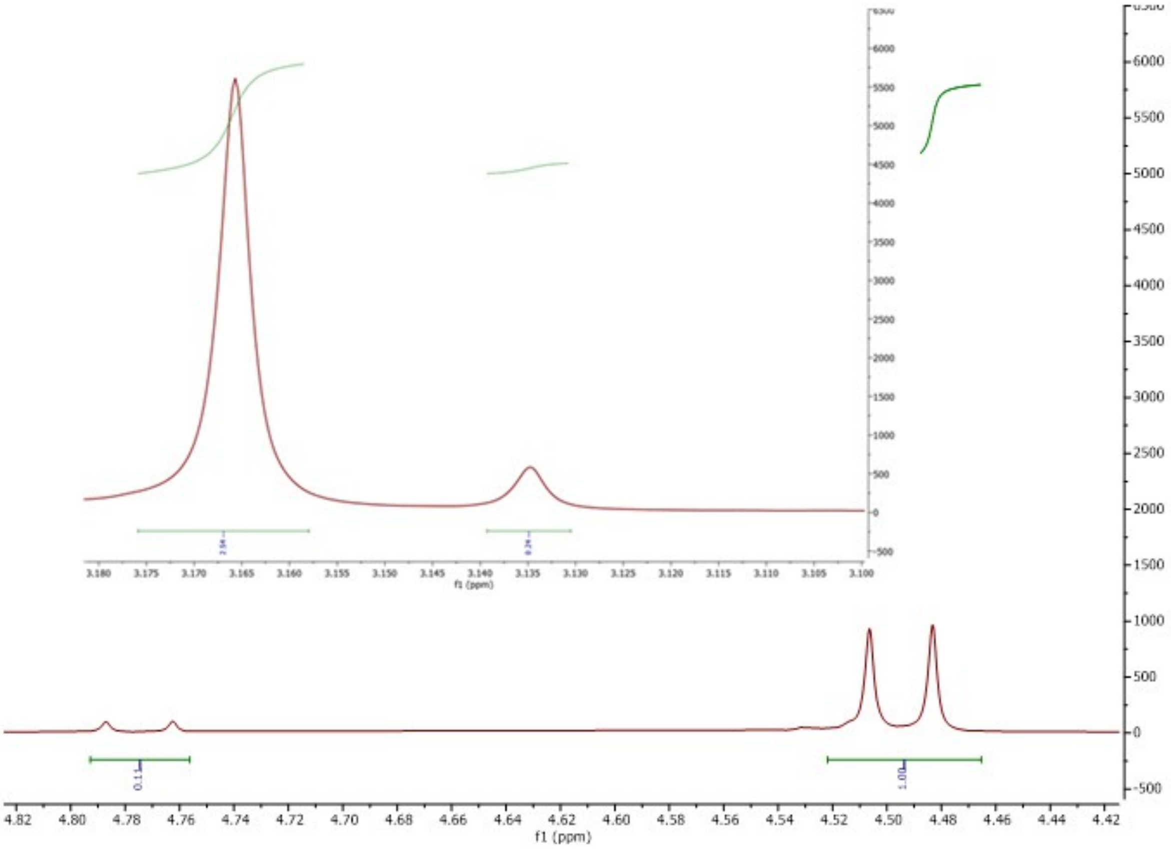

^{1}H- NMR (400 MHz; $CDCl_3$) spectrum of the O3, O4 GlcA-CDA isomer 3 interconversion reaction after 72 h in the presence 1,2-cyclohexanedione.

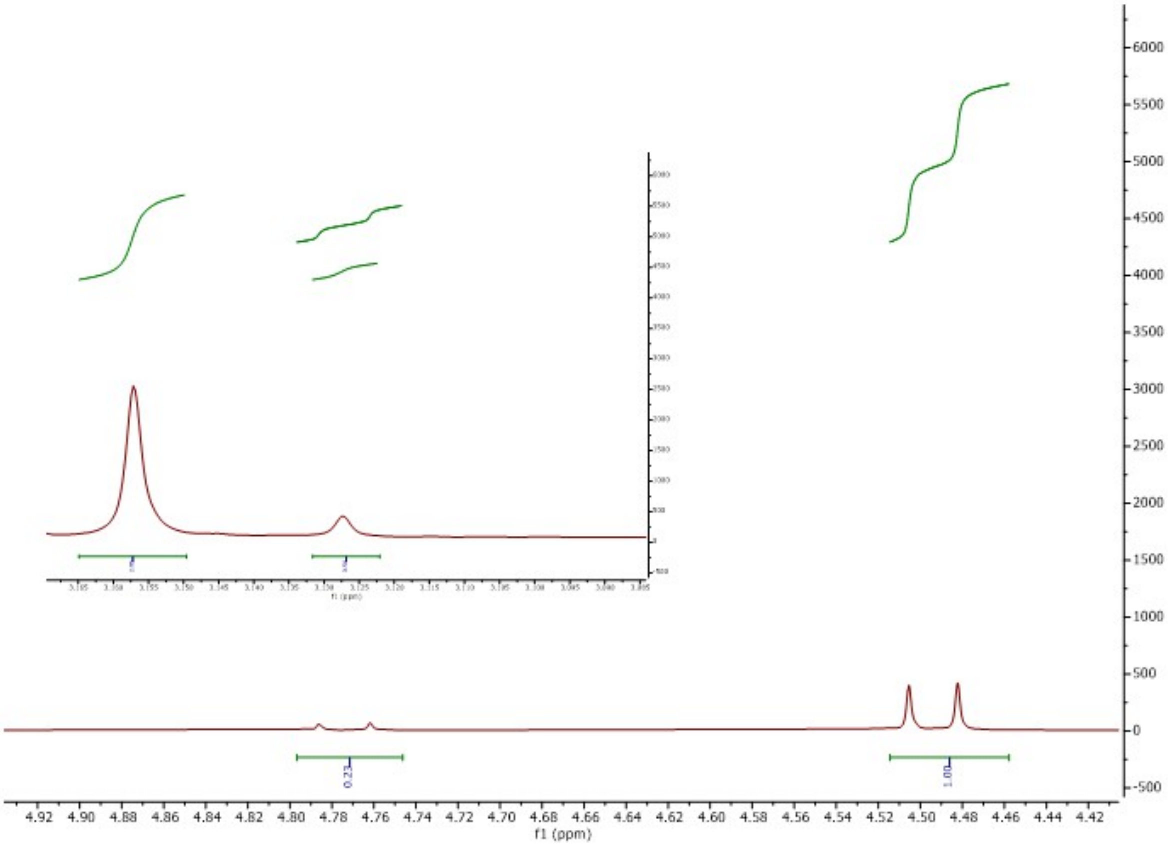

^1H NMR (400 MHz; $CDCl_3$) spectrum of the clean crude product of the O3,O4 CDA isomer 3 interconversion reaction in the presence of 1,2-cyclohexanedione after 72 h.

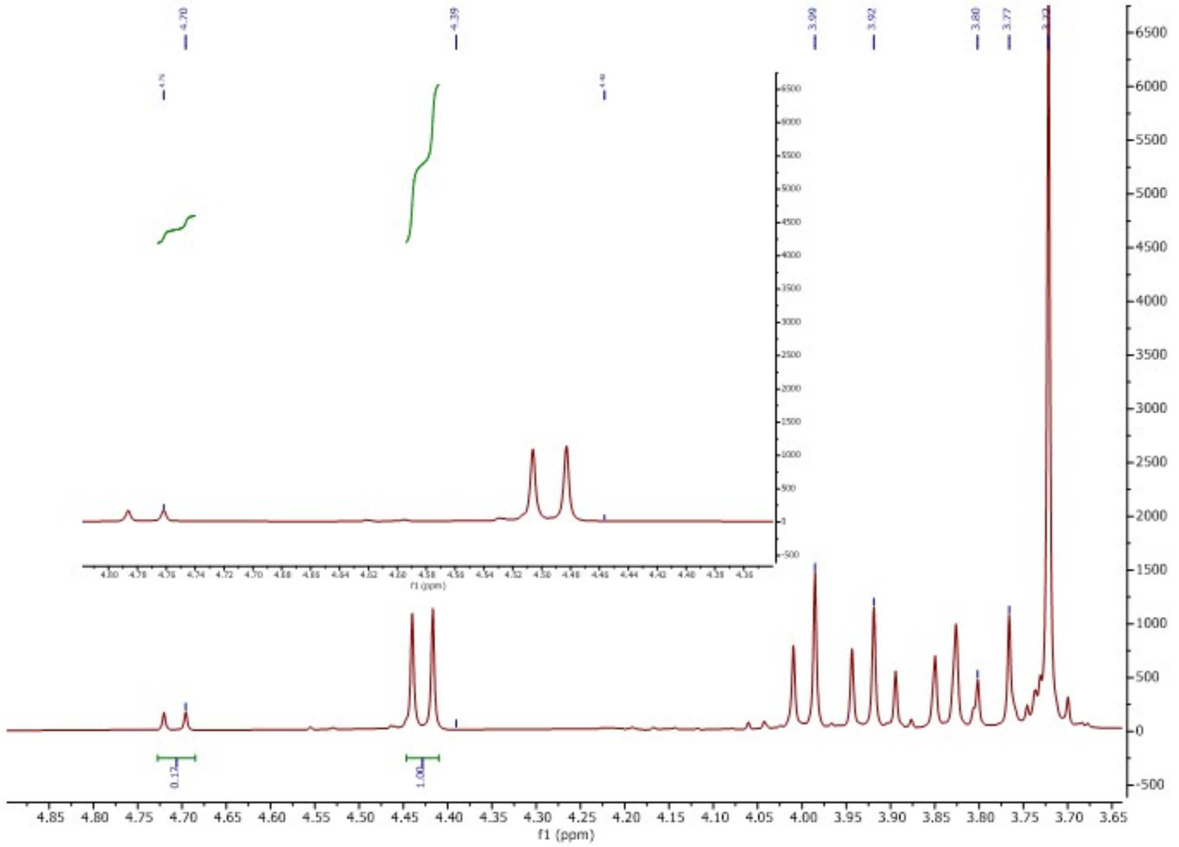

^1H NMR (400 MHz; $CDCl_3$) spectrum of the interconversion reaction of the O3, O4 CDA isomer 3 after 24 h in the absence of 1,2-cyclohexanedione.

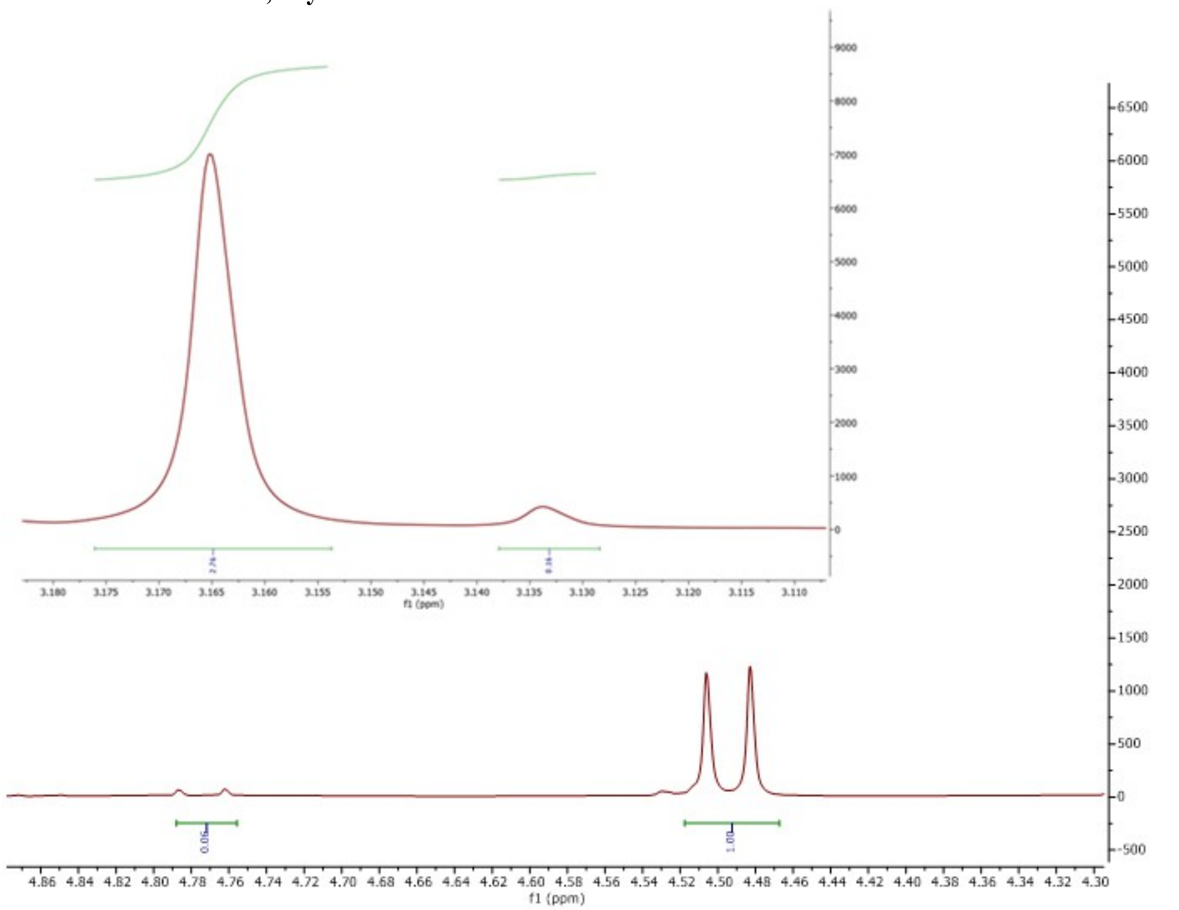

^{1}H NMR (400 MHz; $CDCl_3$) spectrum of the interconversion reaction of the O3, O4 CDA isomer 3 after 72 h in the absence of 1,2-cyclohexanedione.

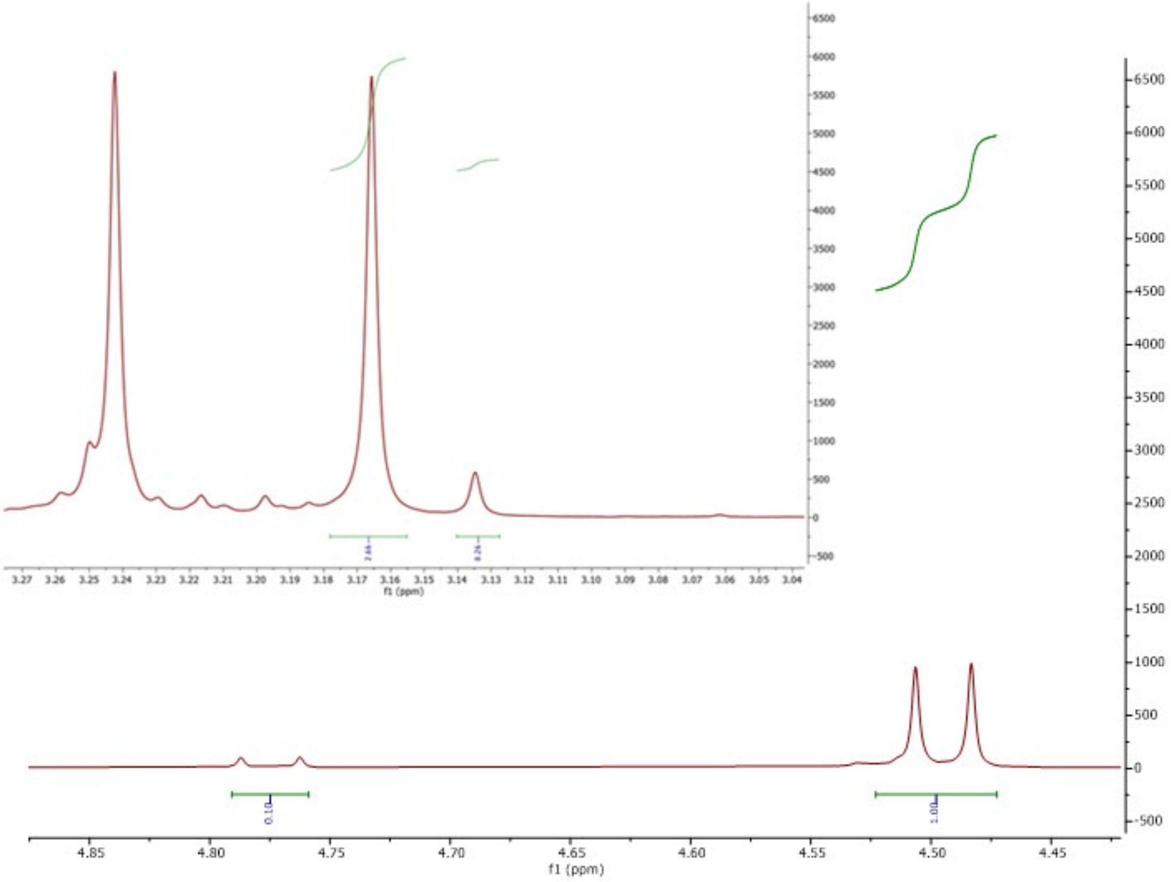

^{1}H NMR (400 MHz; $CDCl_3$) spectrum of the clean crude product of the O3,O4 CDA isomer 3 interconversion reaction in the absence of 1,2-cyclohexanedione after 72 h.

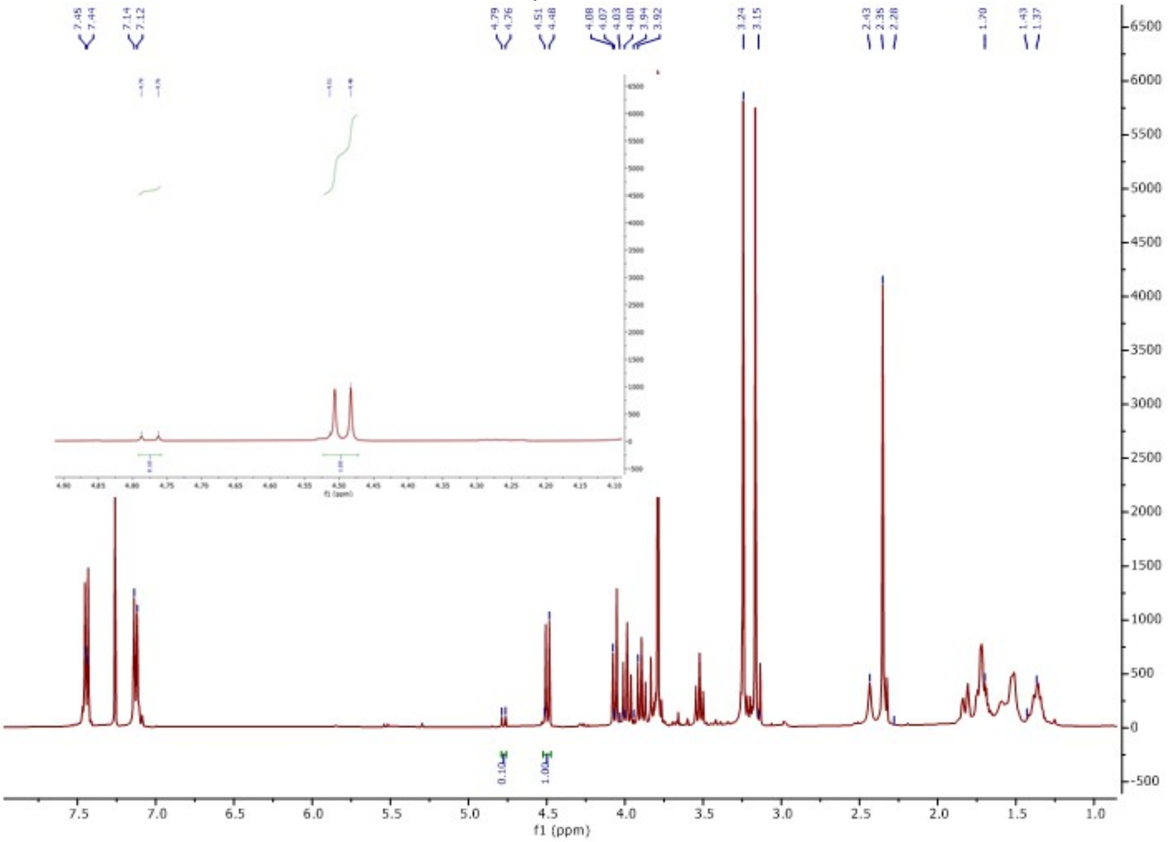

¹H NMR spectrum of the 2,3-SR isomer 4 after 72 h in the absence of 1,2-cyclohexanedione

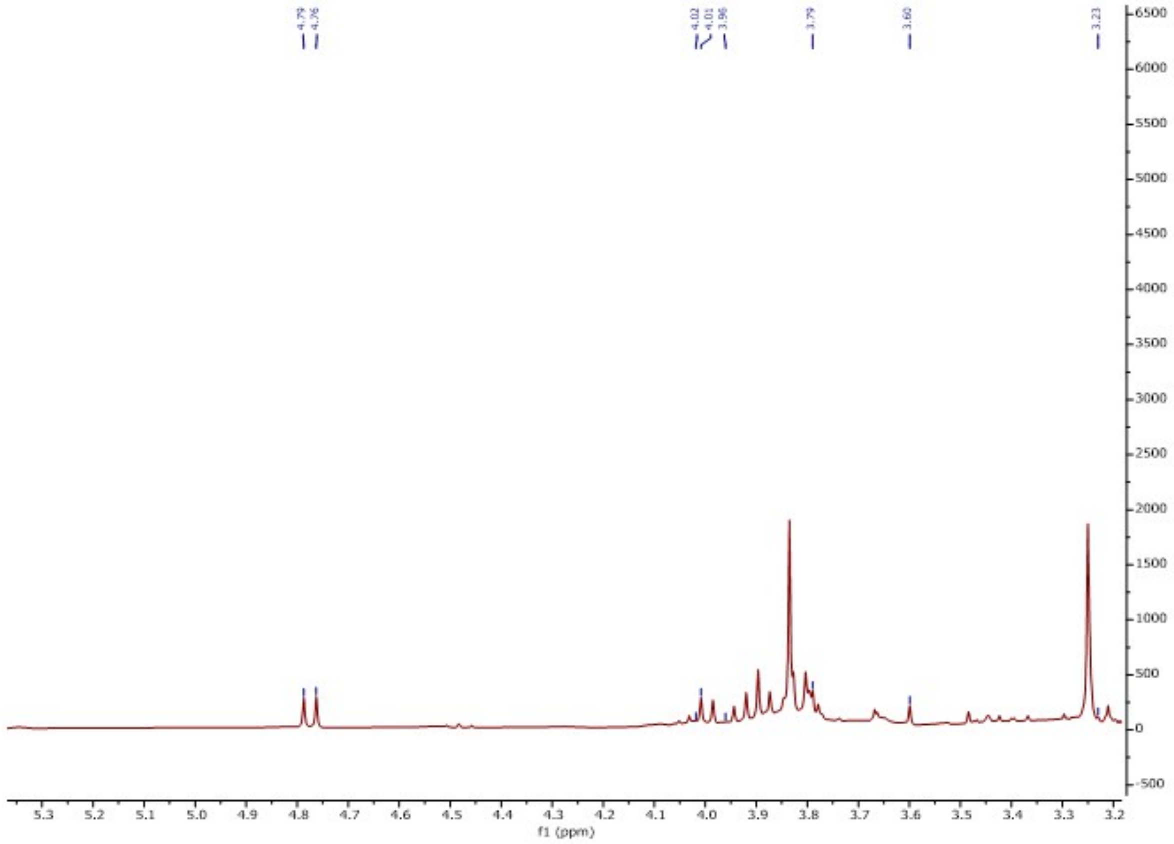

Fig S1: Alternative possible isomers

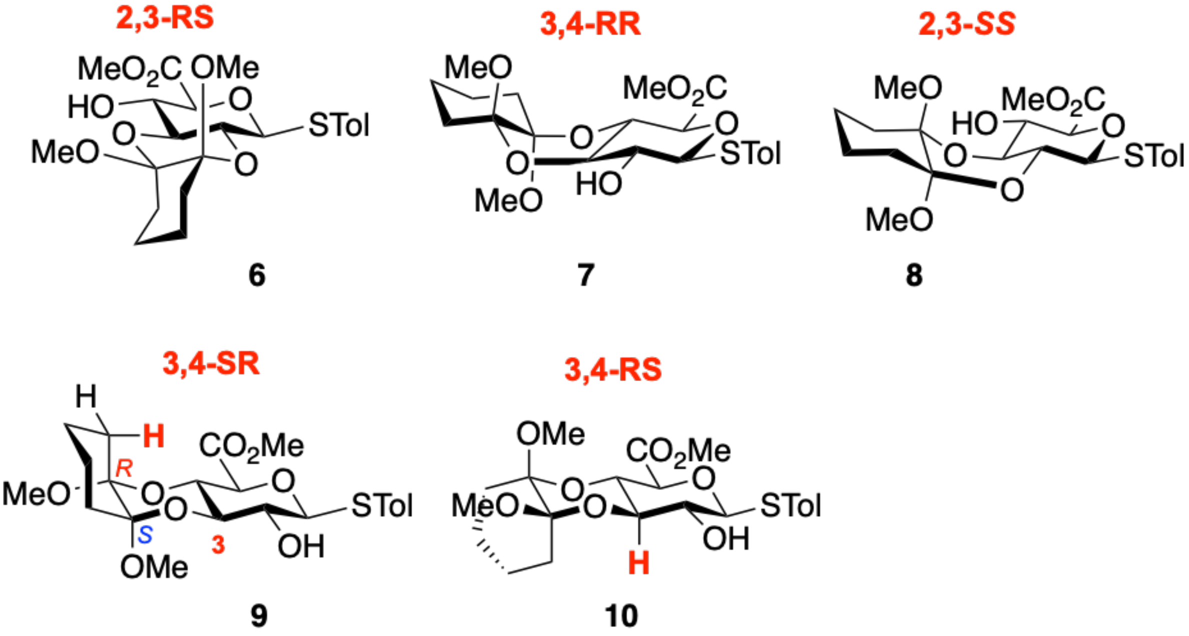

Fig S2: The anomeric signals for all three are well separated. The methyl ester methyl signal is very similar for the 3,4-SS (**3**) and 2,3-SR (**4**) isomers, however, the acetal methyls are clearly shifted downfield for the minor 2,3-SR isomer **4**.

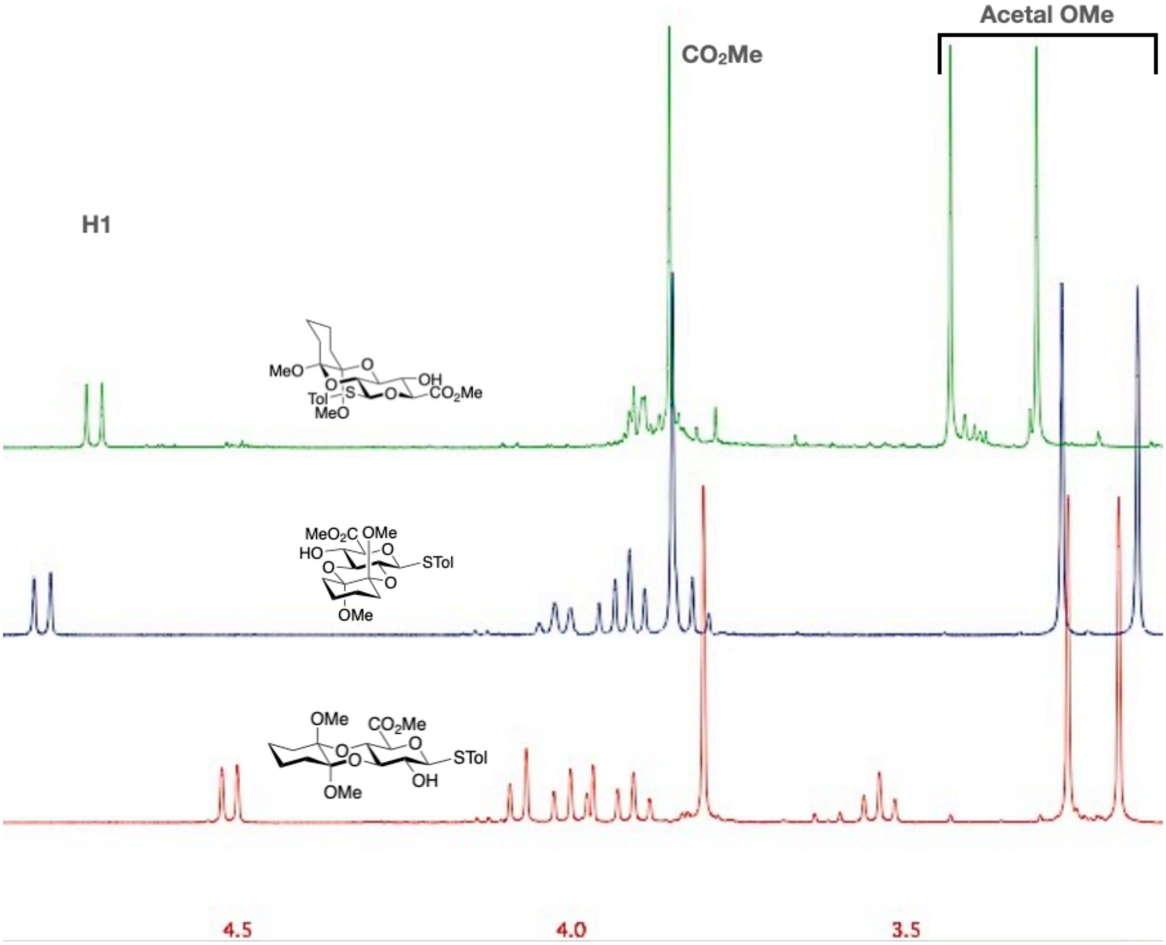

^{1}H-NMR (400 MHz, MeOD) of methyl 1-thiotolyl-β-D-glucopyranuronate 1

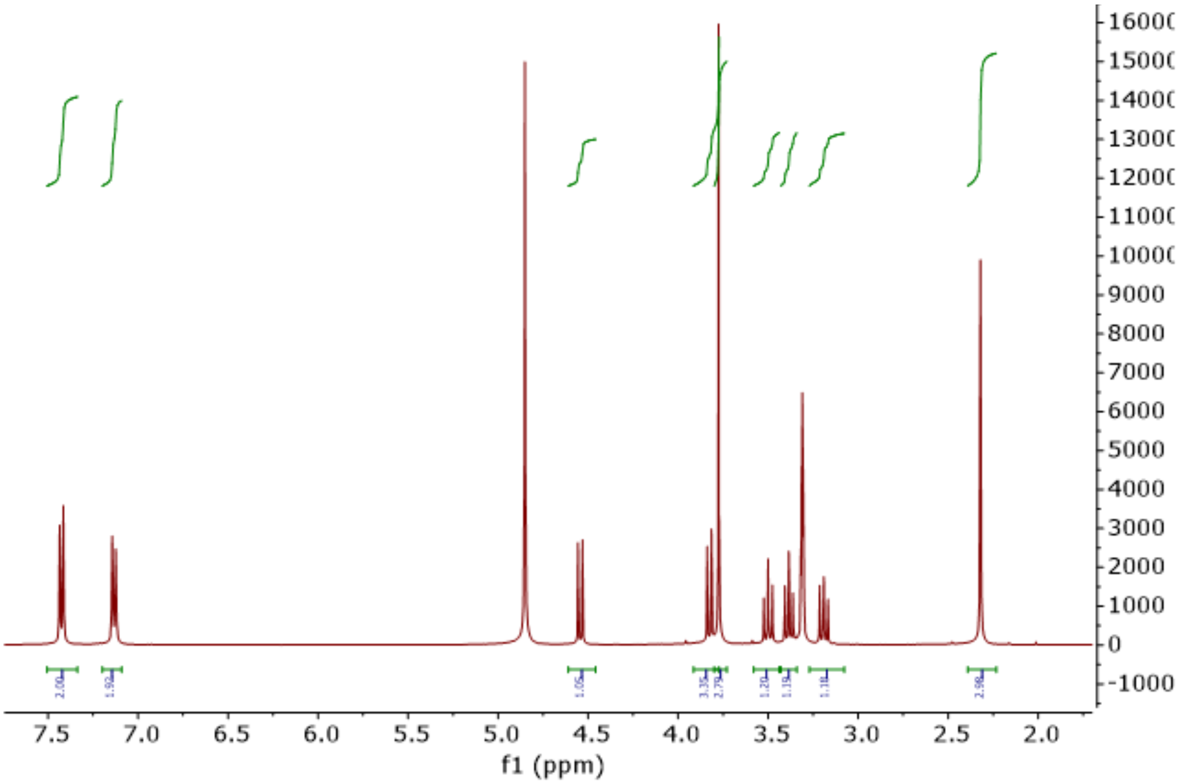

COSY NMR (400 MHz, MeOD) of methyl 1-thiotolyl-β-D-glucopyranuronate 1

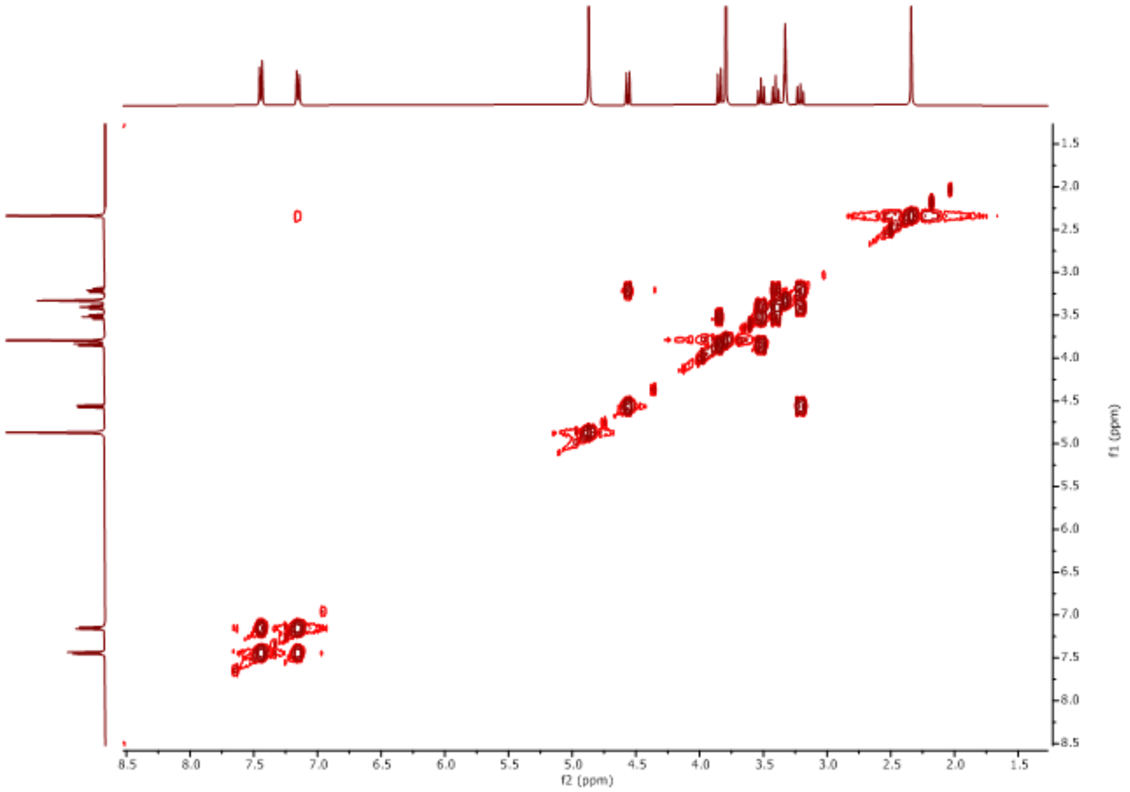

^{13}C NMR (100 MHz, MeOD) of methyl 1-thiotolyl-β-D-glucopyranuronate 1

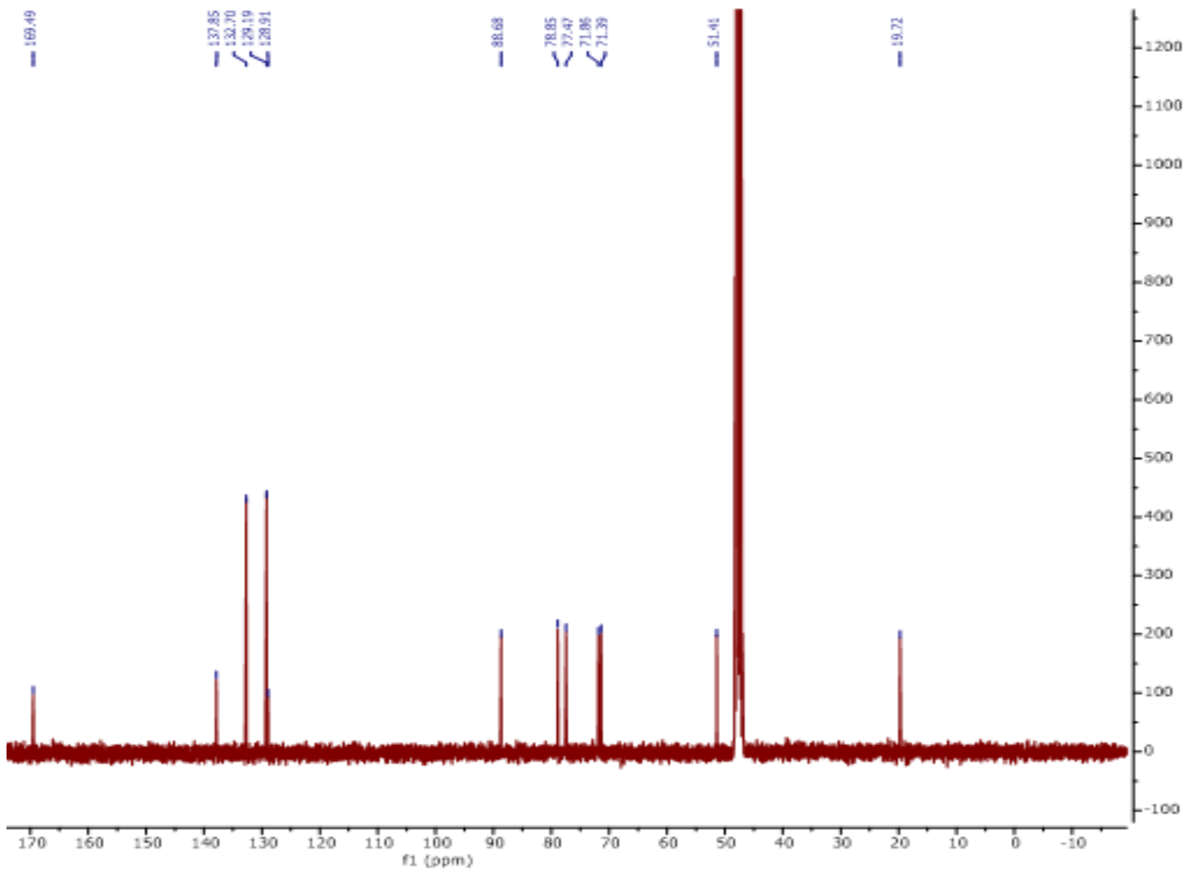

HSQC NMR (400 MHz, MeOD) of methyl 1-thiotolyl-β-D-glucopyranuronate 1

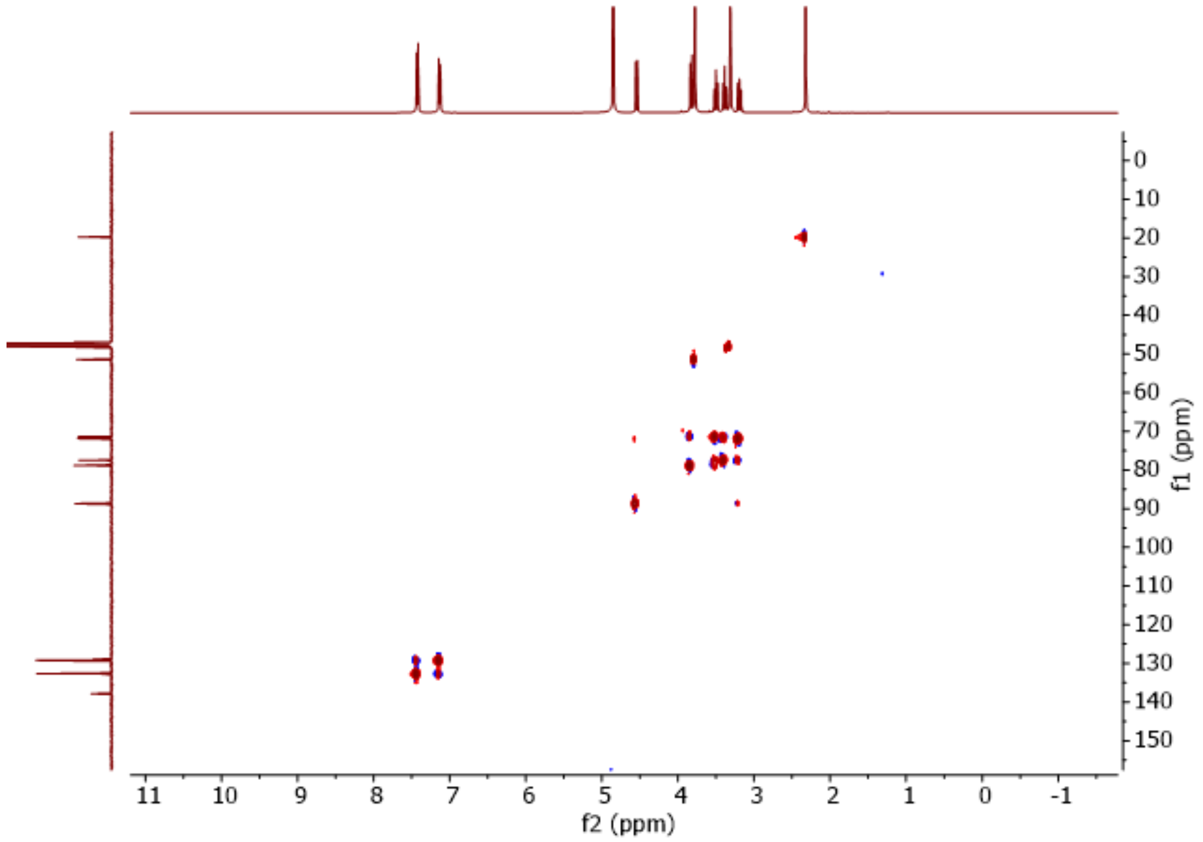

Fig S3: ORTEP at 50% probability for O4-TCA protected 2,3-CDA acetal, **5**.

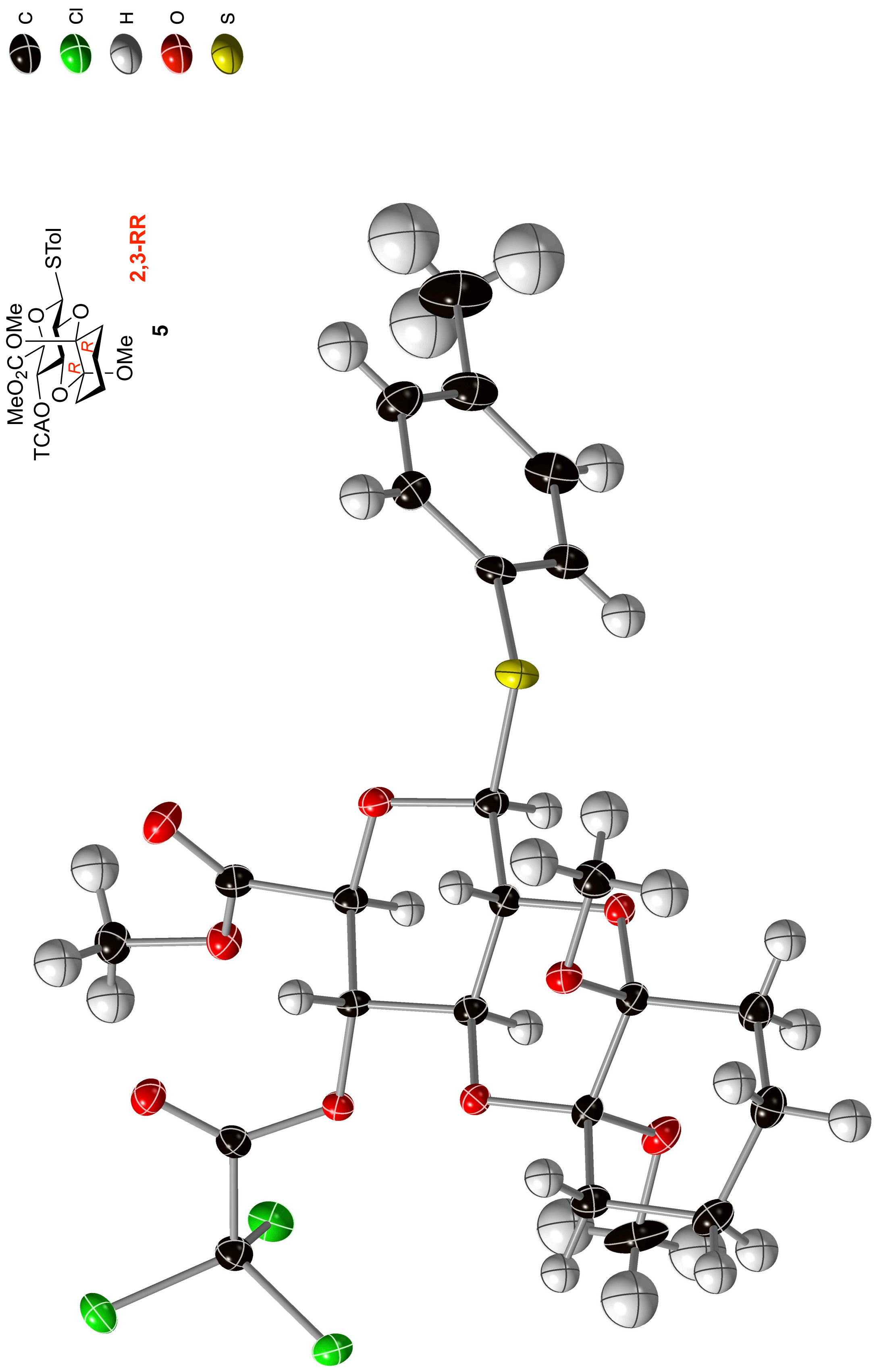

Fig S4: ORTEP at 50% probability for 3,4-CDA acetal, **3**.

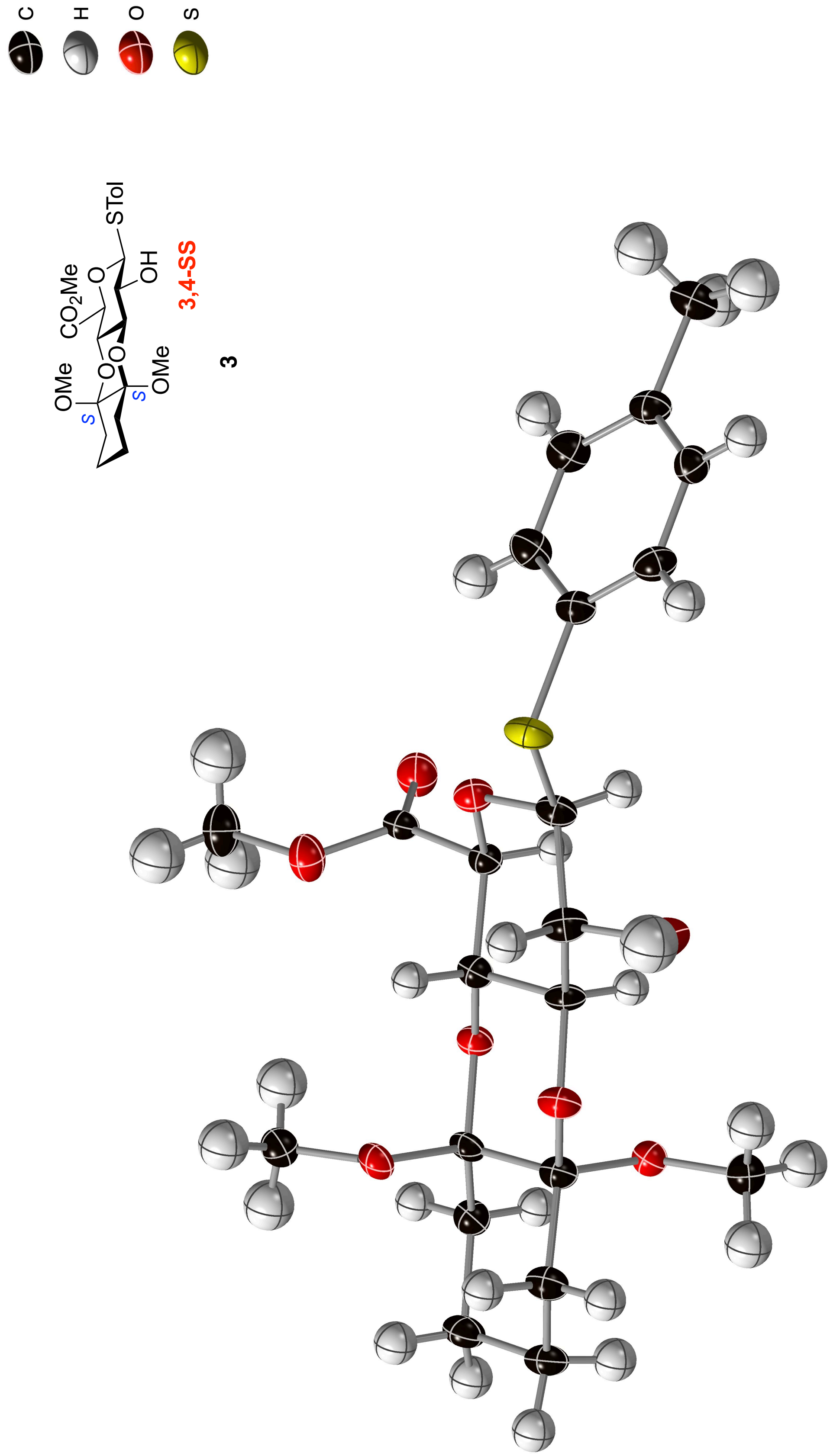

Fig S5A: ORTEP at 50% probability for minor trans-methoxy 3,4-CDA acetal, **4**. Co-crystallized with molecule of chloroform.

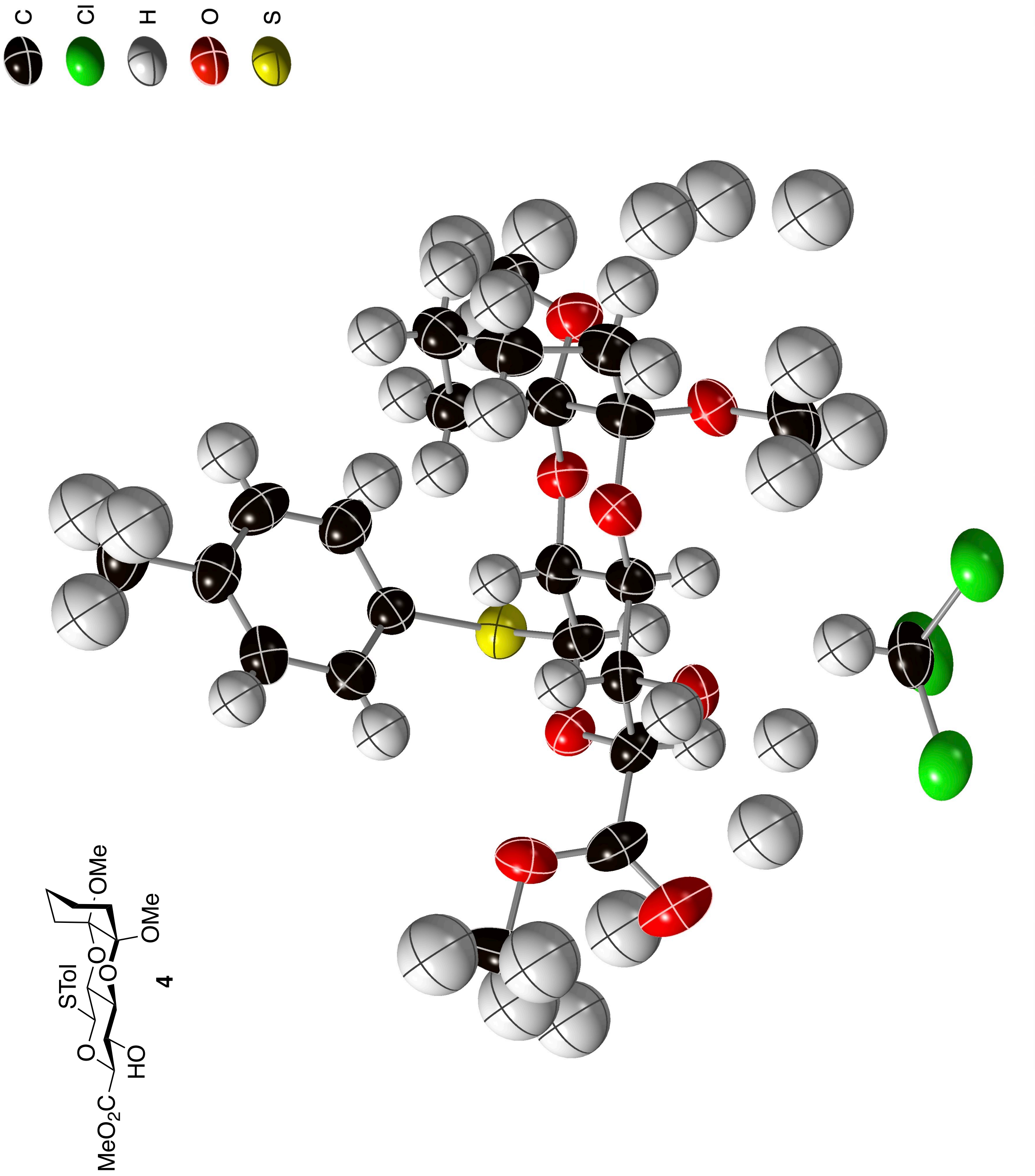

Fig S5B: ORTEP at 50% probability for minor trans-methoxy 3,4-CDA acetal, **4**. Co-crystallized with molecule of chloroform. Hydrogens omitted for clarity.

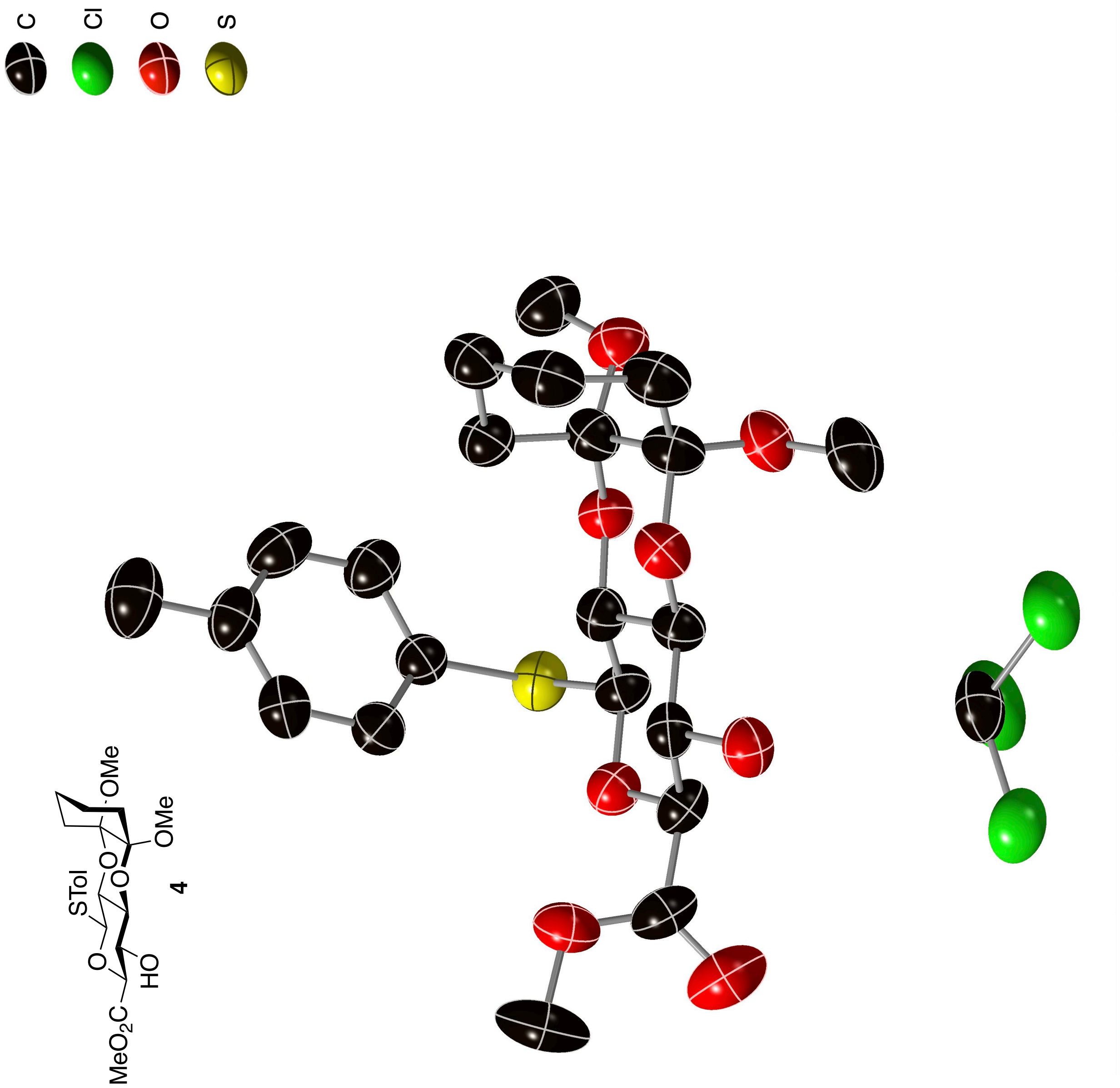

Supplement: Supplementary file 1 — ol3c00255_si_001.pdf [file ol3c00255_si_001.pdf]
